# Supplementary material for: Essential roles of B cell subsets in the progression of MASLD and HCC
Source: JHEP Rep. 2024 Aug 22;6(12):101189. doi: 10.1016/j.jhepr.2024.101189 (PMC11602976; doi:10.1016/j.jhepr.2024.101189)
Supplement: Multimedia component 1 [file mmc1.pdf]

Essential roles of B cell subsets in the progression of MASLD and HCC

Nataliia Petriv, Huizhen Suo, Inga Hochnadel, Kai Timrott, Nina Bondarenko, Lavinia Neubert, Elena Reinhard, Nils Jedicke, Patrick Kaufhold, Carlos Alberto Guzmán, Ralf Lichtinghagen, Michael P. Manns, Heike Bantel, Tetyana Yevsa

Table of contents

Abbreviations……………………………......…………………………………………..……...…2

Supplementary materials and methods……………………………......……………..…………3

Supplementary figures…………………………………………………………….....……..…8

Graphical abstract legend …….…………......…………………………………….…..…....20

Supplementary tables…………………………………………………………….….............21

Supplementary references……………………………………………………………..…...27

Abbreviations

AFP	alpha-1-fetoprotein
BMI	body mass index
Breg	B regulatory cell
BSA	bovine serum albumin
DAPI	4’,6-diamidino-2-phenylindole
DN	double-negative
EDTA	ethylenediaminetetraacetic acid
FBS	fetal bovine serum
GGT	gamma-glutamyltransferase
H&E	hematoxylin and eosin
HCC	hepatocellular carcinoma
HDI	hydrodynamic tail vein injection
HFD	high-fat diet
HRP	horseradish peroxidase
IF	immunofluorescence
IHC	immunohistochemistry
IRES	internal ribosome entry site
LmAIO	*Listeria monocytogenes* Δ*actA/*Δ*inlB + Ova*
MBCs	memory B cells
MN	mature naïve
MASLD	metabolic dysfunction-associated steatotic liver disease
NCD	normal chow diet
NSw	non-switched
Ova	ovalbumin
PBS	phosphate buffered saline
PBs	plasmablasts
PD-L1	programmed death-ligand 1
SB13	*Sleeping Beauty 13*
Sw MBCs	switched memory B cells

Supplementary materials and methods

Human samples

Fresh blood samples, obtained from the patients with metabolic dysfunction-associated steatotic liver disease (MASLD), were prepared for FACS analysis. Liver tissue samples (resected material after surgeries) were collected only from the patients with hepatocellular carcinoma (HCC). The tumor liver tissues were harvested for immunohistochemical (IHC) and immunofluorescent (IF) analyses. The prospective study using all human material (blood and liver tissues obtained after surgeries) was conducted in accordance with the Helsinki Declaration. It was approved by the Ethics Committee of the MHH (approval numbers: 3261_BO_K_2016, 7825_BO_K_2018 and 8742_BO_K_2019). The appropriate informed consent was received from all the patients.

Mouse strains, animal housing and diets used

Wild-type (WT) C57BL/6J mice were purchased from Charles River Laboratories. *P19*$^{Arf-/-}$ mice [1] were obtained in a C57BL/6J background as described previously [2, 3]. B-cell-deficient mice (B6.129S2-Igh-6$^{tm1Cgn/J}$ (µMT) [4] and B6.129P2-Igh-J$^{tm1Cgn/J}$ (JHT) [5]) were purchased from the Jackson Laboratory and were bred at the animal facility of the MHH, Hannover, Germany.

Mice were maintained on a 12 h light and dark cycle with *ad libitum* access to water and a normal chow diet composed of 10% fat. For inducing MASLD, three-weeks-old C57BL/6J female mice were fed with a 60% high-fat diet (HFD, Envigo Teklad TD06414) for 14 weeks long, as previously established [6-8].

B-cell depletion and combination treatment with α-CD20

B-cell depletion was carried out using a monoclonal α-CD20 antibody from BioLegend (clone SA271G2). Mice were treated with 250 µg α-CD20 antibody administered intraperitoneally (i.p.) twice per week [9, 10]. The combination therapy, comprising the α-CD20 treatment and a vaccination with a double-deleted vaccine strain *Listeria monocytogenes ΔactA/ΔinlB*, expressing ovalbumin (designated LmAIO), was performed in HCC-Ova mouse model induced in *p19*$^{Arf-/-}$ mice, as described previously [11]. All antibodies used in the study are listed in Table S3.

Isolation of human peripheral blood mononuclear cells (PBMCs)

Peripheral human blood was obtained by venepuncture and collected into tubes containing ethylenediaminetetraacetic acid (EDTA) as an anticoagulant. PBMCs were obtained using Ficoll-Paque PLUS (GE Healthcare, USA) density gradient centrifugation, according to the

manufacturer's instructions. Briefly, whole blood was diluted with an equal volume of PBS (at room temperature), underlain with Ficoll-Paque (at room temperature), then centrifuged (400×*g*, 30 min, 21 °C) on a Heraeus Megafuge 40R (Thermo Fisher Scientific, USA) without break. PBMCs were collected from the Ficoll-Paque-plasma interface into a 50 ml tube (Greiner Bio-One, Germany), washed twice in PBS (100×*g*, 10 min, 4 °C), and re-suspended at 10^6 cells/ml in complete RPMI 1640 (Gibco, USA). Then, the total number of cells was counted using 0.4% trypan blue (Sigma Aldrich, USA) and a cell counter (LUNA-FL, Logos Biosystems, South Korea).

Vector design and induction of HCC using hydrodynamic tail vein injection (HDI)

Sleeping Beauty 13 (*SB13*) transposase, *NRAS*^{G12V} (*CaN*), and *Myc-NRAS*^{G12V}*-IRES* (*CaMIN*) encoding transposon vectors have been described recently [2, 8, 12]. DNA vectors (transposon and transposase) for HDI were prepared using the QIAGEN EndoFreeMaxi Kit (QIAGEN, Hilden, Germany). For transposon-mediated gene transfer, 4- to 6-week-old animals received a 25 µg:5 µg ratio of transposon to transposase-encoding plasmid, as previously described [2, 3, 13]. DNA was diluted in saline solution at a final volume of 10% of body weight. The model of transposon-mediated stable intrahepatic transfer of oncogenic *NRAS*^{G12V} *or Myc-IRES-NRAS*^{G12V} were used to induce HCC development in *p19*$^{Arf-/-}$ or C57BL/6J mice respectively [1-3, 8]. HCC-Ova was induced in *p19*$^{Arf-/-}$ mice, as previously described [11].

Leukocyte isolation from murine liver tissues and blood

Single-cell suspensions from the murine livers were prepared as previously described [3, 8, 13]. Briefly, liver tissue was chopped into small ~1 mm^3 pieces and then enzymatically digested in complete DMEM medium (Gibco, USA) supplemented with 5% fetal bovine serum (FBS, Roche, Switzerland), 0.5 mg/ml collagenase D (Roche, Switzerland) and 0.01 mg/ml DNAse I (Sigma Aldrich, USA) for 25 min at 37 °C. After incubation, the enzymatic reaction was stopped using 5 mM EDTA and, the liver suspension was passed through a 100 µm cell strainer. Erythrocytes were lysed using an ACK buffer (150 mM NH_4Cl, 10 mM $KHCO_3$, and 0.1 mM EDTA) [3].

Blood was collected from the retro-orbital plexus and mixed with 50 units of heparin (Ratiopharm GmbH, Germany) and erythrocytes were lysed using ACK buffer. The obtained single-cell suspensions were used for stainings and multicolor flow cytometry (FACS) analysis.

Cell suspension staining and multicolor FACS analysis

Isolated single-cell suspensions were stained in 96-well U-bottom plates with fluorochrome-conjugated monoclonal antibodies in FACS buffer (PBS supplemented with 2% FBS, pH=7.4). To avoid Fc-mediated nonspecific interactions, murine cells were pre-incubated with the anti-CD16/32 (clone 93, BioLegend, USA), and human cells were incubated with FcR blocking reagent (BioLegend, USA). Dead cells were excluded based on staining with the Alexa Fluor 350 NHS Ester (Life Technologies, USA). For staining of murine samples, the following monoclonal antibodies were used: anti-B220 (clone RA3-6B2), anti-CD5 (clone 53-7.3), anti-CD1d (clone 1B1), anti-PD-L1 (clone 10F.9G2), anti-IgM (clone RMM-1), anti-CD1d (clone 1B1), anti-IgD (clone 11-26c.2a), anti-CD27 (clone LG.3A10), anti-CD138 (clone 281-2), anti-CD267 (clone 8F10), anti-IL-10 (clone JES5-16E3), anti-CD19 (clone 1D3), anti-IgA (clone 11-44-2).

The antibodies used for the human PBMC staining were the following: anti-CD3 (clone SK7), anti-CD11c (clone 3.9), anti-CD19 (clone HIB19), anti-CD20 (clone 2H7), anti-CD5 (clone L17F12), anti-CD1d (clone 51.1), anti-CD27 (clone M-T271), anti-IgM (clone MHM-88), anti-PD-L1 (clone 29E.2A3), anti-IL-10 (clone JES3-9D7), anti-CD45 (clone HI30), anti-IgD (clone IA6-2). All further information about the antibodies, including the corresponding purchasers, are listed in Table S3.

Samples were acquired on LSR II flow cytometer (BD Biosciences, USA) using BD FACS Diva software (Becton Dickinson Ltd, Oxford, UK) and/or a Cytek Aurora flow cytometer using a SpectroFlo software (Cytek, USA) and analyzed using a FlowJo software (Tree Star, Becton, Dickinson & Company, USA).

Histopathological examination

Murine and resected human liver tissue samples were fixed in 4% paraformaldehyde at room temperature for 24-48 hours, embedded in paraffin, and serially sectioned at 4 μm using a rotary microtome (Microm, Germany). The obtained tissue sections were stained with H&E (Sigma Aldrich, USA and Merck, Germany), argentum (Honeywell-Fluka, USA), and Sirius red (Sigma Aldrich, USA) for the histopathological evaluation, as described [14, 15].

For detection of lipid accumulation in hepatocytes, snap-frozen murine liver tissue samples were sectioned at 6 μm and stained with Oil Red O (Sigma Aldrich, USA), following the routine standard operating procedures at the Institute of Pathology at the MHH [16]. For quantitative morphometry of positive oil area (%, Oil Red O) and collagen deposition (%, Sirius red) representative images of at least five random fields (100x magnification) were calculated using the ImageJ software.

The immune infiltrates were evaluated at the entire liver tissue on the slide for the presence of lymphocytic immune cells that were scored following the recommendations of Sia D. *et al*

using the scoring from 0 to 4 as follows: 0 (absence of immune cell infiltration), 1 (minimal infiltration), 2 (mild infiltration), 3 (moderate infiltration), and 4 (strong infiltration) [17, 18]. In the present study, we characterized samples as the ‘non-inflamed’ HCC if the sample’s score was between 0 to 2 (minimal to mild immune cell infiltration) and the ‘inflamed’ HCC, if the immune infiltrate score reached levels 3 and 4 (moderate to strong immune cell infiltration).
Liver specimens were analyzed using a standard bright field microscopy (BX51, Olympus (Olympus, Japan) and a Nikon Eclipse Ti2 inverted microscope (Nikon, Japan)). The histopathological analyses were confirmed by two experienced pathologists.

Immunohistochemistry (IHC) analysis

IHC staining was performed on 4 μm-thick formalin-fixed paraffin-embedded tissue sections after heat-induced antigen retrieval in a decloaking chamber (Water Bath 1008, GFL) using citrate buffer (10 mM sodium citrate and 0.05% Tween 20, pH=6.0). Incubation with the primary antibodies to anti-CD19 (1:750, Invitrogen, USA), anti-CD5 (RTU, Epredia, USA), anti-CD1d (1:1200, antibodies-online, Germany), anti-IgD (1:1000, Abcam, USA), anti-PD-L1 (1:800, Invitrogen, USA), anti-IL-10 (1:500, Invitrogen, USA) was carried out in 5% bovine serum albumin (BSA, Carl Roth, USA) at room temperature for 1 h. Detailed information about all antibodies used for the IHC staining is provided in Table S3. Signal amplification and colour development via diaminobenzidine were performed using Epredia™ UltraVision™ Quanto Detection System horseradish peroxidase (HRP) DAB (Fisher Scientific, USA) and a counter-staining with Mayer’s hematoxylin (Carl Roth, USA).
Images were analyzed using the Nikon Eclipse Ti2 inverted microscope (Nikon, Japan). The quantification of CD19, CD5, CD1d, IgD, PD-L1, and IL-10 positive immune cells was performed using at least five representative fields (100X) and presented as a cellular density (number of positive cells per 1 mm^2).

Immunofluorescent (IF) staining of the frozen liver tissue sections

Frozen 7 μm-thick liver tissue sections were fixed in ice-cold acetone for 10 min and permeabilized using the 0.1% Triton X-100 (Sigma Aldrich, USA) and 0.1% sodium citrate dihydrate (Fisher Scientific, USA) for 2 minutes at 4°C. Sections were blocked with a PBTB buffer (PBS supplemented with 0.2% Triton X-100 (PBT), 0.2% BSA (Carl Roth, USA), 5% normal goat serum (Abcam, USA) for 30 min at room temperature and incubated, as previously described [8]. Human liver sections were stained with a mix of primary antibodies for 30 min at room temperature: anti-CD19 (clone 6OMP31, Invitrogen, USA), anti-CD5 (clone UCH-T2, Santa Cruz, USA), anti-CD1d (polyclonal, antibodies-online, Germany), anti-IgD (clone EPR6146, Abcam, USA), anti-IgM (clone R1/69, Santa Cruz, USA), anti-IL-10

(polyclonal, Abcam, USA), anti-PD-L1 (polyclonal, Invitrogen, USA). All information about the antibodies, including the corresponding purchasers, are listed in Table S3.

The secondary, fluorescent-conjugated antibodies: Alexa Fluor 488-conjugated donkey anti-rat, Alexa Fluor 555-conjugated donkey anti-rabbit, and Alexa Fluor 647-conjugated donkey anti-mouse antibody (all used at 1:400 dilution, Invitrogen) were applied for 1 h at room temperature. Thereafter, sections were counterstained with 4',6-diamidino-2-phenylindole (DAPI, 0.1 µg/ml, Sigma-Aldrich) in PBS for 10 min at room temperature. Finally, samples were mounted using Citifluor AF1 mountant solution (Electron Microscopy Sciences, Hatfield, PA, USA). Fluorescent images were analyzed using a Zeiss LSM 700 confocal laser-scanning microscope (Carl Zeiss, Germany) (40x objective), ZEN 2011 software and ImageJ software.

ELISA

IgG, IgA, IgM, and IgD levels of antibodies were analyzed in the plasma of mice according to standard ELISA protocols described previously [11]. Briefly, flat-bottomed 96-well ELISA plates were coated overnight with the corresponding antigen dissolved in carbonate buffer. Thereafter, plates were blocked with 3% BSA/PBS and supplied afterwards with murine plasma for 1 hour at 37°C. Detection was performed using anti-mouse IgM-biotin (Sigma Aldrich, USA), anti-mouse IgG-biotin (Sigma Aldrich, USA), or anti-mouse IgA-biotin (Southern Biotech, USA) followed by the incubation with streptavidin-conjugated HRP (BD Pharmingen, USA). HRP substrate solution 2,2'-azino-bis (3-ethylbenzothiazoline-6-sulphonic acid, Sigma Aldrich, USA) was added for the detection of bound IgG/IgM/IgA. Mouse IgD was detected using a sandwich ELISA Kit (CusaBio, USA). Samples were measured at the optical density of 405 nm (OD_{405}) using Synergy 2 microplate reader (BioTek, Winooski, Vermont, USA). All samples were examined in triplicates. Software Gen5 was used for the data analysis. All reagents used for ELISA analyses are listed in Table S3.

Statistical analysis

Graph design and statistical analyses were performed using GraphPad Prism 8.3 software (GraphPad Prism, San Diego, USA). The normality of the calculated variables was assessed by using the Kolmogorov–Smirnov test. To compare means, the nonparametric Mann–Whitney *U*-test was used for non-normally distributed data, whereas the unpaired Student's *t* test was applied for normally distributed data. The relationship of variables was analyzed by the unpaired Student's *t* test and Mann–Whitney nonparametric test. If not stated otherwise, the data are shown as mean +/- standard error of the mean (SEM). *P* values < 0.05 were considered statistically significant (*$p \leq 0.05$, **$p \leq 0.01$, ***$p \leq 0.001$, and ****$p \leq 0.001$).

Supplementary figures

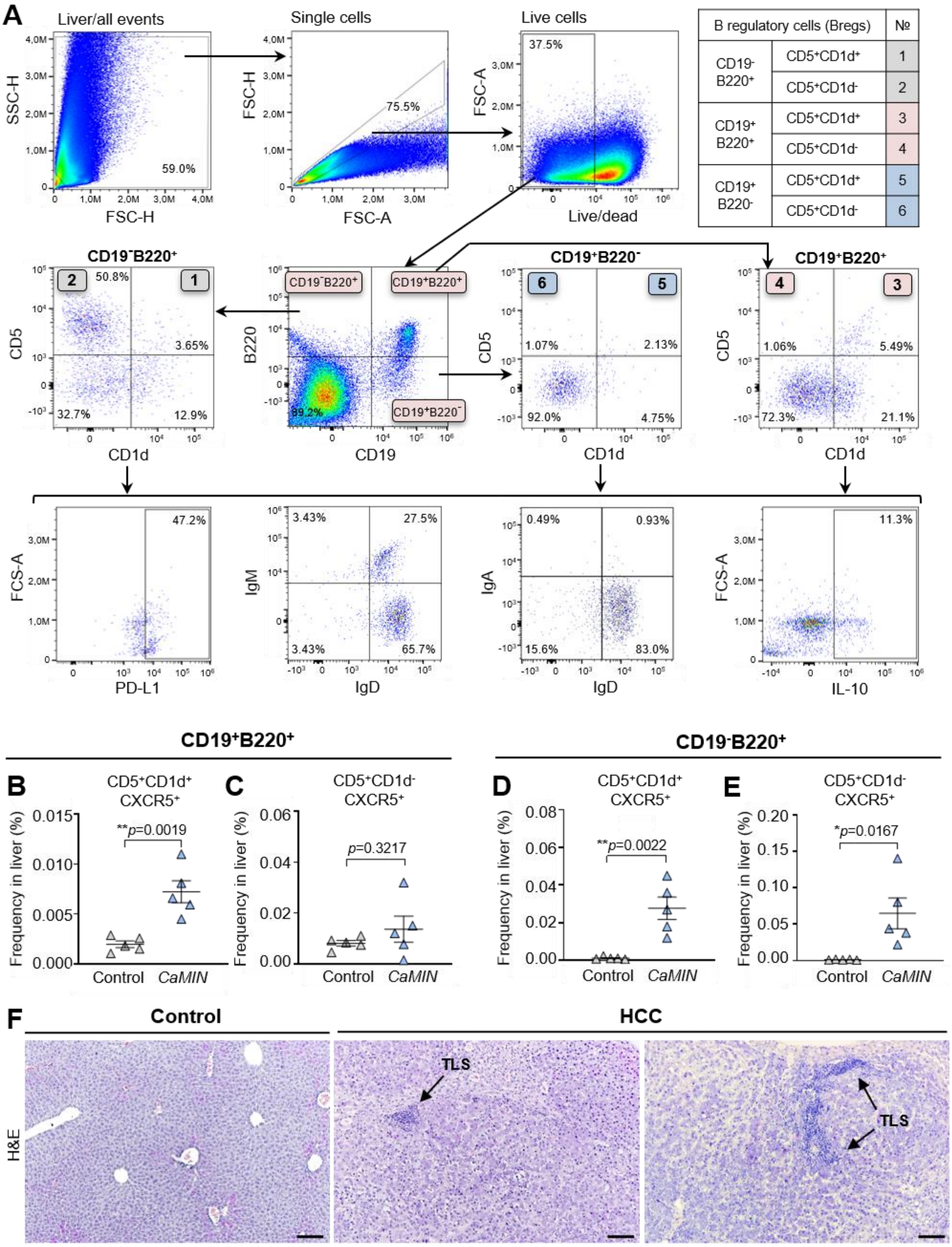

Fig. S1. Gating strategy to phenotype and characterize Bregs; elevated numbers of CXCR5⁺ Bregs and presence of TLS structures in murine HCC. (A) Gating strategy to define and characterize various populations of Bregs. Single-cell suspensions were obtained from the livers and blood of mice with MASLD, HCC/*NRAS*^{G12V}/*p19*$^{Arf-/-}$, and HCC/*CaMIN*.

The cell suspensions were stained and analyzed via the indicated surface and intracellular markers using multicolor FACS. Based on CD19, CD20, CD5, and CD1d markers six distinct populations of Bregs were identified. Further, the immunomodulatory properties of Bregs were assessed while checking PD-L1 and IL-10 expression. In addition, based on IgM, IgD, and IgA expression, several subpopulations of Bregs were defined. **(B-E)** Bregs were further checked for the presence of the homing receptor $CXCR5^+$ in the livers of HCC/*CaMIN* mice: frequencies of $CXCR5^+$-expressing **(B)** $CD19^+B220^+CD5^+CD1d^+$, **(C)** $CD19^+B220^+CD5^+CD1d^-$, **(D)** $CD19^-B220^+CD5^+CD1d^+$ and **(E)** $CD19^-B220^+CD5^+CD1d^-$ Bregs. **(F)** TLS assessment in H&E-stained murine HCC liver (magnification 100x; scale bar, 100 µm). The data were analyzed using the unpaired Student's *t* test. The data are shown as the mean ± SEM, n = 5-6. $^*p < 0.05$, $^{**}p < 0.01$. Bregs – B regulatory cells, MASLD – metabolic dysfunction-associated steatotic liver disease, TLS – tertiary lymphoid structures.

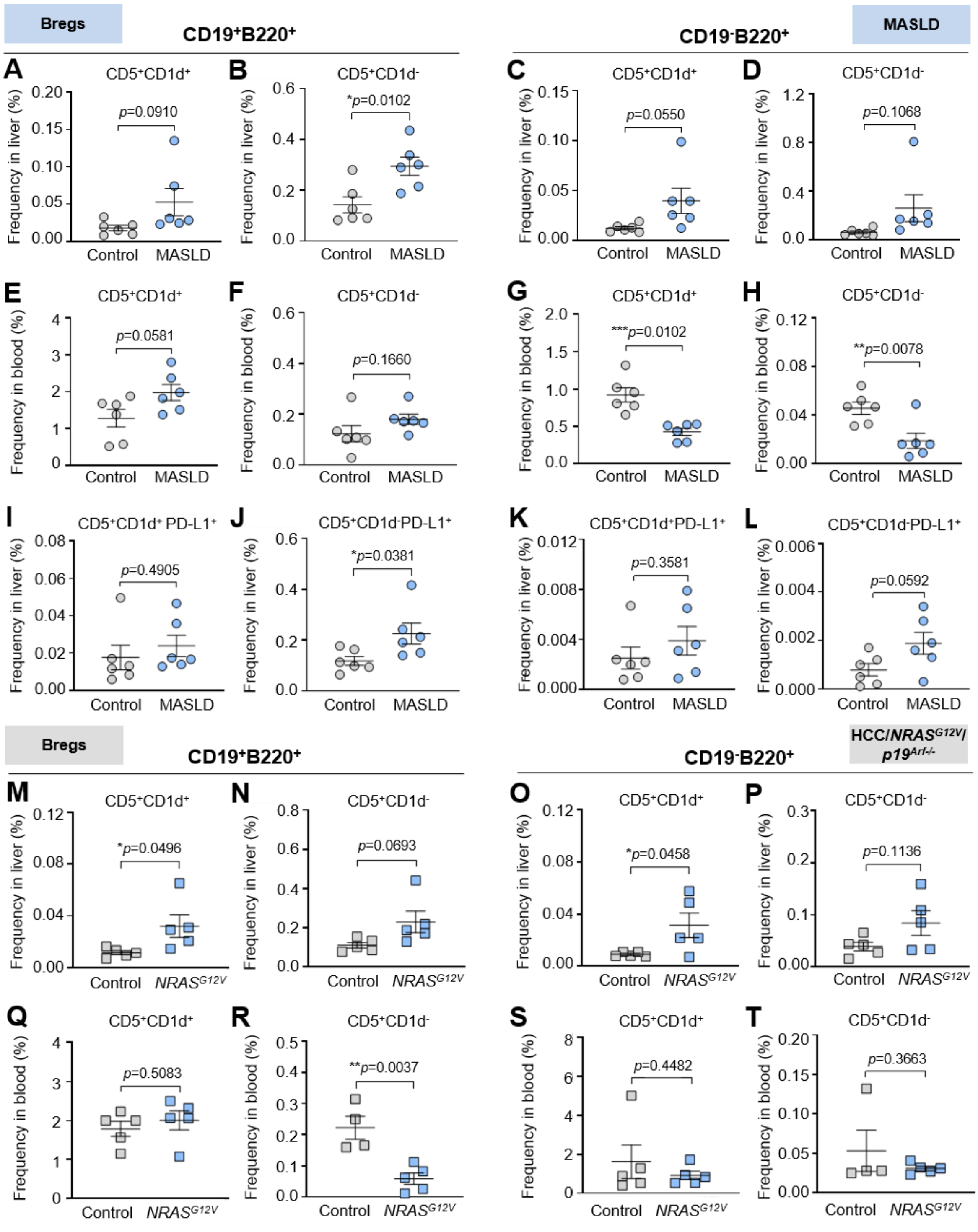

Fig. S2. Bregs phenotype in MASLD and HCC/*NRAS*^{G12V}/*p19*$^{Arf-/-}$ mouse models. (A, B) Frequencies of **(A)** CD19$^+$B220$^+$CD5$^+$CD1d$^+$ and **(B)** CD19$^+$B220$^+$CD5$^+$CD1d$^-$ B cells in the liver of MASLD mice. **(C, D)** Frequencies of **(C)** CD19$^-$B220$^+$CD5$^+$CD1d$^+$ and **(D)** CD19$^-$B220$^+$CD5$^+$CD1d$^-$ B cells in the liver of MASLD mice. **(E, H)** Frequencies of **(E)** CD19$^+$B220$^+$CD5$^+$CD1d$^+$ and **(F)** CD19$^+$B220$^+$CD5$^+$CD1d$^-$ B cells in the blood of MASLD mice. **(G, H)** Frequencies of **(G)** CD19$^-$B220$^+$CD5$^+$CD1d$^+$ and **(H)** CD19$^-$B220$^+$CD5$^+$CD1d$^-$ B cells in the blood of MASLD mice. **(I-L)** Frequencies of PD-L1$^+$-expressing Bregs in the livers

of MASLD mice. **(M, N)** Frequencies of **(M)** $CD19^+B220^+CD5^+CD1d^+$ and **(N)** $CD19^+B220^+CD5^+CD1d^-$ B cells in the liver of HCC/*NRAS*^{G12V}/*p19*$^{Arf-/-}$ mice. **(O, P)** Frequencies of **(O)** $CD19^-B220^+CD5^+CD1d^+$ and **(P)** $CD19^-B220^+CD5^+CD1d^-$ B cells in the liver of HCC/*NRAS*^{G12V}/*p19*$^{Arf-/-}$ mice. **(Q, R)** Frequencies of **(Q)** $CD19^+B220^+CD5^+CD1d^+$ and **(R)** $CD19^+B220^+CD5^+CD1d^-$ B cells in the blood of HCC/*NRAS*^{G12V}/*p19*$^{Arf-/-}$ mice. **(S, T)** Frequencies of **(S)** $CD19^-B220^+CD5^+CD1d^+$ and **(T)** $CD19^-B220^+CD5^+CD1d^-$ B cells in the blood of HCC/*NRAS*^{G12V}/*p19*$^{Arf-/-}$ mice. The data were analyzed using the unpaired Student's *t* test. The data are shown as the mean ± SEM, n = 5-6. $*p < 0.05$, $**p < 0.01$, $***p < 0.001$. Bregs – B regulatory cells, MASLD – metabolic dysfunction-associated steatotic liver disease.

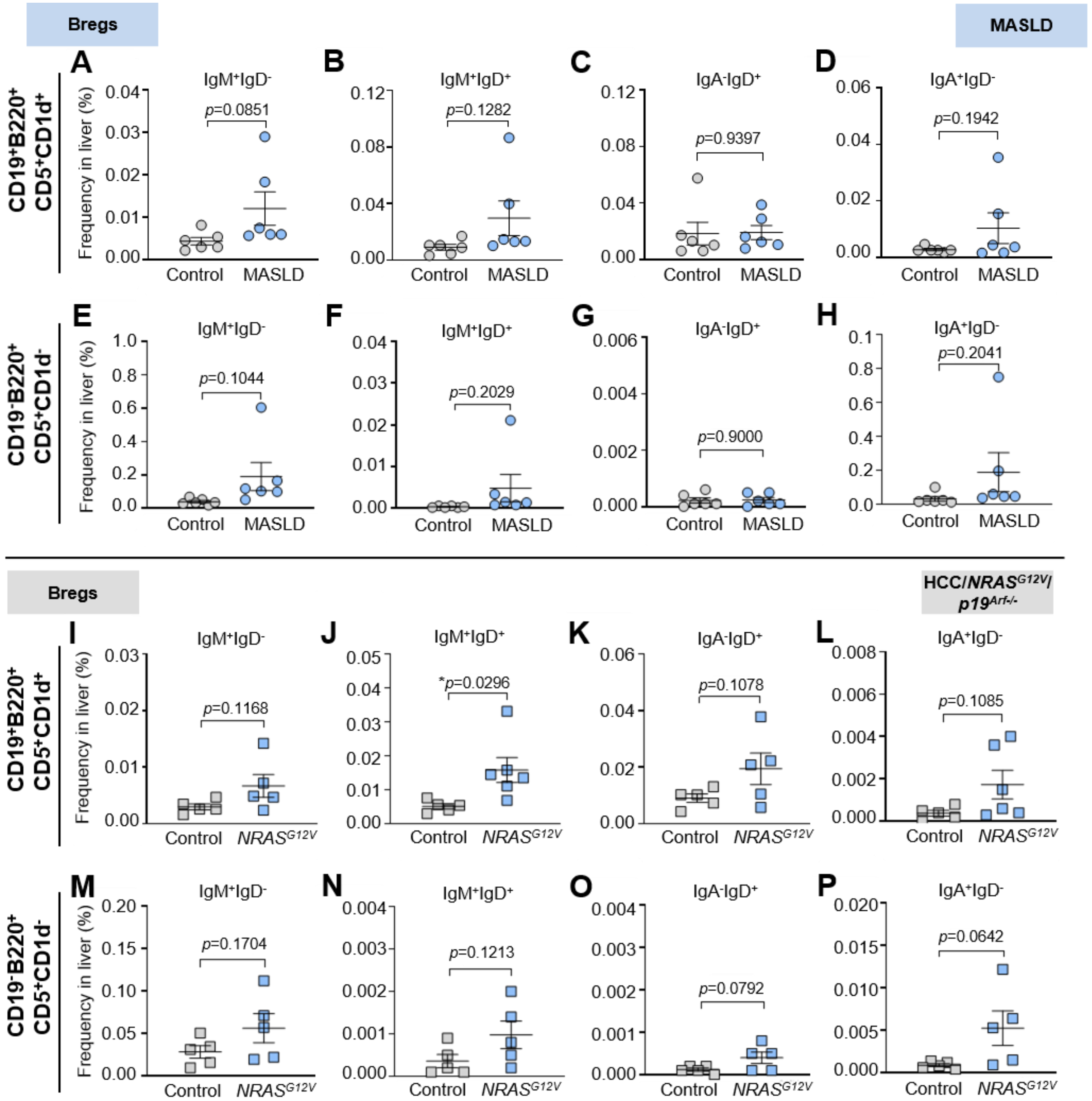

Fig. S3. IgM$^+$, IgD$^+$, and IgA$^+$ Bregs in the livers of MASLD and HCC/*NRAS*^{G12V}/*p19*$^{Arf-/-}$ mice. (A-H), Frequencies of **(A, E)**, IgM$^+$IgD$^-$-, **(B, F)** IgM$^+$IgD$^+$-, **(C, G)** IgA$^-$IgD$^+$-, and **(D, H)** IgA$^+$IgD$^-$-expressing CD19$^+$B220$^+$CD5$^+$CD1d$^+$ and CD19$^-$B220$^+$CD5$^+$CD1d$^-$ Bregs, respectively, in the livers of MASLD mice. **(I-P)** Frequencies of **(I, M)** IgM$^+$IgD$^-$-, **(J, N)** IgM$^+$IgD$^+$-, **(K, O)** IgA$^-$IgD$^+$-, and **(L, P)** IgA$^+$IgD$^-$-expressing CD19$^+$B220$^+$CD5$^+$CD1d$^+$ and CD19$^-$B220$^+$CD5$^+$CD1d$^-$ Bregs, respectively, in the livers of HCC/*NRAS*^{G12V}/*p19*$^{Arf-/-}$ mice. The data were analyzed using the unpaired Student's *t* test. The data are shown as the mean ± SEM, n = 5-6. $^*p < 0.05$. Bregs – B regulatory cells, MASLD – metabolic dysfunction-associated steatotic liver disease.

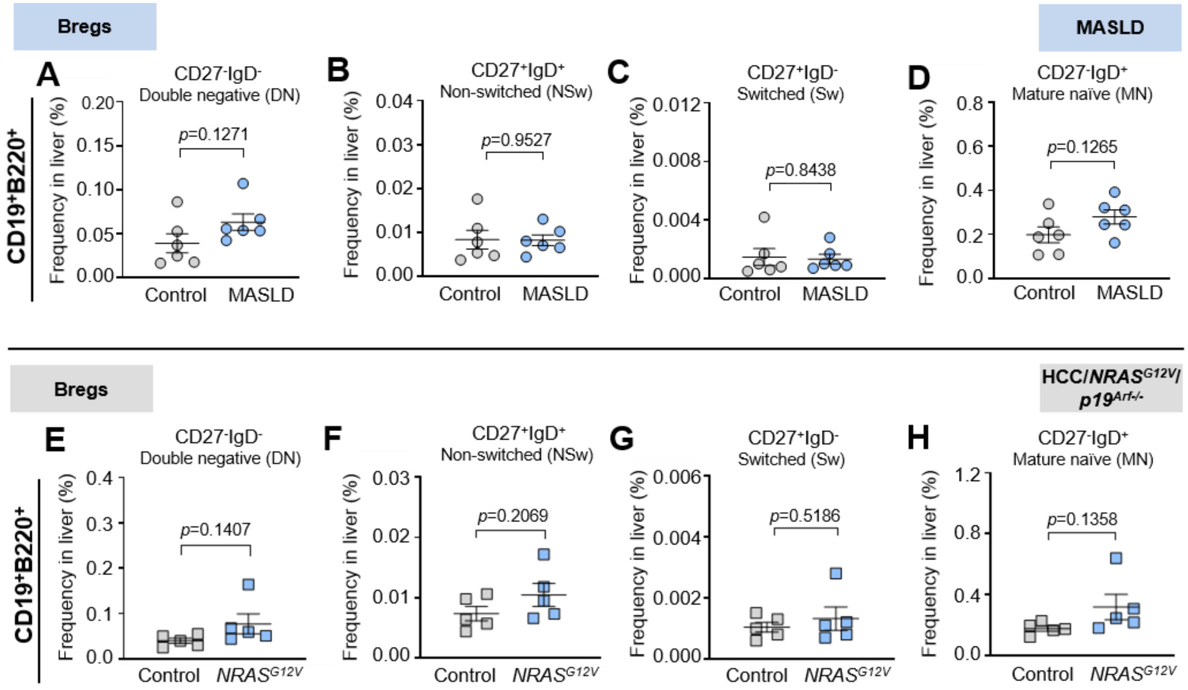

Fig. S4. MBCs in the livers of MASLD and HCC/*NRAS*^{G12V}/*p19*$^{Arf-/-}$ mice. (A-D) Frequencies of **(A)** CD27^{-}IgD^{-} DN, **(B)** CD27^{+}IgD^{+} NSw, **(C)** CD27^{+}IgD^{-} Sw, and **(D)** CD27^{-}IgD^{+} MN MBCs in the livers and blood of MASLD mice. **(E-H)** Frequencies of **(E)** CD27^{-}IgD^{-} DN, **(F)** CD27^{+}IgD^{+} NSw, **(G)** CD27^{+}IgD^{-} Sw, and **(H)** CD27^{-}IgD^{+} MN MBCs in the livers of HCC/*NRAS*^{G12V}/*p19*$^{Arf-/-}$ mice. The data were analyzed using the unpaired Student's *t* test. The data are shown as the mean ± SEM, n = 5-6. MASLD – metabolic dysfunction-associated steatotic liver disease, MBCs – memory B cells, DN – double negative, NSw – non-switched, Sw – switched, MN – mature naïve.

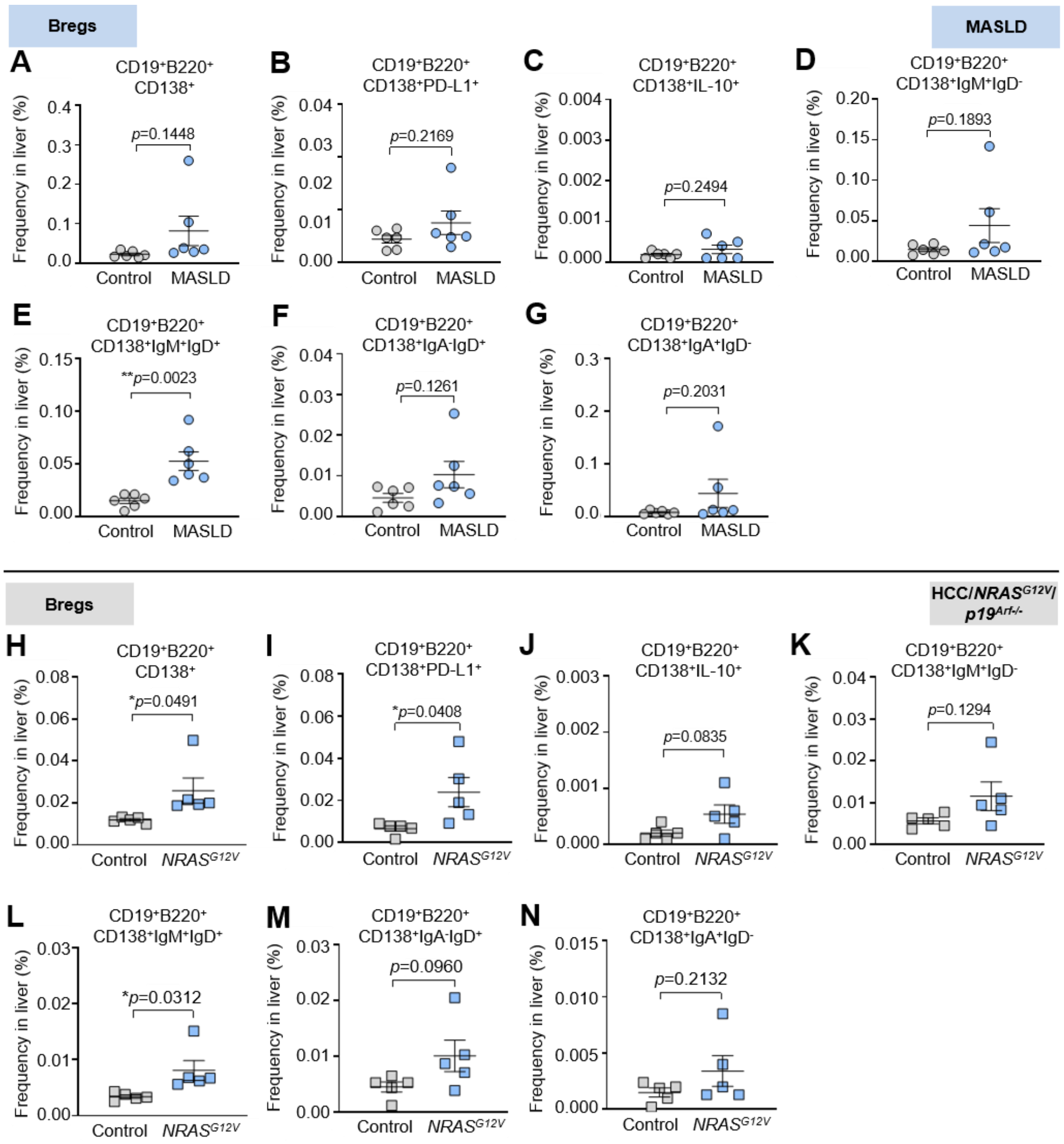

Fig. S5. Elevated frequencies of PD-L1$^+$- and IgM$^+$IgD$^+$-expressing CD19$^+$B220$^+$CD138$^+$ PBs in the livers of MASLD and HCC/*NRAS*^{G12V}/*p19*$^{Arf-/-}$ mice. (A-C) Frequencies of **(A)** CD19$^+$B220$^+$CD138$^+$, **(B)** CD19$^+$B220$^+$CD138$^+$PD-L1$^+$, and **(C)** CD19$^+$B220$^+$CD138$^+$IL-10$^+$ B cells in the livers of MASLD mice. **(D-G)** Frequencies of **(D)** IgM$^+$IgD$^-$, **(E)** IgM$^+$IgD$^+$, **(F)**, IgA$^-$IgD$^+$, and **(G)** IgA$^+$IgD$^-$ CD19$^+$B220$^+$CD138$^+$ B cells in the livers of MASLD mice. **(H-F)** Frequencies of **(H)** CD19$^+$B220$^+$CD138$^+$, **(I)** CD19$^+$B220$^+$CD138$^+$PD-L1$^+$, and **(J)** CD19$^+$B220$^+$CD138$^+$IL-10$^+$ B cells in the livers of HCC/*NRAS*^{G12V}/*p19*$^{Arf-/-}$ mice. **(K-N)** Frequencies of **(K)** IgM$^+$IgD$^-$, **(L)** IgM$^+$IgD$^+$, **(M)**, IgA$^-$IgD$^+$, and **(N)** IgA$^+$IgD$^-$ CD19$^+$B220$^+$CD138$^+$ B cells in the livers of HCC/*NRAS*^{G12V}/*p19*$^{Arf-/-}$ mice. The data were analyzed using the unpaired Student's *t* test. The data are shown as the mean ± SEM, n = 5-6. $^*p$ < 0.05, $^{**}p$ < 0.01. Bregs – B regulatory cells, MASLD – metabolic dysfunction-associated steatotic liver disease, PBs –plasmablasts.

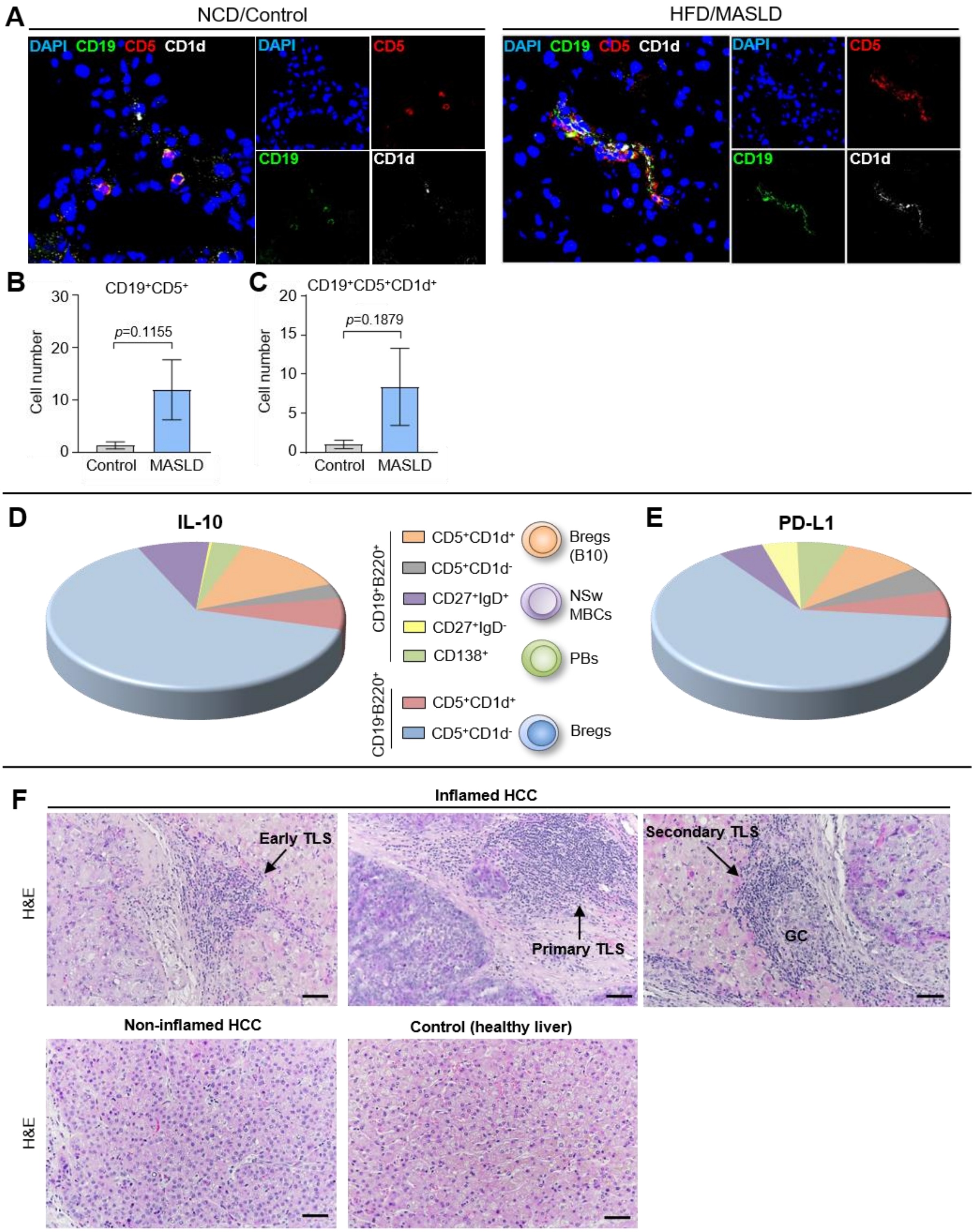

Fig. S6. An increase in the numbers of $CD19^+CD5^+$, $CD19^+CD5^+CD1d^+$ Breg cells in murine MASLD; comparison of B cell subsets based on IL-10 and PD-L1 expression in murine HCC/*CaMIN*; TLS structures in murine HCC. (A) Representative IF images on frozen liver sections were obtained from MASLD mice stained with CD19 (green), CD5 (red), and CD1d (white) antibodies and counterstained with DAPI (blue). **(B-C)** Quantification of **(B)** $CD19^+CD5^+$, and **(C)** $CD19^+CD5^+CD1d^+$ cells in the livers obtained from MASLD mice. (**D-E)**

Pie charts demonstrating the proportions of **(D)** IL-10^{+}, and **(E)** PD-L1^{+} B cell populations in the livers of mice with HCC/*CaMIN*. **(F)** TLS detection in H&E-stained human HCC, demonstrating early, primary, and secondary TLS types and the presence of a germinal center in the inflamed HCC tissue. Controls: non-inflamed HCC (on the left) and healthy liver tissue (on the right). Magnification 400x; scale bar, 100 µm. The data were analyzed using the unpaired Student's *t* test. The data are shown as the mean ± SEM, n = 5-6. NCD – normal chow diet, HFD – high-fat diet, NSw MBC – non-switched memory B cell, Breg – B regulatory cell, PB – plasmablast, TLS – tertiary lymphoid structures, GC – germinal center, MASLD – metabolic dysfunction-associated steatotic liver disease.

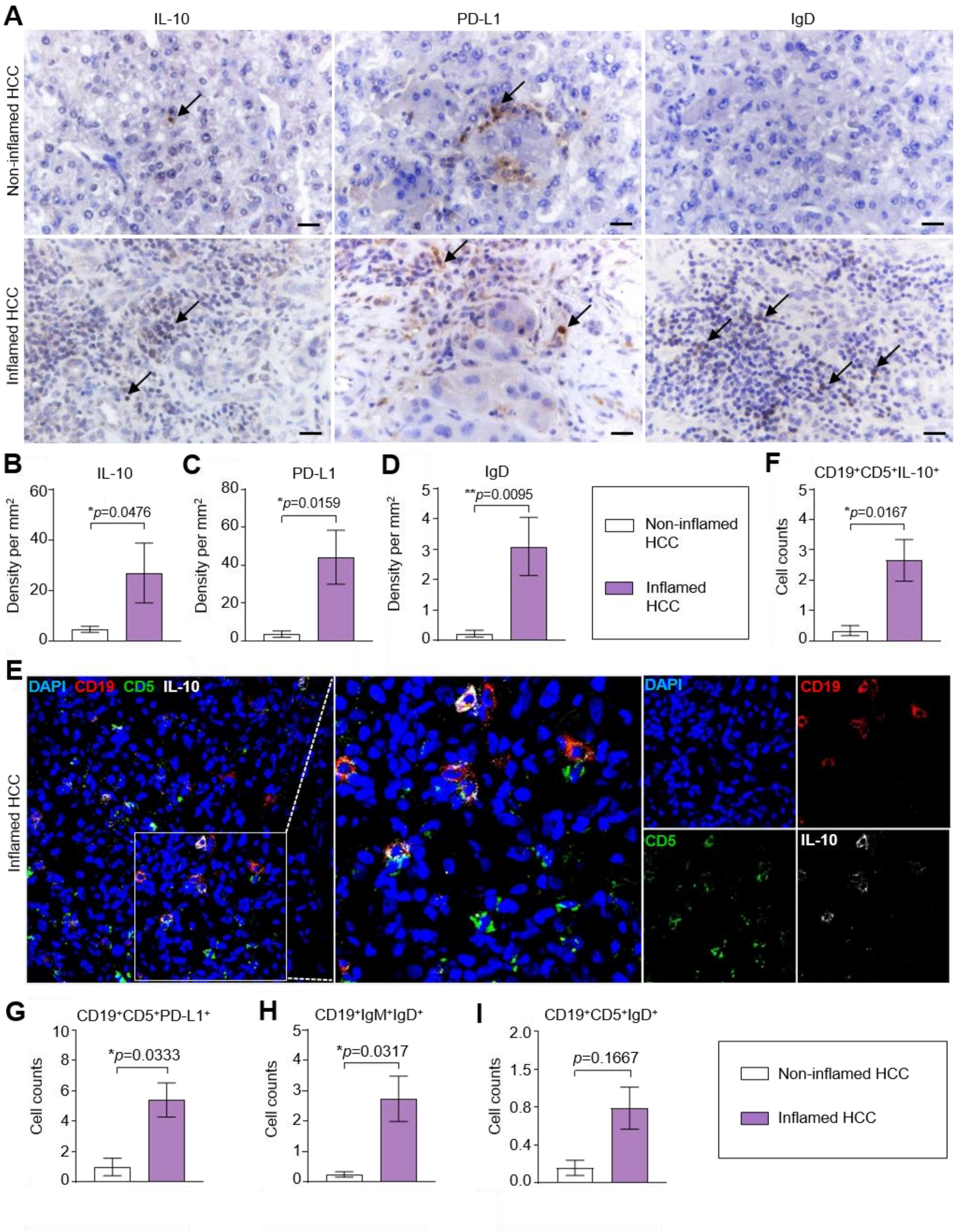

Fig. S7. The inflamed subtype of human HCC is characterized by the increased numbers of CD19⁺CD5⁺IL-10⁺, CD19⁺CD5⁺PD-L1⁺, and CD19⁺IgM⁺IgD⁺ B cells. (A) Representative images of IHC of IL-10, PD-L1, and IgD expression in the non-inflamed and inflamed human HCC tissues. Scale bar, 100 µm. **(B-D)** Density of cellular markers **(B)** IL-10, **(C)** PD-L1, and **(D)** IgD in the non-inflamed and inflamed human HCC tissues. The data were analyzed using the Mann–Whitney nonparametric test, n=10. $*p < 0.05$, $**p < 0.01$. **(E)** Representative IF images on frozen liver sections from patients with inflamed HCC stained

with CD19 (red), CD5 (green), and IL-10 (white) antibodies and counterstained with DAPI (blue). **(F-I)** Quantification of **(G)** $CD19^{+}CD5^{+}IL\text{-}10^{+}$, **(G)** $CD19^{+}CD5^{+}PD\text{-}L1^{+}$, **(H)** $CD19^{+}IgM^{+}IgD^{+}$, and **(I)** $CD19^{+}CD5^{+}IgD^{+}$ cells in the non-inflamed and inflamed human HCC tissues. The data were analyzed using the Mann–Whitney nonparametric test, n=7. *$p < 0.05$.

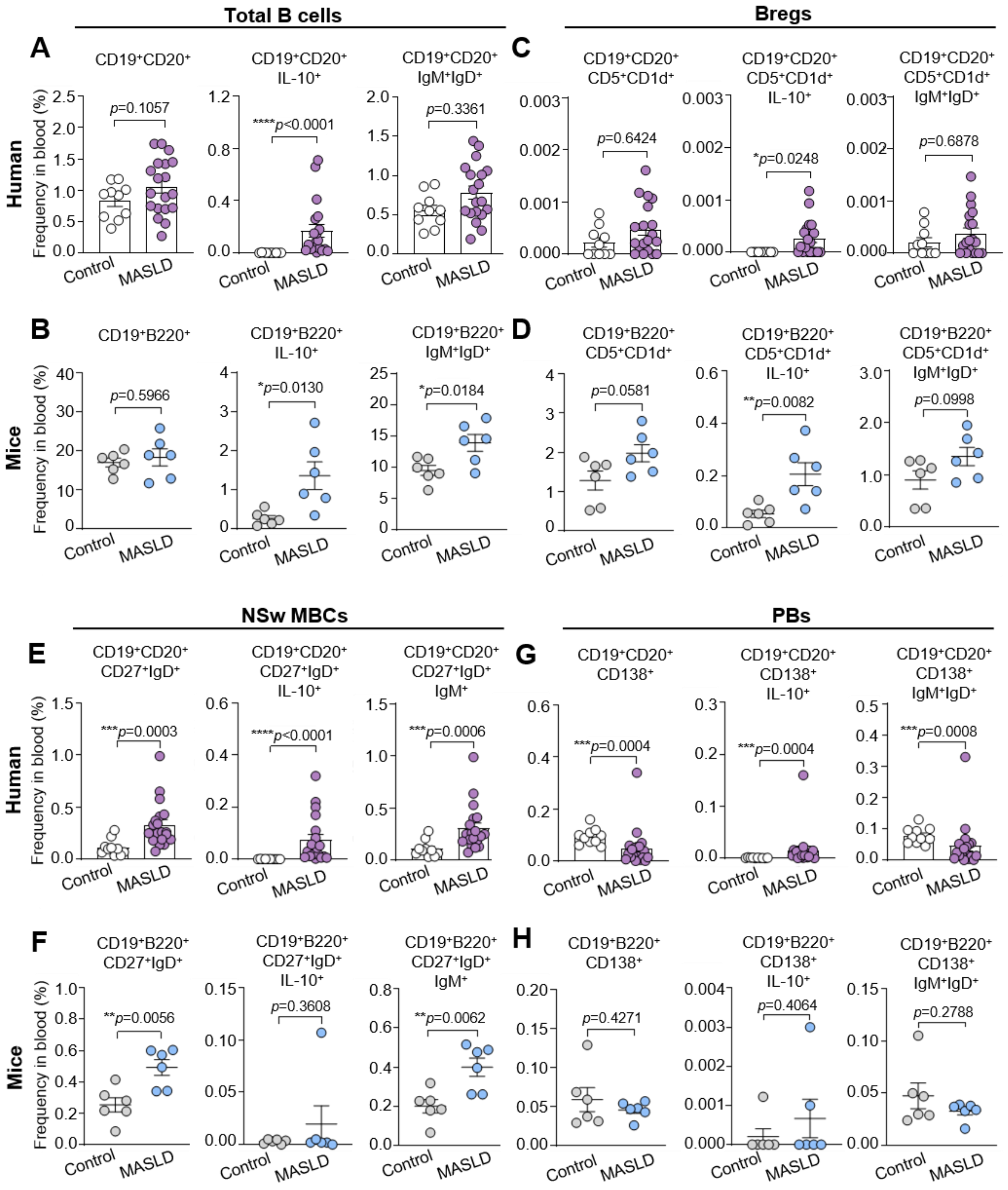

Fig. S8. B cell phenotyping in the blood of patients with MASLD revealed a strong increase of IL-10 on total CD19$^+$CD20$^+$ B cells, on CD19$^+$CD20$^+$CD5$^+$CD1d$^+$ Bregs and on CD19$^+$CD20$^+$CD27$^+$IgD$^+$ NS MBCs. The data strongly correlated between mice and humans with MASLD. (A, B) Frequencies of total B cells in the blood of **(A)** patients and **(B)** mice with MASLD. **(C, D)** Frequencies of Bregs in the blood of **(C)** patients and **(D)** mice with MASLD. **(E, F)** Frequencies of NSw MBCs in the blood of **(E)** patients and **(F)** mice with MASLD. **(G, H)** Frequencies of PBs in the blood of **(G)** patients and **(H)** mice with MASLD. The data were analyzed using the Mann–Whitney nonparametric test, n=4. $*p < 0.05$, $**p <$

0.01, ***$p < 0.001$, ****$p < 0.0001$. Bregs – B regulatory cells, NSw MBCs – non-switched memory B cells, PBs – plasmablasts. MASLD – metabolic dysfunction-associated steatotic liver disease.

Graphical abstract. Essential roles of $IgM^{+}IgD^{+}$ Bregs, NSw MBCs and PBs in the progression of MASLD and HCC.

Murine models were used to clarify the phenotypic characteristics and investigate the role of B lymphocytes in precancerous (MASLD) and cancerous (HCC) liver diseases. The results obtained in mice have also been verified in patients with MASLD and HCC. Blood and liver tissues obtained from mice and patients were analyzed using multicolor FACS, ELISA, IHC and IF. Our study revealed an increase in numbers of several specific B cell subsets: 1) $CD19^{+}B220^{+}CD5^{+}CD1d^{+}$ B10 Bregs; 2) $CD19^{-}B220^{+}CD5^{+}CD1d^{-}$ Bregs; 3) $CD19^{+}B220^{+}CD5^{-}CD27^{+}$ NSw MBCs; and 4) $CD19^{+}B220^{+}CD138^{+}$ PBs, all of which highly expressed IgM, IgD receptors, and the inhibitory molecules PD-L1 and IL-10. Two pie chart diagrams, demonstrating the proportions of IL-10^{+}- and PD-$L1^{+}$-expressing B cell subsets in the livers of HCC/*CaMIN* mice and depicting the most immunosuppressive B cell subset, $CD19^{-}B220^{+}CD5^{+}CD1d^{-}$ Bregs, in murine HCC. All four identified B cell subsets might play a protumorigenic role in precancerous (MASLD) and cancerous (HCC) liver disease progression.

Supplementary tables

Table S1. Clinicopathological characteristics of the human cohort with HCC (n=16).

**Age (years)**	
Median (range)	69.08 (50-82)
**Gender, n (%)**	
Male	11 (68.75%)
Female	5 (31.25%)
**BMI**	
<25	5 (31.25%)
≥25	9 (56.25%)
Unknown	2 (12.50%)
**AFP, n (%)**	
<20 ng/ml	8 (50.00%)
≥20 ng/ml	3 (18.75%)
Unknown	5 (31.25%)
**GGT, n (%)**	
<54 U/L	7 (43.75%)
8 (50.00%)	8 (50.00%)
Unknown	1 (6.25%)
**Microvascular invasion, n (%)**	
Yes	3 (18.75%)
No	13 (81.25%)
**Inflammatory type**	
Inflamed	8 (50.00%)
Non-inflamed	8 (50.00%)
**Tumor stage, TNM, n (%)**	
1	6 (37.50%)
2	4 (25.00%)
3	1 (6.25%)
4	1 (6.25%)
Unknown	4 (25.00%)
**Tumor grade, n (%)**	

I	0
II	12 (75.00%)
III	1 (6.25%)
Unknown	3 (18.75%)

BMI – body mass index; AFP – alpha-1-fetoprotein; GGT – gamma-glutamyltransferase; TNM – tumor, nodes, metastasis.

Table S2. Clinical characteristics of the human cohort with MASLD (n=19).

**Age (years)**	
Median (range)	48 (32-74)
**Gender, n (%)**	
Male	14 (73.68%)
Female	5 (26.32%)
**BMI**	
<25	18 (94.73%)
≥25	1 (5.26%)
**AFP, n (%)**	
<20 ng/ml	0 (0%)
≥20 ng/ml	19 (100%)
**GGT, n (%)**	
<54 U/L	11 (57.89%)
≥54 U/L	8 (42.10%)

BMI – body mass index; AFP – alpha-1-fetoprotein; GGT – gamma-glutamyltransferase.

Table S3. Key resources table.

Reagent or resource	Source	Identifier
**Antibodies used for flow cytometry**		
Anti-mouse B220	BioLegend	Cat # 103236;<br>RRID: AB_893354
Anti-mouse CD5	BioLegend	Cat # 100622;<br>RRID: AB_2562773
Anti-mouse CD1d	BioLegend	Cat # 123520;<br>RRID: AB_2715918
Anti-mouse PD-L1	BioLegend	Cat # 124343;<br>RRID: AB_2894674
Anti-mouse IgM	BioLegend	Cat # 406504;<br>RRID: AB_315054
Anti-mouse IgD	BioLegend	Cat # 405704;<br>RRID: AB_315026
Anti-mouse CD27	BioLegend	Cat # 124233;<br>RRID: AB_2687192
Anti-mouse CD138	BioLegend	Cat # 142515;<br>RRID: AB_2562336
Anti-mouse CD267	BioLegend	Cat # 133404;<br>RRID: AB_2240584
Anti-mouse IL-10	BioLegend	Cat # 505022;<br>RRID: AB_2563240
Anti-mouse CD19	BD Bioscience	Cat # 563157;<br>RRID: AB_2738035
Anti-mouse IgA	eBioscience	Cat # 13599482;<br>RRID: AB_466863
Anti-mouse CD20	BioLegend	Cat # 152104;<br>RRID: AB_2629619
Anti-human CD3	BioLegend	Cat # 344846;<br>RRID: AB_2800923
Anti-human CD11c	BioLegend	Cat # 301626;<br>RRID: AB_10662381
Anti-human CD19	BioLegend	Cat # 302270;<br>RRID: AB_2832581
Anti-human CD20	BioLegend	Cat # 302332;<br>RRID: AB_2563805
Anti-human CD5	BioLegend	Cat # 364020;<br>RRID: AB_2565941
Anti-human CD1d	BioLegend	Cat # 350316;<br>RRID: AB_2687379
Anti-human CD38	BioLegend	Cat # 303550;<br>RRID: AB_2860784
Anti-human CD27	BioLegend	Cat # 356428;<br>RRID: AB_261671
Anti-human IgM	BioLegend	Cat # 314544;

		RRID: AB_2800832
Anti-human PD-L1	BioLegend	Cat # 329718;<br>RRID: AB_2561687
Anti-human IL-10	BioLegend	Cat # 501426;<br>RRID: AB_2566744
Anti-human CD45	BD Biosciences	Cat # 563792;<br>RRID: AB_2869519
Anti-human CD24	BD Biosciences	Cat # 741831;<br>RRID: AB_2871166
Anti-human IgD	BD Biosciences	Cat # 566138;<br>RRID: AB_2739536
TruStain FcX™ (anti-mouse CD16/32) antibody	BioLegend	Cat # 101320;<br>RRID: AB_1574975
Human TruStain FcX™ (Fc Receptor Blocking Solution)	BioLegend	Cat # 422302;<br>RRID: AB_2818986
BV785 Streptavidin	BioLegend	Cat # 405249; RRID: N/A
APC-Cy7 Streptavidin	BioLegend	Cat # 405208; RRID: N/A
**Antibodies used for IHC and IF**		
Anti-CD19	Invitrogen	Cat # PA5-27442;<br>RRID: AB_2544918
Anti-CD19	eBioscience™	Cat # 14019482;<br>RRID: AB_2637171
Anti-CD19	eBioscience™	Cat # 53-0194-82;<br>RRID: AB_2637167
Anti-CD5	Epredia™Lab Vision™	Cat # MS-393-R7;<br>RRID: AB_61248
Anti-CD5	Invitrogen	Cat # MA5-17781;<br>RRID: AB_2539165
Anti-CD5	Santa Cruz Animal Health	Cat # sc-1180;<br>RRID: AB_627112
Anti-CD1d	antibodies-online.com	Cat # ABIN3022497;<br>RRID: N/A
Anti-IL-10	Invitrogen	Cat # PA5-85660;<br>RRID: AB_2792799
Anti-PD-L1	Invitrogen	Cat # PA5-28115;<br>RRID: AB_2545591
Anti-IgD	Abcam	Cat # AB124795;<br>RRID: AB_10974228
Anti-IgM	Santa Cruz Animal Health	Cat # sc-53347;<br>RRID: AB_672096
Donkey anti-rat IgG,<br>Alexa Fluor 647	Invitrogen	Cat # A-78947;<br>RRID: AB_2910635
Donkey anti-rabbit IgG,<br>Alexa Fluor 555	Invitrogen	Cat # A-31572;<br>RRID: AB_162543
Donkey anti-mouse IgG,<br>Alexa Fluor 488	Invitrogen	Cat # A-21202;<br>RRID: AB_141607

Goat anti-mouse IgG1, Alexa Fluor 488	Invitrogen	Cat # A-21121; RRID: AB_2535764
Goat anti-rat IgG, Alexa Fluor 568	Invitrogen	Cat # A-11077; RRID: AB_2534121
Goat anti-rabbit IgG, Alexa Fluor 647	Invitrogen	Cat # A-21245; RRID: AB_2535813
**Antibodies used for B-cell depletion**		
Anti-mouse CD20	BioLegend	Cat # 152116; RRID: AB_2629619
**Chemicals, peptides and recombinant proteins**		
Complete DMEM (cDMEM) medium	Gibco	Cat # 31966021
Complete RPMI 1640 (cRPMI) medium	Gibco	Cat # 72400047
Fetal bovine serum (FBS)	Serena	Cat # S-FBSP-EU-015
Penicillin/Streptomycin	Gibco	Cat # 15070063
Collagenase D	Roche	Cat # 11088882001
DNAse I	Sigma Aldrich	Cat # D4527
EDTA	Carl Roth	Cat # 8043.2
Heparin 5000	Ratiopharm	Cat # PZN-03029820
Ammonium chloride (NH_4Cl)	Carl Roth	Cat # P726.2
Potassium hydrogen carbonate ($KHCO_3$)	Carl Roth	Cat # X887.2
Phosphate buffered saline (PBS)	Gibco	Cat # 70013-016
Ficoll-Paque PLUS	GE Healthcare	Cat # GE17-1440-02
Trypan blue	Sigma Aldrich	Cat # T815
Alexa Fluor™ 350 NHS Ester (Succinimidyl Ester)	Life Technologies (Molecular Probe)	Cat # 11579036
Argentum	Honeywell-Fluka	Cat # 31630-2506
Eosin	Merck	Cat # 1.115935-0100
Hematoxylin	Sigma Aldrich	Cat # 517-28-2
Sirius red (Direct Red 80)	Sigma Aldrich	Cat # 2610-10-8
Oil red O	Sigma Aldrich	Cat # O-0625
DAPI (4’,6-diamidino-2-phenylindole)	Sigma-Aldrich	Cat # D9542-50MG
Normal goat serum	Abcam	Cat # ab7481
Bovine serum albumin fraction V	Carl Roth	Cat # 8076.4
Triton X-100	Sigma Aldrich	Cat # 9002-93-1

Sodium citrate dihydrate	Fisher Scientific	Cat # 11945071
**Commercial kits / assays**		
QIAGEN EndoFree Maxi Kit	Qiagen	Cat # 12362
Mouse Immunoglobulin D (IgD) ELISA Kit	CusaBio	Cat # CSB-E15761m
Epredia™ UltraVision™ Quanto Detection System HRP DAB	Fisher Scientific	Cat # 12673997
**Experimental models: animals / murine strains**		
C57BL/6J	Charles River	Strain code: 632; RRID: IMSR_JAX:000664
B6.129X1-Cdkn2a^{tm1Cjs} (*p19*$^{Arf-/-}$)	Jackson Laboratory	Strain code: 029676; RRID: IMSR_JAX:029676
B6.129S2-Igh-6$^{tm1Cgn/J}$ (µMT)	Jackson Laboratory	Strain Code: 002288; RRID: IMSR_JAX:002288
B6.129P2-Igh-J$^{tm1Cgn/J}$ (JHT)	Jackson Laboratory	Strain Code: 002438; RRID: IMSR_JAX:002438
**Software and algorithms**		
FlowJo, v10.8.1	Tree Star Inc.	RRID: SCR_008520
GraphPad Prism, v8.3	GraphPad Software	RRID: SCR_002798
ZEN Digital Imaging for Light Microscopy, 2011	Carl Zeiss AG	RRID: SCR_013672
Fiji/ImageJ	Fiji	RRID: SCR_002285
Adobe Photoshop CS5	Adobe	RRID: SCR_014199
**Other**		
Instrument: Cytek Aurora	Cytek	
Instrument: LSR II	BD Biosciences	
Instrument: LUNA-FL™ Automated Fluorescence Cell Counter	Logos Biosystems	
Instrument: Microtome HM 335 E	Microm	
Instrument: Cryotome HM 500 OM	Microm	
Instrument: Zeiss LSM 700 confocal laser-scanning microscope	Carl Zeiss AG	
Instrument: BX51, Olympus	Olympus	
Nikon Eclipse Ti2 Inverted Microscope	Nikon	

<u>**Supplementary references**</u>

Author names in bold designate shared co-first authorship.

[1] Kamijo T, Zindy F, Roussel MF, et al. Tumor suppression at the mouse INK4a locus mediated by the alternative reading frame product p19ARF. Cell 1997;91:649-659.

[2] Dauch D, Rudalska R, Cossa G, et al. A MYC-aurora kinase A protein complex represents an actionable drug target in p53-altered liver cancer. Nature medicine 2016;22:744-753.

[3] **Kang TW, Yevsa T,** Woller N, et al. Senescence surveillance of pre-malignant hepatocytes limits liver cancer development. Nature 2011;479:547-551.

[4] Kitamura D, Roes J, Kuhn R, at al. B cell-deficient mouse by targeted disruption of the membrane exon of the immunoglobulin mu chain gene. Nature 1991;350:423-426.

[5] Gu H, Zou YR, Rajewsky K. Independent control of immunoglobulin switch recombination at individual switch regions evidenced through Cre-loxP-mediated gene targeting. Cell 1993;73:1155-1164.

[6] Ohashi T, Nakade Y, Ibusuki M, et al. Conophylline inhibits high fat diet-induced non-alcoholic fatty liver disease in mice. PloS one 2019;14:e0210068.

[7] **Bartolome F, Antequera D, de la Cueva M,** et al. Endothelial-specific deficiency of megalin in the brain protects mice against high-fat diet challenge. Journal of neuroinflammation 2020;17:22.

[8] **Petriv N, Neubert L,** Vatashchuk M, et al. Increase of alpha-dicarbonyls in liver and receptor for advanced glycation end products on immune cells are linked to nonalcoholic fatty liver disease and liver cancer. Oncoimmunology 2021;10:1874159.

[9] Shalapour S, Lin XJ, Bastian IN, et al. Inflammation-induced IgA+ cells dismantle anti-liver cancer immunity. Nature 2017;551:340-345.

[10] DiLillo DJ, Hamaguchi Y, Ueda Y, et al. Maintenance of long-lived plasma cells and serological memory despite mature and memory B cell depletion during CD20 immunotherapy in mice. J Immunol 2008;180:361-371.

[11] **Hochnadel I, Hoenicke L,** Petriv N, et al. Safety and efficacy of prophylactic and therapeutic vaccine based on live-attenuated Listeria monocytogenes in hepatobiliary cancers. Oncogene 2022.

[12] **Rudalska R, Dauch D,** Longerich T, et al. In vivo RNAi screening identifies a mechanism of sorafenib resistance in liver cancer. Nature medicine 2014;20:1138-1146.

[13] Eggert T, Wolter K, Ji J, et al. Distinct Functions of Senescence-Associated Immune Responses in Liver Tumor Surveillance and Tumor Progression. Cancer cell 2016;30:533-547.

[14] Neubert L, Borchert P, Stark H, et al. Molecular Profiling of Vascular Remodeling in Chronic Pulmonary Disease. The American journal of pathology 2020;190:1382-1396.
[15] Neubert L, Borchert P, Shin HO, et al. Comprehensive three-dimensional morphology of neoangiogenesis in pulmonary veno-occlusive disease and pulmonary capillary hemangiomatosis. The journal of pathology Clinical research 2019;5:108-114.
[16] Mehlem A, Hagberg CE, Muhl L, at al. Imaging of neutral lipids by oil red O for analyzing the metabolic status in health and disease. Nature protocols 2013;8:1149-1154.
[17] Sia D, Jiao Y, Martinez-Quetglas I, et al. Identification of an Immune-specific Class of Hepatocellular Carcinoma, Based on Molecular Features. Gastroenterology 2017;153:812-826.
[18] **Montironi C, Castet F, Haber PK,** et al. Inflamed and non-inflamed classes of HCC: a revised immunogenomic classification. Gut 2023;72:129-140.
