# Supplementary material for: Essential roles of B cell subsets in the progression of MASLD and HCC
Source: JHEP Rep. 2024 Aug 22;6(12):101189. doi: 10.1016/j.jhepr.2024.101189 (PMC11602976; doi:10.1016/j.jhepr.2024.101189)
Supplement: Multimedia component 2 [file mmc2.docx]

**JHEP Reports**

- 1. **Antibodies**

| **Name** | **Citation** | **Supplier** | **Cat no.** | **Clone no.** |
| --- | --- | --- | --- | --- |
| Anti-mouse B220 | Lang J, et al. 2020. *Nat Communication* | BioLegend | 103236 | RA3-6B2 |
| Anti-mouse CD5 | Fan L, et al. 2020. *J Immunol Res.* | BioLegend | 100622 | 53-7.3 |
| Anti-mouse CD1d | Brutkiewicz RR, et al. 2007. Eur. J. Immunol. | BioLegend | 123520 | 1B1 |
| Anti-mouse PD-L1 | Cripps JG, et al. 2010. Hepatology | BioLegend | 124343 | 10F.9G2 |
| Anti-mouse IgM | Lai M, et al. 2016. *Nat Communication* | BioLegend | 406504 | RMM-1 |
| Anti-mouse IgD | Xu Y, et al. 2022. *Nat Communication* | BioLegend | 405704 | 11-26c.2a |
| Anti-mouse CD27 | White CA, et al.   2014. J Immunol. | BioLegend | 124233 | LG.3A10 |
| Anti-mouse CD138 | Harsha Krovi S, et al. 2020. *Nat Communication* | BioLegend | 142515 | 281-2 |
| Anti-mouse CD267 | Chappaz S, et al. 2021. Cell Reports. | BioLegend | 133404 | 8F10 |
| Anti-mouse IL-10 | Hayashi K, et al. 2020. *Nat Communication* | BioLegend | 505022 | JES5-16E3 |
| Anti-mouse CD19 | Gommerman JL, *et al.* 2000*. J Immunol.* | BD Bioscience | 563157 | 1D3 |
| Anti-mouse IgA | Guo G, *et al.* 2021. *Nat Communication* | eBioscience | 13599482 | 11-44-2 |
| Anti-mouse CD20 | Tang KH, *et al.* 2022. *Cancer Discov*ery | BioLegend | 152104 | SA271G2 |
| Anti-human CD3 | Rutjens E, *et al.* 2007. *J. Immunol*. | BioLegend | 344846 | SK7 |
| Anti-human CD11c | Huizinga R, *et al.* 2013. J Immunol. | BioLegend | 301626 | 3.9 |
| Anti-human CD19 | Szabo PA, et al. 2021. Immunity | BioLegend | 302270 | [HIB19](https://www.biolegend.com/en-us/search-results?Clone=HIB19) |
| Anti-human CD20 | Gagne M, et al. 2022. Cell | BioLegend | 302332 | 2H7 |
| Anti-human CD5 | Fraussen J, *et al.* 2019. J Immunol. | BioLegend | 364020 | L17F12 |
| Anti-human CD1d | Durante-Mangoni E, *et al.* 2004. *J. Immunol.* | BioLegend | 350316 | 51.1 |
| Anti-human CD38 | Lozano-Rodríguez R, *et al.* 2022. *Cell Reports* | BioLegend | 303550 | HIT2 |
| Anti-human CD27 | Gupta R, et al. 2022. *Front Immunology* | BioLegend | 356428 | M-T271 |
| Anti-human IgM | Japp AS, et al. 2021. *Cell* | BioLegend | 314544 | MHM-88 |
| Anti-human PD-L1 | Cañadas I, et al. 2018. *Nat Med* | BioLegend | 329718 | 29E.2A3 |
| Anti-human IL-10 | Saggau C, et al. 2022. *Immunity* | BioLegend | 501426 | JES3-9D7 |
| Anti-human CD45 | Hermiston ML, et al. 2003. *Annu Rev Immunology* | BD Biosciences | 563792 | HI30 |
| Anti-human CD24 | Chtanova T, et al. 2004. *J Immunology* | BD Biosciences | 741831 | ML5 |
| Anti-human IgD | Wei C, et al. 2007. *J Immunology* | BD Biosciences | 566138 | IA6-2 |
| TruStain FcX™ (anti-mouse CD16/32) | Kimura S, et al. 2020. *Nat Communication* | BioLegend | 101320 | 93 |
| Anti-CD19 |  | Invitrogen | PA5-27442 | polyclonal |
| Anti-CD19 | Asano Y, *et al.* 2021. *Nat Communication* | eBioscience™ | 14019482 | 6OMP31 |
| Anti-CD19 | Cho H, *et al.* 2022. *Nat Communication* | eBioscience™ | 53-0194-82 | 6OMP31 |
| Anti-CD5 |  | Epredia™Lab Vision™ | MS-393-R7 | 4C7 |
| Anti-CD5 | Leech JM, *et al.* 2017. *J Immunology* | Invitrogen | MA5-17781 | 53-7.3 |
| Anti-CD5 | Gary-Gouy H, *et al.* 2007. *J Immunology* | Santa Cruz Animal Health | sc-1180 | UCH-T2 |
| Anti-CD1d |  | antibodies-online.com | ABIN3022497 | polyclonal |
| Anti-IL-10 | Cavalcante RS, *et al.* 2021. Br *J Pharmacol* | Invitrogen | PA5-85660 | polyclonal |
| Anti-PD-L1 | Johnson DB, *et al.* 2019. *Nat Med* | Invitrogen | PA5-28115 | polyclonal |
| Anti-IgD | Moysi E, *et al.* 2018. *J Clin Invest* | Abcam | AB124795 | EPR6146 |
| Anti-IgM | Dong, S. *et al.* 2023. *Virol Sin* | Santa Cruz Animal Health | sc-53347 | R1/69 |
| Donkey anti-rat IgG, Alexa Fluor 647 |  | Invitrogen | A-78947 | polyclonal |
| Donkey anti-rabbit IgG, Alexa Fluor 555 | - [Jin](https://www.researchgate.net/scientific-contributions/Hengwei-Jin-2167162162?_sg%5B0%5D=5kU0RNYx9kGIUgDdNMQoNQlD9HCDuA0aZmOWmIYrs1EfyFrcAdSv78XiFxjdQOouAmthPdg.FewqwGIyhPuyXgiLs12kAtc6VMZY-nx3h4Cl11dyt20aeAWlf9A3Fpc6mAv_yZHvMWyCE6GGrzIAwN7XjK_8dQ&_sg%5B1%5D=ZYHgs_1lqUdII13SSpJ9t5K6-RWTsZ3wHKNrKUxtETWV9DRpEuBLVuc4fnBgVcbp27rKhHw.Ynb1Uc7uaXwHFqsHnokaRW6KzWmY_2rLHUaQcyOF0DDwYWcvCn7j096SvwpQNF88jYnlo9o_qy95nhURbjY_zg&_tp=eyJjb250ZXh0Ijp7ImZpcnN0UGFnZSI6Il9kaXJlY3QiLCJwYWdlIjoicHVibGljYXRpb24iLCJzZWN0aW9uIjoicGFnZUhlYWRlciJ9fQ) H, *et al.* 2021. *Nat Communication* | Invitrogen | A-31572 | polyclonal |
| Donkey anti-mouse IgG, Alexa Fluor 488 | [Smith](https://bmcbiol.biomedcentral.com/articles/10.1186/s12915-022-01267-6#auth-Andrew_J_-Smith-Aff1-Aff2) AJ, *et al.* 2022. *BMC Biology* | Invitrogen | A-21202 | polyclonal |
| Goat anti-mouse IgG1, Alexa Fluor 488 | [Somasundaram](https://www.nature.com/articles/s41467-017-00452-4/#auth-Rajasekharan-Somasundaram-Aff1) R, *et al.* 2017. *Nat Communication* | Invitrogen | A-21121 | polyclonal |
| Goat anti-rat IgG, Alexa Fluor 568 | Puga I, *et al. 2012. Nat Immunology* | Invitrogen | A-11077 | polyclonal |
| Goat anti-rabbit IgG, Alexa Fluor 647 | Fuentes LA, *et al. 2023.* J Cell Biol. | Invitrogen | A-21245 | polyclonal |
| Anti-mouse CD20 | Tang KH, et al. 2022. *Cancer Discov.* | BioLegend | 152116 | SA271G2 |

- 1. **Organisms**

| **Name** | **Citation** | **Supplier** | **Strain** | **Sex** | **Age** |
| --- | --- | --- | --- | --- | --- |
| Mouse |  | Charles River | *C57BL/6J* | Males and females | 4-8 weeks at HDI |
| Mouse | Kamijo T, *et al. 1997. Cell* | Jackson Laboratory | *B6.129X1-Cdkn2a^tm1Cjs^* (*p19^Arf-/-^*) | Males and females | 4-8 weeks at HDI |
| Mouse | Kitamura D, *et al. 1991. Nature* | Jackson Laboratory | [B6.129S2-Igh-6^tm1Cgn/J^ (µMT)](https://mhh.lavan-os.de/animalRoom/index#strain/4166709) | Males and females | 4-8 weeks at HDI |
| Mouse | Gu H, *et al. 1993. Cell* | Jackson Laboratory | [B6.129P2-Igh-J^tm1Cgn/J^ (JHT](https://mhh.lavan-os.de/animalRoom/index#strain/11874491)) | Males and females | 4-8 weeks at HDI |

- 1. **Biological samples**

| **Description** | **Source** | **Identifier** |
| --- | --- | --- |
| Murine blood (mice with MASLD and HCC) | (see Table 1.2) |  |
| Murine liver tissues (mice with MASLD and HCC) | (see Table 1.2) |  |
| Human blood samples (PBMCs obtained from healthy controls (n =10) and from patients with MASLD (n = 19)) | Hannover Medical School (MHH), 2020-2022 | Ethics Committee of the MHH (approval numbers: 3261_BO_K_2016, 7825_BO_K_2018) |
| Human HCC tumor tissues  (n = 16) | MHH, 2020-2022 | Ethics Committee of the MHH (approval number: 8742_BO_K_2019) |

- 1. **Software**

| **Software name** | **Manufacturer** | **Version** |
| --- | --- | --- |
| FlowJo | Treestar Inc. | 10.8.1 |
| GraphPad Prism, v8.3 | Graphpad Software, Inc. | 8.3 |
| ZEN Digital Imaging for Light Microscopy, 2011 | Carl Zeiss AG | 2.1 |
| Fiji/ImageJ | Fiji | 1.54e 4 |
| Adobe Photoshop CS5 | Adobe | 12.1 |

- 1. **Other (e.g. drugs, proteins, vectors etc.)**

| **Reagent or Resource** | **Supplier/Reference** | **Identifier** |
| --- | --- | --- |
| SB13 vector | Yant SR, *et al.* 2004. *Mol Cell Bio* |  |
| CaN vector | Kang TW, Yevsa T, *et al.* 2011. *Nature* |  |
| CaMIN vector | Kang TW, Yevsa T, *et al.* 2011. *Nature* |  |
| Human TruStain FcX™ (Fc Receptor Blocking Solution) | BioLegend | 422302 |
| BV785 Streptavidin | BioLegend | 405249 |
| APC-Cy7 Streptavidin | BioLegend | 405208 |
| Complete DMEM (cDMEM) medium | Gibco | 31966021 |
| Complete RPMI 1640 (cRPMI) medium | Gibco | 72400047 |
| Fetal bovine serum (FBS) | Serena | S-FBSP-EU-015 |
| Penicillin/Streptomycin | Gibco | 15070063 |
| Collagenase D | Roche | 11088882001 |
| DNAse I | Sigma Aldrich | D4527 |
| Ethylenediaminetetraacetic acid (EDTA) | Carl Roth | 8043.2 |
| Heparin 5000 | Ratiopharm | PZN-03029820 |
| Ammonium chloride (NH_4_Cl) | Carl Roth | P726.2 |
| Potassium hydrogen carbonate (KHCO_3_) | Carl Roth | X887.2 |
| Phosphate buffered saline (PBS) | Gibco | 70013-016 |
| Ficoll-Paque PLUS | GE Healthcare | GE17-1440-02 |
| Trypan blue | Sigma Aldrich | T815 |
| Alexa Fluor™ 350 NHS Ester (Succinimidyl Ester) | Life Technologies (Molecular Probe) | 11579036 |
| Argentum | Honeywell-Fluka | 31630-2506 |
| Eosin | Merck | 1.115935-0100 |
| Hematoxylin | Sigma Aldrich | 517-28-2 |
| Sirius red (Direct Red 80) | Sigma Aldrich | 2610-10-8 |
| Oil red O | Sigma Aldrich | O-0625 |
| DAPI (4’,6-diamidino-2-phenylindole) | Sigma-Aldrich | D9542-50MG |
| Normal goat serum | Abcam | ab7481 |
| Bovine serum albumin fraction V | Carl Roth | 8076.4 |
| Triton X-100 | Sigma Aldrich | [9002-93-1](https://www.sigmaaldrich.com/DE/de/search/9002-93-1?focus=products&page=1&perPage=30&sort=relevance&term=9002-93-1&type=cas_number) |
| Sodium citrate dihydrate | Fisher Scientific | 11945071 |
| QIAGEN EndoFree Maxi Kit | Qiagen | 12362 |
| Mouse Immunoglobulin D (IgD) ELISA Kit | CusaBio | CSB-E15761m |
| Epredia™ UltraVision™ Quanto Detection System HRP DAB | Fisher Scientific | 12673997 |

- 1. **Details of the corresponding methods author for the manuscript:**

| Tetyana Yevsa, PhD  Department of Gastroenterology, Hepatology, Infectious Diseases and Endocrinology  Hannover Medical School  Carl-Neuberg Str. 1  30625 Hannover, Germany  Tel.: +49 (0) 511 532 83164  Fax: +49 (0) 511 532 5692  E-mail address: [Yevsa.Tetyana@mh-hannover.de](mailto:Yevsa.Tetyana@mh-hannover.de) |
| --- |
