# Supplementary material for: Essential roles of B cell subsets in the progression of MASLD and HCC
Source: JHEP Rep. 2024 Aug 22;6(12):101189. doi: 10.1016/j.jhepr.2024.101189 (PMC11602976; doi:10.1016/j.jhepr.2024.101189)
Supplement: Multimedia component 4 [file mmc4.pdf]

JHEP|Reports

Essential roles of B cell subsets in the progression of MASLD and HCC

Nataliia Petriv[1], Huizhen Suo[1], Inga Hochnadel[1], Kai Timrott[2], Nina Bondarenko[1,3,4], Lavinia Neubert[4], Elena Reinhard[5], Nils Jedicke[1], Patrick Kaufhold[1], Carlos Alberto Guzmán[5], Ralf Lichtinghagen[6], Michael P. Manns[1], Heike Bantel[1], Tetyana Yevsa[1,*]

JHEP Reports **2024**. vol. 6 | 1–16

Check for updates

Background & Aims: Hepatocellular carcinoma (HCC) is the third leading cause of cancer-related death. Metabolic dysfunction-associated steatotic liver disease (MASLD) is a significant cause of HCC. Current treatment options for HCC are very limited. Recent evidence highlights B cells as key drivers in MASLD progression toward HCC. However, it remains unclear whether multiple B cell populations or a distinct B cell subset regulates inflammatory responses during liver disease progression. The scope of this study was to define protumorigenic B cell subsets in MASLD and HCC.

Methods: Multicolor flow cytometry, immunohistochemistry, and immunofluorescence analyses were performed to investigate B cell populations locally (in liver tissue) and systemically (in the blood) in mice with MASLD (n = 6) and HCC (n = 5–6). The results obtained in mice were also verified in patients with MASLD (n = 19) and HCC (n = 16).

Results: Our study revealed an increase of two regulatory B cell (Breg) subsets, $CD19^+B220^+CD5^+CD1d^+$ (p <0.0001) and $CD19^-B220^+CD5^+CD1d^-$ (p <0.0001), both of which highly overexpress IgM/IgD, PD-L1, and IL-10, in the livers of mice with MASLD and HCC. Furthermore, we showed that B-cell depletion therapy in combination with a *Listeria*-based vaccine decreased $CD19^-B220^+CD5^+CD1d^-$ Bregs (p = 0.0103), and improved survival of mice with HCC. We also found $CD19^+CD5^+IL\text{-}10^+$ (p = 0.0167), $CD19^+CD5^+PD\text{-}L1^+$ (p = 0.0333) and $CD19^+CD5^+IgM^+IgD^+$ (p = 0.0317) B cells in human HCCs. In addition, strong overexpression of IgM/IgD, PD-L1, IL-10, were detected on non-switched memory B cells (p = 0.0049) and plasmablasts (p = 0.0020). The examination of blood samples obtained from patients with MASLD showed an increase of total B cells expressing IL-10 (p <0.0001) and IgM/IgD (p = 0.3361), $CD19^+CD20^+CD5^+CD1d^+$ Bregs (p = 0.6424) and $CD19^+CD20^+CD27^+$ non-switched memory B cells (p = 0.0003).

Conclusions: Our results provide novel insights into the protumorigenic roles of several B cell subsets, the specific targeting of which could abrogate the progression of liver disease.

© 2024 The Authors. Published by Elsevier B.V. on behalf of European Association for the Study of the Liver (EASL). This is an open access article under the CC BY license (http://creativecommons.org/licenses/by/4.0/).

Introduction

Hepatocellular carcinoma (HCC) is the third leading cause of cancer-related mortality.[1,2] Metabolic dysfunction-associated steatotic liver disease (MASLD; formerly known as non-alcoholic fatty liver disease [NAFLD])[3] and its most severe manifestation, metabolic dysfunction-associated steatohepatitis (MASH; formerly known as non-alcoholic steatohepatitis [NASH]),[3] have been described as emerging important causes of HCC.[4]

In addition to MASLD, chronic liver diseases such as viral hepatitis, alcohol abuse and aflatoxin exposure can lead to several gene mutations and the overexpression of different oncogenes in the liver, resulting in HCC development.[2] Current treatment options for HCC are limited to the early stages of the disease and do not protect against recurrence.[2] There is an urgent need for further therapies for prevention and at the early and advanced stages of HCC.

Immunotherapy has recently been acknowledged and approved as the first-line therapy for treating HCC.[5] To date, novel systemic therapies, including immune checkpoint inhibitors (ICIs), tyrosine kinase inhibitors, and monoclonal antibodies, have challenged the use of conventional therapies for HCC.[2] Previous studies have shown that HCC tumors can be stratified into two classes: inflamed ('hot') and non-inflamed ('cold').[6,7] The inflamed subtype of HCC is characterized by a significant immune cell infiltration of T and B cells, natural killer (NK) cells, dendritic cells, and macrophages within the tumor microenvironment[6,7] and is associated with an enhanced response to ICI therapy. Despite the promising effectiveness of ICI treatment observed in HCC patients, a considerable number of patients still face resistance to this therapy.[8]

B cells perform various immunological functions. Several reports have indicated that B cells can mediate antitumor effects.[9,10] Additionally, in our study, B cells were found to limit

* Corresponding author. Address: Department of Gastroenterology, Hepatology, Infectious Diseases and Endocrinology, Hannover Medical School, Carl-Neuberg Str. 1, 30625 Hannover, Germany. Tel.: +49-511-532-83164; Fax: +49-511-532-5692.
E-mail address: Yevsa.Tetyana@mh-hannover.de (T. Yevsa).
https://doi.org/10.1016/j.jhepr.2024.101189

the growth of established diethylnitrosamine-induced liver cancer.[11] Moreover, several recent studies have indicated an important role for B cells in promoting cancer progression.[10,12] In our previous study, we showed that 1 week after early therapeutic vaccination of HCC-bearing animals with *Listeria monocytogenes ΔactA/ΔinlB* expressing ovalbumin (designated LmAIO) significant decreases in B cell populations were detected in the blood of the LmAIO group animals in comparison with the PBS control group.[13] In particular, a highly significant fourfold decrease in $CD19^+$ and $CD19^+B220^{low}$ B cell subpopulations was detected in LmAIO-vaccinated animals protected from HCC development in comparison with those in the PBS control group, indicating the tumor-promoting role of B cells in HCC.[13]

Several B cell types, such as B regulatory cells (Bregs), memory B cells (MBCs), plasma cells (PCs) and plasmablasts (PBs), have been shown to have immunomodulatory effects in infectious diseases and tumors.[9,14–16] In particular, Bregs represent a subset of immunomodulatory B cells that have recently been described in the pathogenesis of many chronic diseases, including cancer.[9,10,16] Bregs are a heterogeneous population of immunosuppressive cells that support immunological tolerance primarily through the release of anti-inflammatory mediators, such as IL-10, and the expression of inhibitory molecules (PD-L1, FasL, and CD1d).[9,14,15] Bregs can originate from a distinct B cell subpopulation.[14,15] Tedder and colleagues classified a unique subset of IL-10-producing $CD19^{hi}CD1d^{hi}CD5^+$ B cells (B10 cells) in mice and humans.[17] Recently, IgA^+-producing B cells have been shown to play a critical role in the progression of MASH-driven HCC in mice and humans.[18] Elevated levels of serum IgA have been reported in patients with MASLD.[18] In addition, IgA^+ B cells in mice and humans have been shown to be involved in the pathogenesis of MASH and to contribute to MASH-induced liver fibrosis.[19]

Little is known about mouse MBCs during cancer progression, and most of the related information comes from human studies. Based on IgD and CD27 expression, MBCs are currently separated into several subpopulations: $CD27^-IgD^-$ double-negative (DN), $CD27^+IgD^+$ non-switched (NSw), $CD27^+IgD^-$ switched (Sw) and $CD27^-IgD^+$ mature naive (MN) cells.[20] In humans, close to 40% of the B cells in the blood are MBCs identified by CD27 and IgD expression,[20] whereas in mice, only a minor MBC population expresses the classic memory cell marker CD27.[21,22] Recently, Weisel *et al.*[21] reported that CD1d, but not CD5, is expressed by murine MBCs. Tang *et al.*[23] observed the enrichment of MBCs within HCC tumor tissue. Other studies have shown that the percentage of MBCs decreases as HCC progresses.[24] Thus, the prognostic impact of MBCs in HCC remains controversial.

Despite many studies on B cells in liver diseases, it remains unclear whether multiple B cell populations or a distinct B cell subset regulates inflammatory responses during the progression of MASLD and HCC. Therefore, in this study, we aimed to perform a detailed analysis, clarify the phenotypic characteristics, and investigate the role of several distinct subtypes of B lymphocytes in precancerous (MASLD) and cancerous (HCC) liver diseases. We used murine models established in our previous studies[25–28] as well as spectral multicolor flow cytometry (FACS) analysis and investigated several B cell populations locally (in the liver) and systemically (in the blood) of mice with MASLD and two types of HCC. The results obtained in mice have also been verified in patients with MASLD and HCC.

Materials and methods

Human samples

Human samples were obtained from 16 patients with HCC and 19 patients with MASLD patients (35 patients in total) who were treated at the Hannover Medical School (MHH) from September 2020 to September 2022 (Tables S1 and S2). Healthy donor adults (n = 10) were included in this study if they reported neither chronic medical conditions nor current medical therapy, obesity, diabetes, etc. Patients with concurrent autoimmune diseases, syphilis, human immunodeficiency virus, alcohol abuse, and pregnant or breastfeeding women were not included in the analysis.

Animal experiments

All animal experiments were performed in compliance with ethical regulations and with the approval of the Lower Saxonian State Office for Consumer Protection and Food Safety (LAVES, Niedersächsisches Landesamt für Verbraucherschutz und Lebensmittelsicherheit; AZ 18/2808, 15/1766).

The detailed Materials and methods section can be found in the Supplementary data.

Results

Precancerous (MASLD) and cancerous (HCC) liver disease models

To induce MASLD, mice were fed a high-fat diet (HFD) *ad libitum* for 14 weeks (Fig. 1A). The control mice were fed a normal chow diet (NCD).[28] Compared with NCD-fed control mice, C57BL/6J mice fed an HFD (MASLD group) exhibited rapid weight gain (Fig. 1B). General weight gain was accompanied by a significant increase in liver weights in the MASLD group (Fig. 1C). Oil Red O staining of liver sections from the MASLD mice revealed a significant increase in the number of neutral lipid droplets (Fig. 1D and E). Further examination of liver sections revealed enhanced lipid accumulation, ballooning, and a liver fibrosis development (enhanced Sirius red) in mice with MASLD compared with control mice (Fig. 1D and E).

To induce HCC, the transposable elements encoding $NRAS^{G12V}$, together with *Sleeping Beauty 13* (*SB13*) transposase, were delivered into the hepatocytes of $p19^{Arf-/-}$ mice (Fig. 1F), as previously described.[25–28] Stable intrahepatic delivery of $NRAS^{G12V}$ to $p19^{Arf-/-}$ mice resulted in advanced HCC/$NRAS^{G12V}/p19^{Arf-/-}$ development within 4 weeks (Fig. 1F and G). The development of multiple singular tumor nodules was confirmed by experienced pathologists using H&E and argentum staining (Fig. 1H).

Because the oncogene *c-Myc* is one of the most abundant oncogenes expressed in HCC,[29] we used this aggressive HCC model as the main HCC model in our study. HCC/*CaMIN* was induced by the intrahepatic codelivery of both *c-Myc* and $NRAS^{G12V}$ transposons into the livers of wild-type (WT) C57BL/6J mice (Fig. 1I and J). This combination resulted in the formation of aggressive multinodular HCC/*CaMIN* within 5 weeks, as confirmed by pathologists using H&E and argentum staining (Fig. 1K).

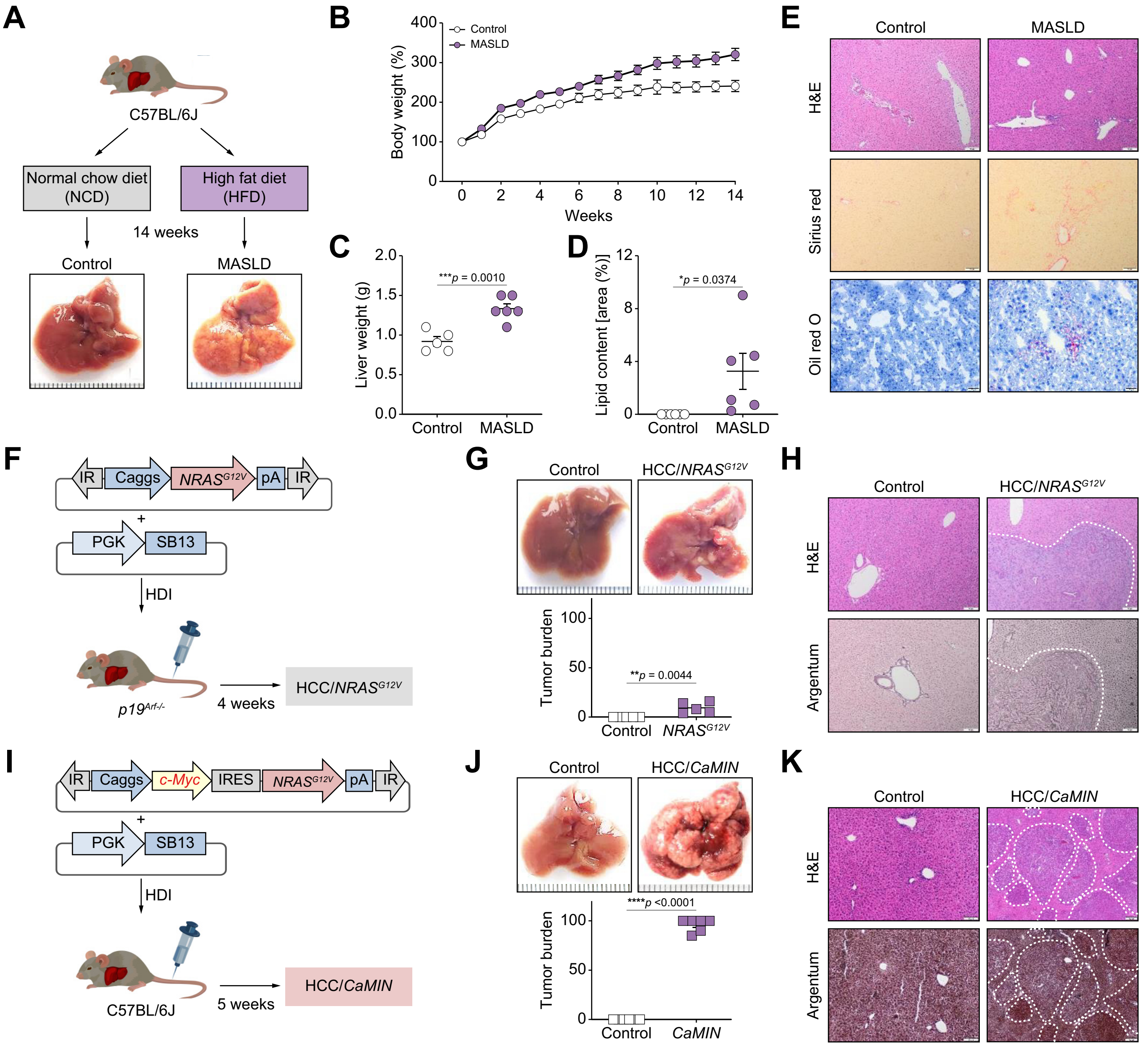

Fig. 1. Experimental models and vectors. Three study models are shown: (A–E) MASLD, (F–H) HCC/*NRAS*^{G12V}/*p19*$^{Arf-/-}$, and (I–K) HCC/*CaMIN*. (A) Schematic outline of the MASLD mouse model. (B) Body weight and (C) liver weight development in the MASLD model. (D) Quantification of lipid content in the MASLD model. (E) Representative liver sections stained with H&E, Sirius red, and Oil red O in the MASLD model. Scale bar, 50 µm. (F) Schematic outline of the HCC/*NRAS*^{G12V}/*p19*$^{Arf-/-}$ mouse model. (G) Representative liver images and the liver tumor burden in the HCC/*NRAS*^{G12V}/*p19*$^{Arf-/-}$ mouse model. (H) Representative liver sections from the HCC/*NRAS*^{G12V}/*p19*$^{Arf-/-}$ model mice stained with H&E and argentum. The dotted line shows the border of a tumor nodule. Scale bar, 50 µm. (I) Schematic outline of the HCC/*CaMIN* mouse model. (J) Representative liver images and the liver tumor burden in the HCC/*CaMIN* mouse model. (K) Representative liver sections from the HCC/*CaMIN* model mice stained with H&E and argentum. Scale bar, 50 µm. The data were analyzed using the unpaired Student's *t* test. The data are shown as the mean ± SEM, n = 5–6. *p <0.05, **p <0.01, ***p <0.001, ****p <0.0001. HCC, hepatocellular carcinoma; HDI, hydrodynamic tail vein injection; HFD, high-fat diet; IR, inverted repeats; IRES, internal ribosome entry site; MASLD, metabolic dysfunction-associated steatotic liver disease; NCD, normal chow diet; pA, polyadenylation site; pCaggs, synthetic CAG promoter; PGK, phosphoglycerate kinase promoter; *SB13 – Sleeping Beauty 13* (transposase).

Strong increase in PD-L1$^+$ and IL-10$^+$-expressing CD19$^+$B220$^+$CD5$^+$CD1d$^+$ and CD19$^-$B220$^+$CD5$^+$CD1d$^-$ Bregs in murine MASLD and HCC *in situ*

We first performed phenotyping of B cell populations in murine livers and blood by multicolor FACS. Using the gating strategy shown in Fig. S1A and based on CD5 and CD1d expression, we identified several Breg subsets in HCC/*CaMIN* (Fig. 2) and in MASLD and HCC/*NRAS*^{G12V}/*p19*$^{Arf-/-}$ (Fig. S2). Our results demonstrated a significant increase in CD5$^+$CD1d$^+$ and CD5$^+$CD1d$^-$ Bregs among CD19$^+$B220$^+$ B cells in the HCC/*CaMIN* model (Fig. 2A, B). The expression of CD1d and CD5 by CD19$^-$B220$^+$ B cells changed only slightly in mice with HCC/*CaMIN* (Fig. 2C). Moreover, the frequencies of CD5$^+$CD1d$^-$-expressing CD19$^-$B220$^+$ B cells significantly increased in animals

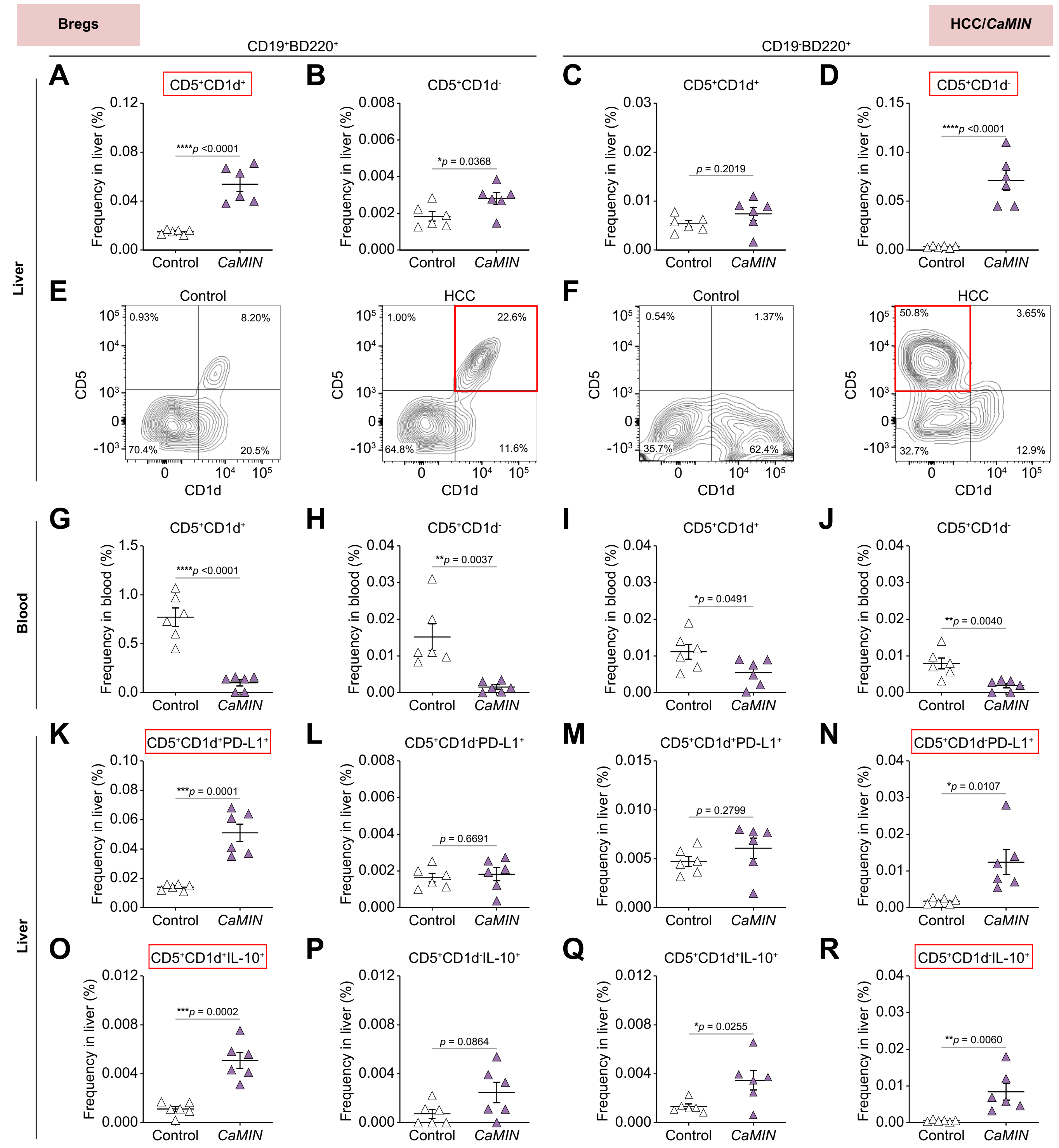

Fig. 2. There was a strong increase in the number of CD19$^+$B220$^+$CD5$^+$CD1d$^+$ and CD19$^-$B220$^+$CD5$^+$CD1d$^-$ Bregs and high PD-L1 and IL-10 expression in murine HCC/*CaMIN*. (A, B) Frequencies of (A) CD19$^+$B220$^+$CD5$^+$CD1d$^+$ and (B) CD19$^+$B220$^+$CD5$^+$CD1d$^-$ B cells in the liver. (C, D) Frequencies of (C) CD19$^-$B220$^+$CD5$^+$CD1d$^+$ and (D) CD19$^-$B220$^+$CD5$^+$CD1d$^-$ B cells in the liver. (E, F) Representative FACS plots of Breg subsets gated on (E) CD19$^+$B220$^+$ and (F) CD19$^-$B220$^+$ B cells in the liver. (G–J) Frequencies of Breg subsets in the blood. (K–N) Frequencies of PD-L1$^+$-expressing (K-L) CD19$^+$B220$^+$ and (M, N) CD19$^-$B220$^+$ Bregs in the liver. (O–R) Frequencies of IL-10$^+$-expressing (O, P) CD19$^+$B220$^+$ and (Q, R) CD19$^-$B220$^+$ Bregs in the liver. The data were analyzed using the unpaired Student's *t* test. The data are shown as the mean ± SEM, n = 6. **p* <0.05, ***p* <0.01, ****p* <0.001, *****p* <0.0001. Fig. S2 shows the MASLD and HCC/*NRAS^{G12V}/p19$^{Arf-/-}$* models. Bregs, B regulatory cells; HCC, hepatocellular carcinoma; PD-L1, programmed death-ligand 1.

with HCC/*CaMIN* (Fig. 2D). Representative FACS plots revealed differences in CD5$^+$CD1d$^+$ and CD5$^+$CD1d$^-$ Breg subtypes among CD19$^+$B220$^+$ and CD19$^-$B220$^+$ B cells in the HCC/*CaMIN* model (Fig. 2E, F, respectively). Importantly, an increase in Bregs was detected mainly locally in HCC liver tissues *in situ* (Fig. 2A–D); however, in the blood of HCC/*CaMIN* mice, all tested Breg populations were strongly decreased (Fig. 2G–J), indicating active B-cell migration. To further confirm the migration of B cells, we evaluated for the expression of CXCR5, a chemokine receptor that plays a crucial role in B-cell homing[30,31] (Fig. S1B–E). Our findings demonstrated a significant increase of CXCR5$^+$-expressing Bregs during HCC/*CAMIN* development (Fig. S1B–E). In particular, we observed a strong increase of CXCR5$^+$CD19$^+$B220$^+$CD5$^+$CD1d$^+$ and CXCR5$^+$CD19$^-$B220$^+$CD5$^+$CD1d$^{+/-}$ Breg populations in murine HCC/*CAMIN* (Fig. S1B–E). In addition, histopathological examination of H&E-stained HCC tissue sections indicated the presence of tertiary lymphoid structures (TLSs) in murine livers with HCC (Fig. S1F).

In the next step, we aimed to characterize the inhibitory status of Breg subsets in the livers of HCC-harboring animals by analyzing PD-L1 and IL-10 expression. We found that Bregs demonstrated elevated numbers of PD-L1 (Fig. 2K–N) and IL-10 (Fig. 2O–R). However, increases of both parameters were detected in the CD19$^+$B220$^+$CD5$^+$CD1d$^+$ (Fig. 2K–O) and CD19$^-$B220$^+$CD5$^+$CD1d$^-$ (Fig. 2N–R) subsets of Bregs.

We next tested the Breg populations in mice with MASLD (Fig. S2A–L) and another HCC model, HCC/*NRAS*^{G12V}/*p19*$^{Arf-/-}$ (Fig. S2M–T). Although not as strongly as in the aggressive HCC/*CaMIN* model, an increase in CD5$^+$CD1d$^+$ and CD5$^+$CD1d$^-$ Bregs in CD19$^+$B220$^+$ B cells was detected in mice with MASLD (Fig. S2A, B) and HCC/*NRAS*^{G12V}/*p19*$^{Arf-/-}$ (Fig. S2M, N). The percentage of CD19$^-$B220$^+$CD5$^+$CD1d$^+$ Bregs tended to increase in mice with MASLD (Fig. S2C) and significantly increased in mice with HCC/*NRAS*^{G12V}/*p19*$^{Arf-/-}$ (Fig. S2O). In addition, an increase in the frequency of CD19$^-$B220$^+$CD5$^+$CD1d$^-$ B cells, although not significant, was detected in the livers of the mice with MASLD (Fig. S2D) and HCC/*NRAS*^{G12}/*p19*$^{Arf-/-}$ (Fig. S2P). The loss of Bregs in the blood of mice with MASLD (Fig. 2SE–H) and HCC/*NRAS*^{G12V}/*p19*$^{Arf-/-}$ (Fig. 2SQ–T) was detected; however, the results were not as consistent as those in mice with HCC/*CaMIN* (Fig. 2G–J). We then detected elevated numbers of PD-L1$^+$-expressing Bregs in the livers of mice with MASLD (Fig. S2I–L).

Overall, we identified two types of Bregs in the liver (CD19$^+$B220$^+$CD5$^+$CD1d$^+$ and CD19$^-$B220$^+$CD5$^+$CD1d$^-$), which exhibit an inhibitory phenotype characterized by high expression of PD-L1 and IL-10 in aggressive murine HCC/*CaMIN*. Notably, an increase in the number of Bregs was predominantly observed within the liver tissues of mice with MASLD and HCC, suggesting a local effect. However, in the blood of HCC/*CaMIN* mice, all tested Breg populations decreased in number. In addition, Bregs upregulated the expression of the homing receptor CXCR5 in HCC/*CaMIN* livers.

Increased IgM$^+$- and IgD$^+$-expressing CD19$^+$B220$^+$CD5$^+$CD1d$^+$ and CD19$^-$B220$^+$CD5$^+$CD1d$^-$ Bregs in the liver and upregulated secretion of IgA in the plasma of mice harboring aggressive HCC

We next examined the expression of IgD, IgM, and IgA in the main types of Bregs (CD19$^+$B220$^+$CD5$^+$CD1d$^+$ and CD19$^-$B220$^+$CD5$^+$CD1d$^-$) in mice with MASLD and HCC using multicolor FACS.

Our study demonstrated a significant increase in IgM$^+$IgD$^-$, IgM$^+$IgD$^+$, and IgA$^-$IgD$^+$-expressing CD19$^+$B220$^+$CD5$^+$CD1d$^+$ Bregs in the livers of mice with HCC/*CaMIN* (Fig. 3A–C). Interestingly, no changes were observed in the frequencies of IgA$^+$IgD$^-$-expressing CD19$^+$B220$^+$CD5$^+$CD1d$^+$ Bregs (Fig. 3D) in HCC/*CaMIN* mice. A significant increase in IgM$^+$IgD$^+$ and IgA$^-$IgD$^+$-expressing CD19$^-$B220$^+$CD5$^+$CD1d$^-$ Bregs was detected in HCC/*CaMIN* mice (Fig. 3E–H). Additionally, similar tendencies were observed in the numbers of IgM$^+$IgD$^-$, IgM$^+$IgD$^+$, and IgA$^-$IgD$^+$ cells among the Bregs (Fig. 3I–L and M–P).

We then tested the expression of IgD, IgM and IgA in the main types of Breg populations in mice with MASLD (Fig. S3A–H) and another HCC mouse model, HCC/*NRAS*^{G12V}/*p19*$^{Arf-/-}$, using multicolor FACS (Fig. S3I–P). We detected a significant increase in the IgM$^+$IgD$^+$-expressing CD19$^+$B220$^+$CD5$^+$CD1d$^+$ Breg population in the HCC/*NRAS*^{G12V}/*p19*$^{Arf-/-}$ group (Fig. S3J). Moreover, increases (not significant) in the frequencies of IgD-, IgM-, and IgA-expressing CD19$^+$B220$^+$ CD5$^+$CD1d$^+$ and CD19$^-$B220$^+$CD5$^+$CD1d$^-$ Bregs were detected in mice with MASLD (Fig. S3A–H) and HCC/*NRAS*^{G12V}/*p19*$^{Arf-/-}$ (Fig. S3I–P).

In addition, we performed an ELISA to examine the secreted levels of immunoglobulins (IgM, IgG, IgA, and IgD) in plasma samples obtained from mice with MASLD and HCC/*CaMIN* (Fig. 3Q–T). Only a moderate (not significant) increase in the IgM level was detected in MASLD mice, compared with controls, whereas a significant decrease in the IgM level was detected in the plasma of mice with HCC/*CaMIN* (Fig. 3Q). Additionally, compared with control mice maintained on a standard diet, mice with MASLD exhibited decreased plasma levels of IgG and IgA (Fig. 3R, S). Similarly, compared with tumor-free control mice, HCC/*CaMIN* mice exhibited decreased plasma IgG levels (Fig. 3R). In contrast to that in the MASLD group, a strong increase in IgA levels was detected in the plasma of mice with HCC/*CaMIN* (Fig. 3S). We did not observe any significant changes in the level of secreted IgD in the plasma of mice with MASLD or HCC/*CaMIN* (Fig. 3T).

These findings indicate the presence of IgD and IgM on the surface of CD19$^+$B220$^+$CD5$^+$CD1d$^+$ and CD19$^-$B220$^+$ CD5$^+$CD1d$^-$ Bregs locally in the livers of mice with aggressive HCC/*CaMIN* development. Secreted IgA was detected in the plasma of HCC/*CaMIN* mice but not in that of mice with MASLD.

Strong increase in IgM$^+$-, PD-L1$^+$- and IL-10$^+$-expressing CD27$^+$IgD$^+$ NSw MBCs in murine HCC livers

We next investigated MBCs in the livers and blood of mice with premalignant liver disease (MASLD) and malignant liver disease (HCC). Using the gating strategy shown in Fig. 4A, we first gated live CD19$^+$B220$^+$ B cells, subsequently excluding CD5$^+$ B cells (a marker of Bregs, as previously reported).[9,15,21] Thereafter, based on the expression of CD27 and IgD, we identified several murine MBC populations: CD27$^-$IgD$^-$ DN, CD27$^+$IgD$^+$ NSw**,** CD27$^+$IgD$^-$ Sw, and CD27$^-$IgD$^+$ MN.

Our results demonstrated a significant decrease in the numbers of CD27$^-$IgD$^-$ DN MBCs in the livers of HCC/*CaMIN* mice compared with control mice (Fig. 4B). A strong increase in

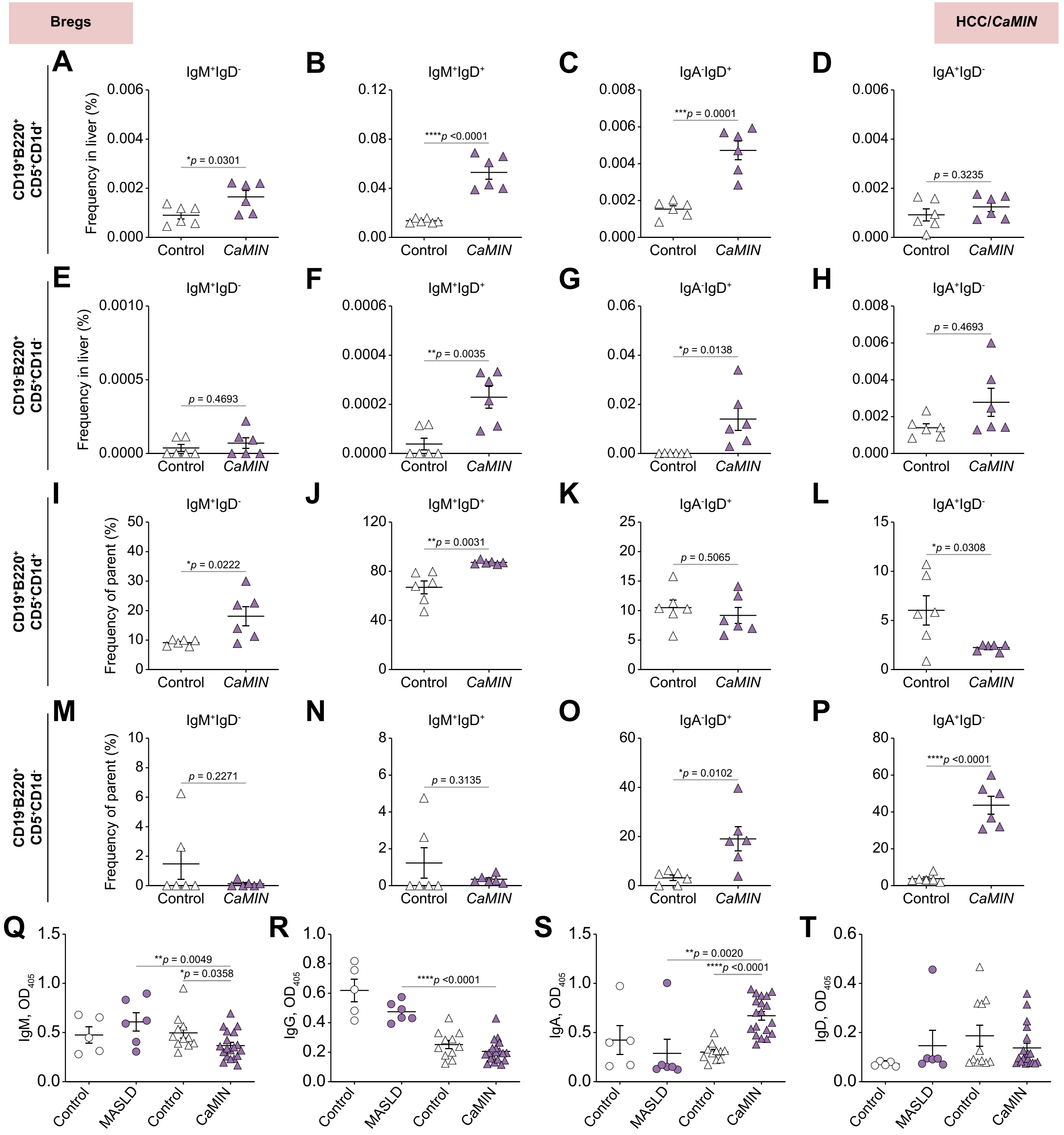

Fig. 3. Upregulation of IgM$^+$- and IgD$^+$-expressing CD19$^+$B220$^+$CD5$^+$CD1d$^+$ and CD19$^-$B220$^+$CD5$^+$CD1d$^-$ Bregs in the livers of HCC/*CaMIN* mice. (A–D) Frequencies of (A) IgM$^+$IgD$^-$-, (B) IgM$^+$IgD$^+$-, (C), IgA$^-$IgD$^+$-, and (D) IgA$^+$IgD$^-$-expressing CD19$^+$B220$^+$CD5$^+$CD1d$^+$ Bregs. (E–H) Frequencies of (E) IgM$^+$IgD$^-$-, (F) IgM$^+$IgD$^+$-, (G) IgA$^-$IgD$^+$-, and (H) IgA$^+$IgD$^-$-expressing CD19$^-$B220$^+$CD5$^+$CD1d$^-$ Bregs. (I–L) Percentage of (I) IgM$^+$IgD$^-$, (J) IgM$^+$IgD$^+$, (K), IgA$^-$IgD$^+$, and (L) IgA$^+$IgD$^-$ among CD19$^+$B220$^+$CD5$^+$CD1d$^+$ Bregs. (M–P) Percentage of (M) IgM$^+$IgD$^-$, (N) IgM$^+$IgD$^+$, (O), IgA$^-$IgD$^+$, and (P) IgA$^+$IgD$^-$ among CD19$^-$B220$^+$CD5$^+$CD1d$^-$ Bregs. (Q–T) ELISA to determine the levels of (Q) IgM, (R) IgG, (S) IgA and (T) IgD in the plasma samples of mice with MASLD and HCC/*CaMIN*. The data were analyzed using the unpaired Student's *t* test. The data are shown as the mean ± SEM, n = 6. **p* <0.05, ***p* <0.01, ****p* <0.001, *****p* <0.0001. Fig. S3 shows the MASLD and HCC/*NRAS^{G12V}*/*p19$^{Arf-/-}$* models. Bregs, B regulatory cells; HCC, hepatocellular carcinoma; MASLD, metabolic dysfunction-associated steatotic liver disease.

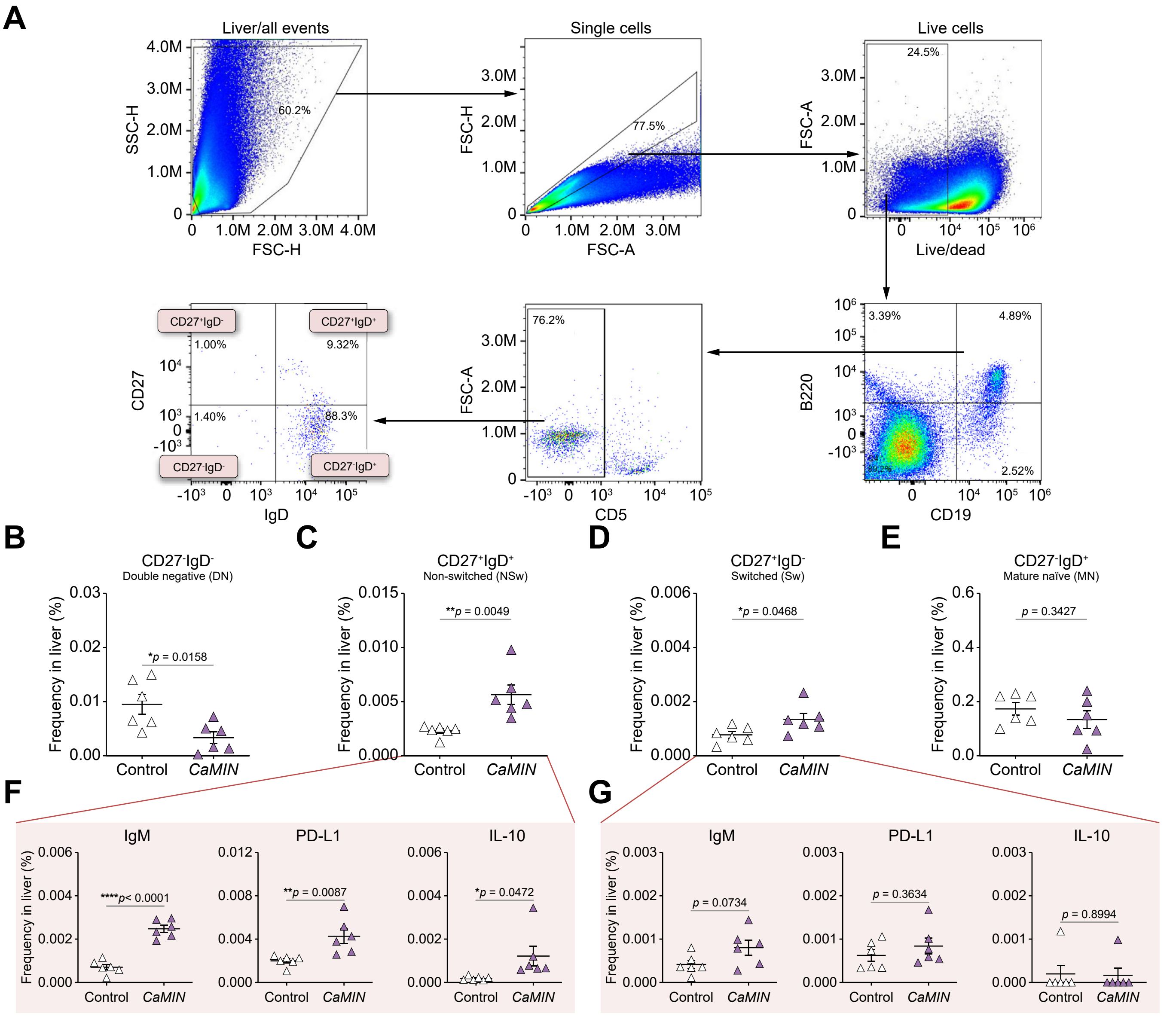

Fig. 4. Increased numbers of CD27$^+$IgD$^+$ NSw MBCs with elevated local IgM, PD-L1, and IL-10 expression in the livers of HCC/*CaMIN* mice. (A) Gating strategy to identify and characterize the phenotype of MBCs in the liver. (B–E) Frequencies of (B) CD27$^-$IgD$^-$ DN, (C) CD27$^+$IgD$^+$ NSw, (D) CD27$^+$IgD$^-$ Sw, and (E) CD27$^-$IgD$^+$ MN MBCs. (F, G) Frequencies of IgM$^+$-, PD-L1$^+$- and IL-10$^+$-expressing (F) CD27$^+$IgD$^+$ NSw and (G) CD27$^+$IgD$^-$ Sw MBCs. The data were analyzed using the unpaired Student's *t* test. The data were shown as the mean ± SEM, n = 6. *p <0.05, **p <0.01, ****p <0.0001. Fig. S4 shows the MASLD and HCC/*NRAS*^{G12V}/*p19*$^{Arf-/-}$ models. DN, double-negative; FSC-A, forward scatter area; FSC-H, forward scatter height; HCC, hepatocellular carcinoma; MBCs, memory B cells; NSw, non-switched; PD-L1, programmed death-ligand 1; SSC-H, side scatter height; Sw, switched.

the numbers of CD27$^+$IgD$^+$ NSw (Fig. 4C) and CD27$^+$IgD$^-$ Sw (Fig. 4D) MBCs was detected in the livers of HCC/*CaMIN* mice compared with control mice. No changes were observed in the frequency of CD27$^-$IgD$^+$ MN MBCs (Fig. 4E). The levels of MBCs were significantly decreased in the blood of mice with HCC/*CaMIN* compared with that of control mice (data not shown).

To address the functional characteristics of MBCs, we further examined the expression of IgM and of the immunosuppressive molecules PD-L1 and IL-10 in highly increased MBC populations. We detected significantly elevated frequencies of IgM$^+$-, PD-L1$^+$-, and IL-10$^+$-expressing CD27$^+$IgD$^+$ NSw MBCs in HCC/*CaMIN* mice compared with those in controls (Fig. 4F). CD27$^+$IgD$^-$ Sw MBCs showed only moderate but not significant increases in IgM and PD-L1 expression and few changes in IL-10 expression (Fig. 4G).

We further investigated the presence of MBCs in mice with MASLD (Fig. S4A–D) and another HCC mouse model, HCC/*NRAS*^{G12V}/*p19*$^{Arf-/-}$ (Fig. S4E–H). Interestingly, the numbers of all the MBC subsets were shown to be elevated (but not significantly) in the livers of the HCC/*NRAS*^{G12V}/*p19*$^{Arf-/-}$ mice (Fig. S4E–H), but not those of the mice with MASLD (Fig. S4A–D). Neither MASLD nor HCC/*NRAS*^{G12V}/*p19*$^{Arf-/-}$ mice exhibited elevated expression of PD-L1 or IL-10 (data not shown).

In summary, CD27$^+$IgD$^+$ NSw MBCs seem to have the strongest inhibitory effect shown by PD-L1 and IL-10

expression among all MBCs in mice with aggressive HCC/*CaMIN*. In addition, CD27$^+$IgD$^+$ NSw MBCs showed an increase in expression of the IgM receptor in mice with aggressive HCC/*CaMIN*.

Increases of PD-L1$^+$-, IL-10$^+$- and IgM$^+$IgD$^+$-expressing CD19$^+$B220$^+$CD138$^+$ PBs in livers of mice with MASLD and HCC

We next investigated the phenotype of CD138-expressing B cells in mice with MASLD and HCC. Using the gating strategy shown in Fig. 5A, we identified CD19$^+$B220$^+$CD138$^+$ PBs in the murine livers. A significant increase in the number of CD19$^+$B220$^+$CD138$^+$ PBs was detected in HCC/*CaMIN* livers (Fig. 5B). We further tested the expression of inhibitory molecules (PD-L1 and IL-10) on PBs. We detected highly elevated frequencies of PD-L1$^+$- and IL-10$^+$-expressing CD19$^+$B220$^+$CD138$^+$ B cells in the livers of mice with HCC/*CaMIN* (Fig. 5C, D). A significant increase in IgM$^+$IgD$^-$-, IgM$^+$IgD$^+$-, and IgA$^-$IgD$^+$- (Fig. 5E–H), but not IgA$^+$IgD$^-$-expressing (Fig. 5H) CD19$^+$B220$^+$CD138$^+$ B cells, was detected in the livers of HCC/*CaMIN* mice.

We next identified CD19$^+$B220$^+$CD138$^+$ PCs in mice with MASLD (Fig. S5A–G) and in HCC/*NRAS*^{G12V}/*p19*$^{Arf-/-}$ (Fig. S5H–N). In both, MASLD (Fig. S5A, not significant) and HCC/*NRAS*^{G12V}/*p19*$^{Arf-/-}$ mice (Fig. S5H), an increase in

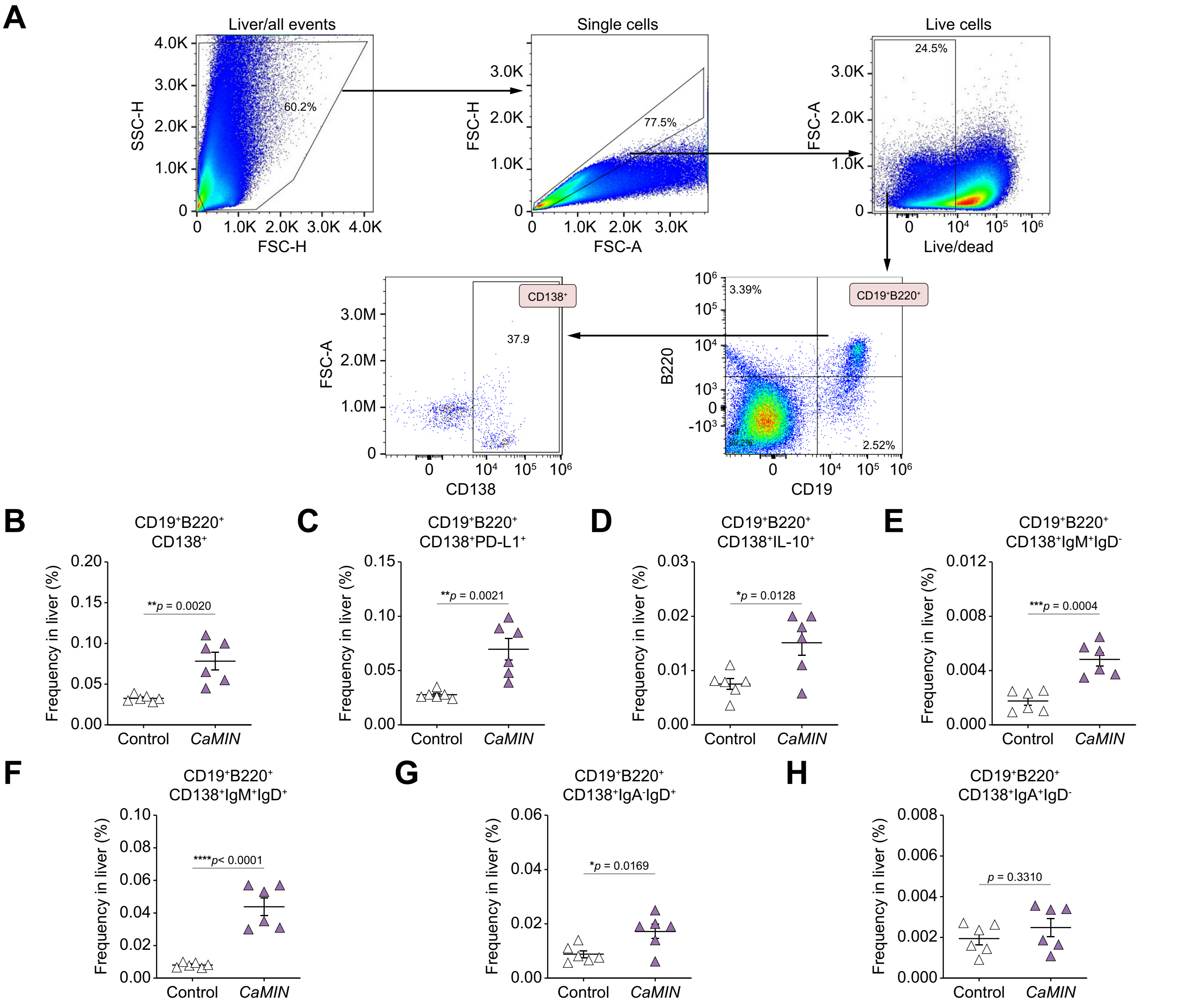

Fig. 5. There was a strong increase in the frequencies of PD-L1$^+$-, IL-10$^+$-, and IgM$^+$IgD$^+$-expressing CD19$^+$B220$^+$CD138$^+$ PBs in the livers of HCC/*CaMIN* mice. (A) Gating strategy. (B) Frequencies of CD19$^+$B220$^+$CD138$^+$ PBs. (C, D) Frequencies of (C) PD-L1$^+$- and (D) IL-10$^+$-expressing CD19$^+$B220$^+$CD138$^+$ PBs. (E–H) Frequencies of (E) IgM$^+$IgD$^-$-, (F) IgM$^+$IgD$^+$-, (G), IgA$^-$IgD$^+$-, and (H) IgA$^+$IgD$^-$-expressing CD19$^+$B220$^+$CD138$^+$ PBs. The data were analyzed using the unpaired Student's *t* test. The data are shown as the mean ± SEM, n = 6. *p <0.05, **p <0.01, ***p <0.001, ****p <0.0001. Fig. S5 shows the MASLD and HCC/*NRAS*^{G12V}/*p19*$^{Arf-/-}$ models. FSC-A, forward scatter area; FSC-H, forward scatter height; HCC, hepatocellular carcinoma; PBs, plasmablasts; PD-L1, programmed death-ligand 1; SSC-H, side scatter height.

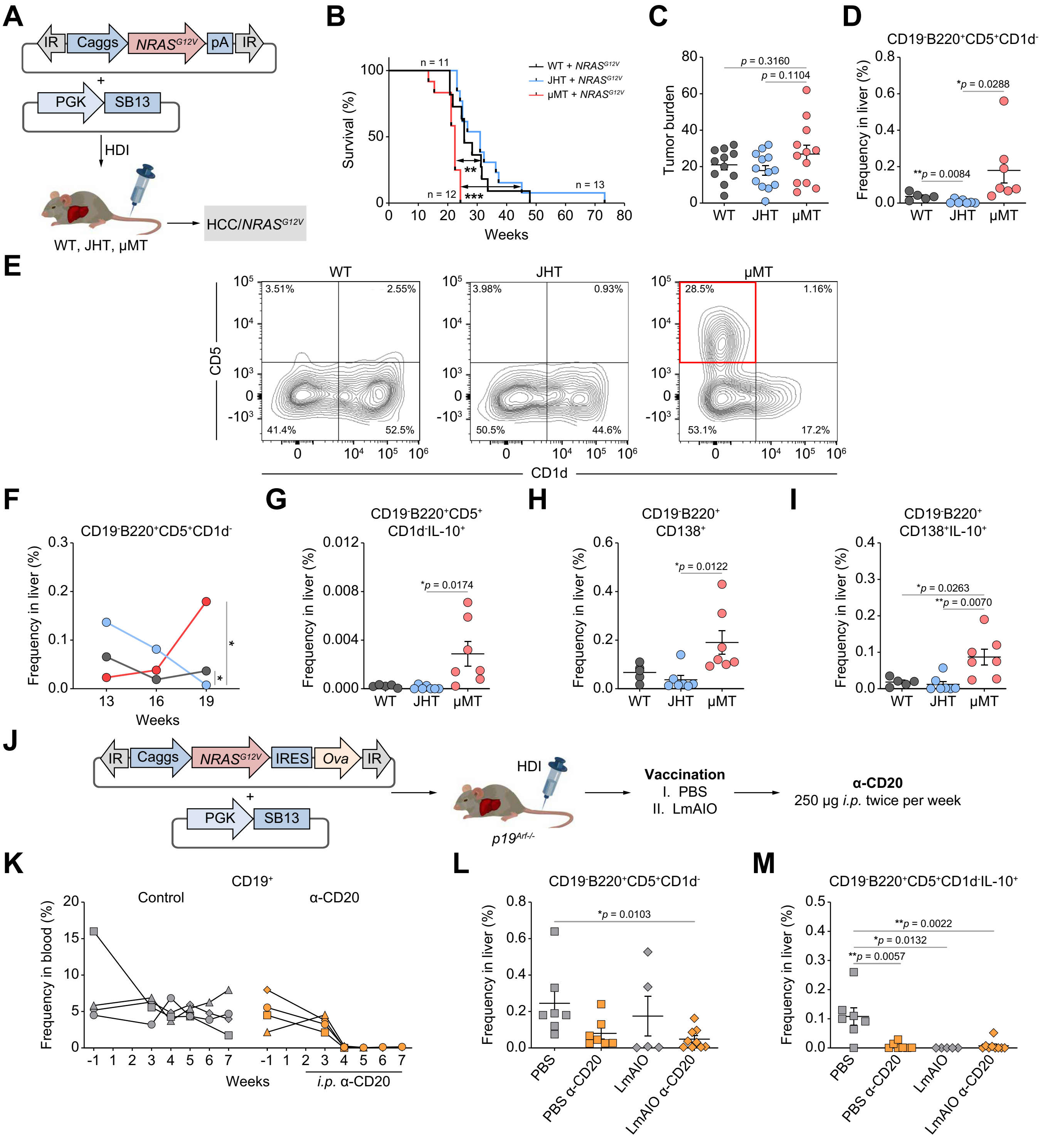

Fig. 6. An increase in the numbers of CD19$^-$B220$^+$CD5$^+$CD1d$^-$ and CD19$^-$B220$^+$CD138$^+$ cell subsets expressing IL-10 in the liver correlates with HCC/*NRAS^{G12V}/p19$^{Arf-/-}$* progression in B-cell-deficient µMT mice. A reduction in CD19$^-$B220$^+$CD5$^+$CD1d$^-$ Bregs as well as in IL-10 on these cells correlated with protection against HCC-Ova/*NRAS^{G12V-}Ova/p19$^{Arf-/-}$* development. (A) *NRAS^{G12V}* transposon constructs were codelivered with a transposase (*SB13*) into C57BL/6J (WT) and B-cell-deficient mice (JHT and µMT) via HDI. (B) Kaplan−Meier survival curves of WT, JHT and µMT mice. (C) Tumor burdens in JHT, µMT, and WT mice. (D) Frequencies of CD19$^-$B220$^+$CD5$^+$CD1d$^-$ cells in the livers of WT, JHT, and µMT mice at week 19 after HDI. (E) Representative FACS plots of CD19$^-$B220$^+$CD5$^+$CD1d$^-$ cells in the livers of WT, JHT, and µMT mice at week 19 after HDI. (F) Kinetics of the frequencies of CD19$^-$B220$^+$CD5$^+$CD1d$^-$ cells monitored at weeks 13, 16, and 19 after HDI in the livers of WT and B-cell-deficient JHT and µMT mice. (G–I) Frequencies of (G) CD5$^+$CD1d$^-$IL-10$^+$, (H) CD138$^+$, and (I) CD138$^+$IL-10$^+$ cells in the livers of WT, JHT, and µMT mice at week 19 after HDI. (J) Experimental setup to study the therapeutic potential of α-CD20 and LmAIO administered either alone or in combination to *p19$^{Arf-/-}$* mice harboring HCC-Ova. (K) Kinetics of CD19$^+$ B cells monitored in the blood of HCC-Ova/*NRAS^{G12V-}Ova/p19$^{Arf-/-}$* mice. (L-M) Frequencies of (L) CD19$^-$B220$^+$CD5$^+$CD1d$^-$ and (M) CD19$^-$B220$^+$CD5$^+$CD1d$^-$IL-10$^+$ Breg cells at the survival endpoint in the livers of HCC-Ova/*NRAS^{G12V-}Ova/p19$^{Arf-/-}$* mice treated with α-CD20 and LmAIO either alone or in combination. The data were analyzed using the unpaired Student's *t* test. The data are shown as the mean ± SEM, n = 5–13. $^*p$ <0.05, $^{**}p$ <0.01, $^{***}p$ <0.001. Bregs, B regulatory cells; Caggs, synthetic CAG; IR, inverted repeats; HCC, hepatocellular carcinoma; HDI, hydrodynamic tail vein injection; IR, inverted repeats; IRES, internal ribosome entry site; LmAIO, *Listeria monocytogenes ΔactA/ΔinlB + Ova*; Ova, ovalbumin; pA, polyadenylation site; PGK, phosphoglycerate kinase promoter; *SB13*, *Sleeping Beauty 13*; WT, wild-type.

$CD19^+B220^+CD138^+$ PBs was observed, similar to the findings in the HCC/*CaMIN* mouse model. However, to a lesser extent than in the aggressive HCC/*CaMIN* group, increased frequencies of PD-L1$^+$- and IL-10$^+$-expressing cells were observed in the livers of mice with MASLD (Fig. S5B and Fig. S5C, neither significant) and HCC/*NRAS*^{G12V} (Fig. S5I, significant and Fig. S5J, not significant). Importantly, elevated PD-L1 expression seemed to correlate with disease severity and was more pronounced in both HCC models (Fig. 5C and Fig. S5I) than in controls. In addition, a significant increase in IgM^+IgD^+ PBs was detected in both MASLD (Fig. S5E) and HCC/*NRAS*^{G12V}/*p19*$^{Arf-/-}$ mice (Fig. S5L), which strongly correlated with the findings in the HCC/*CaMIN* model (Fig. 5F).

In summary, the obtained data indicated a strong increase in the number of PD-L1$^+$, IL-10$^+$, and IgM^+IgD^+ $CD19^+B220^+CD138^+$ PBs in the livers of mice with MASLD and HCC, which correlated with disease severity.

Increase of IL-10$^+$-expressing $CD19^-B220^+CD5^+CD1d^-$ and $CD19^-B220^+CD138^+$ cell subsets in the liver correlates with HCC progression in B-cell-deficient μMT mice

We next validated the impact of B cells on the progression of HCC in B-cell knockout mice (μMT[32] and JHT[33]). The *NRAS*^{G12V} transposon construct was delivered along with *SB13* transposase into WT or B-cell-deficient mice (μMT and JHT) to induce HCC development (Fig. 6A). We subsequently monitored tumor progression over time and the survival of these mice. Compared with those of B-cell-deficient JHT mice and control WT mice, the survival of B-cell-deficient μMT mice strongly and rapidly decreased upon induction with HCC/*NRAS*^{G12V} (Fig. 6B). The tumor burden was greater in μMT mice than in B-cell-deficient JHT and control WT mice (Fig. 6C).

Importantly, FACS analysis revealed a significant increase in $CD19^-B220^+CD5^+CD1d^-$ cells in the livers of μMT mice at week 19 after tumor induction (Fig. 6D, E). In addition, we performed kinetic studies and determined the frequencies of $CD19^-B220^+CD5^+CD1d^-$ cells in the livers of all three tested murine strains 13, 16, and 19 weeks after hydrodynamic tail vein injection (HDI). The increase in the number of $CD19^-B220^+CD5^+CD1d^-$ cells correlated with the decrease in survival in μMT mice at week 19 (Fig. 6F). Interestingly, we further detected elevated frequencies of IL-$10^+CD19^-B220^+CD5^+CD1d^-$ cells in the livers of μMT mice at week 19 after HDI (Fig. 6G). Additionally, compared with JHT and WT mice, μMT mice showed a significant increase in the number of $CD19^-B220^+CD138^+$ cells (Fig. 6H). The latter cell subset demonstrated a significant upregulation of IL-10 expression in the livers of μMT mice compared with those of their JHT and WT counterparts (Fig. 6I).

To further investigate the functional role of B cells, we performed B-cell depletion therapy using a monoclonal α-CD20 antibody, as previously described.[11,13] We performed the B-cell depletion therapy either alone or in combination with administration of an experimental cancer vaccine comprising the double-deletion vaccine strain LmAIO for *in vivo* application in an HCC-ovalbumin (Ova) model in *p19*$^{Arf-/-}$ mice, as described in our previous research[13] (Fig. 6J–M). Vaccination with either LmAIO or the combination of LmAIO+α-CD20 was highly protective against HCC development (92–70% protection), whereas monotherapy with α-CD20 protected 45% of mice against HCC development.[13] Importantly, we observed a reduction in the $CD19^-B220^+CD5^+CD1d^-$ Breg cell population in all the protected groups (Fig. 6L). In particular, a significant reduction in the number of $CD19^-B220^+CD5^+CD1d^-$ Bregs occurred upon treatment with the combination of LmAIO+α-CD20 compared with those in the PBS control group (Fig. 6L). Interestingly, we further detected a significant reduction of IL-10$^+$-expressing $CD19^-B220^+CD5^+CD1d^-$ Bregs in PBS+α-CD20-, LmAIO-, and LmAIO+α-CD20-treated animals compared with that in the PBS control (Fig. 6M). Importantly, the reduction in the number of $CD19^-B220^+CD5^+CD1d^-$ Bregs correlated with survival (see Fig. 7D in Hochnadel, Hoenicke *et al.*).[13]

In summary, the numbers of cells with the $CD19^-B220^+CD5^+CD1d^-$ and $CD19^-B220^+CD138^+$ phenotypes and expressing IL-10 were significantly increased in the livers of μMT mice and correlated with HCC progression. Furthermore, B-cell depletion therapy and protective therapeutic vaccination (combination) therapy led to a reduction in numbers of the $CD19^-B220^+CD5^+CD1d^-$ Breg subset as well as in the expression of IL-10 on these cells and improved survival in mice harboring HCC.

Elevated levels of $CD19^+CD5^+$ and $CD19^+CD5^+CD1d^+$ Breg cells in the livers of mice with MASLD and HCC

We next analyzed the expression of CD19, CD5, and CD1d in liver tissues obtained from mice with MASLD, HCC/*CaMIN* and HCC/*NRAS*^{G12V}/*p19*$^{Arf-/-}$ using immunohistochemistry (IHC) and immunofluorescence (IF) (Fig. 7 and Fig. S6).

Histopathological examination of liver tissues obtained from HCC/*NRAS*^{G12V} mice revealed abundant immune infiltration in the tumor tissue and the presence of CD19$^+$, CD5$^+$, and CD1d$^+$ cells (Fig. 7A).

Next, we evaluated the coexpression of CD19, CD5, and CD1d in B cells using multicolor IF staining (Fig. 7B, C). Significantly greater numbers of $CD19^+CD5^+$ and $CD19^+CD5^+CD1d^+$ Bregs were found in the livers of HCC/*NRAS*^{G12V} mice compared with controls (Fig. 7D, E). Elevated levels of $CD19^+CD5^+$ (Fig. 7F, not significant) and $CD19^+CD5^+CD1d^+$ Bregs (Fig. 7G, significant) were detected in the livers of HCC/*CaMIN* mice compared with controls. Additionally, increased (not significant) numbers of $CD19^+CD5^+$ and $CD19^+CD5^+CD1d^+$ B cells were detected in the livers of mice with MASLD (Fig. S6A–C).

In summary, histopathological examination and multicolor IF staining demonstrated the presence of increased numbers of $CD19^+CD5^+$ and $CD19^+CD5^+CD1d^+$ Breg cells in mice with HCC and MASLD.

$CD19^-B220^+CD5^+CD1d^-$ Bregs, $CD19^+B220^+CD5^+CD1d^+$ B10 Bregs, and $CD19^+B220^+CD27^+IgD^+$ NSw MBCs demonstrate the most immunosuppressive phenotype based on IL-10 and PD-L1 expression in HCC/*CaMIN* mice.

To define the most immunosuppressive phenotype among the B cell subsets found in the mice in this study, we prepared a pie chart diagram that depicts the proportions of IL-10$^+$ and PD-L1$^+$ B cell subsets normalized to those of the respective controls in the livers of HCC/*CaMIN* mice (Fig. S6D, E, respectively). Based on the obtained results, we can conclude that three B cell subsets can potentially be characterized as the most relevant/immunosuppressive. In particular, $CD19^-B220^+CD5^+CD1d^-$ Bregs (with the highest expression of

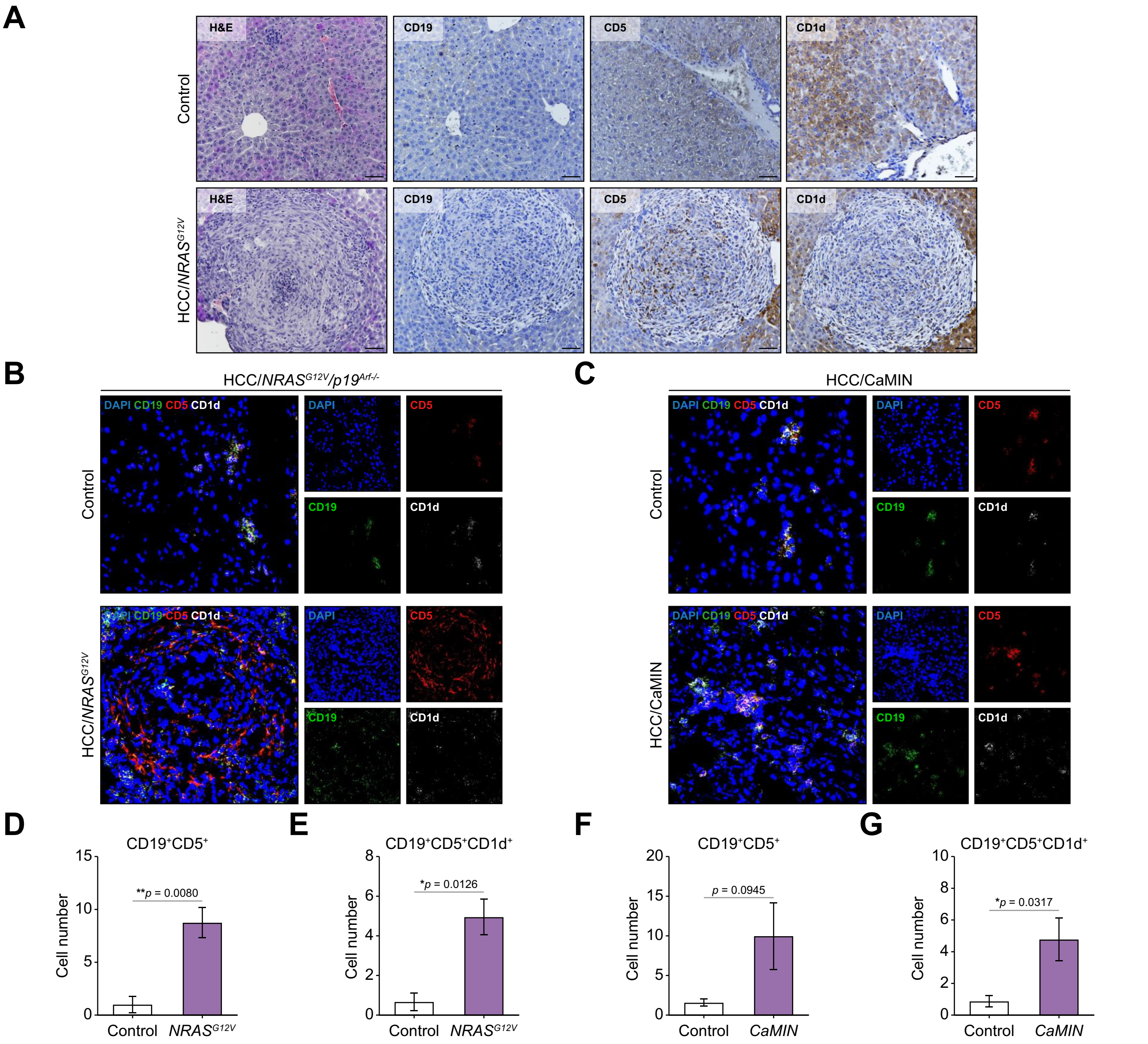

Fig. 7. Histopathological examination and multicolor IF staining revealed elevated numbers of CD19$^+$CD5$^+$ and CD19$^+$CD5$^+$CD1d$^+$ Bregs in the livers of HCC/*NRAS*^{G12V}/*p19*$^{Arf-/-}$ and HCC/*CaMIN* mice. (A) Representative images of H&E and IHC staining for CD19, CD5, and CD1d expression in the liver tissues of the HCC/*NRAS*^{G12V}/*p19*$^{Arf-/-}$ mice. Scale bar, 100 µm. (B, C) Representative IF images of frozen liver sections obtained from (B) HCC/*NRAS*^{G12V}/*p19*$^{Arf-/-}$ and (C) HCC/*CaMIN* mice stained with CD19 (green), CD5 (red), and CD1d (white) antibodies and counterstained with DAPI (blue). (D, E) Quantification of (D) CD19$^+$CD5$^+$ and (E) CD19$^+$CD5$^+$CD1d$^+$ cells in the livers of mice with HCC/*NRAS*^{G12V}/*p19*$^{Arf-/-}$. (F, G) Quantification of (F) CD19$^+$CD5$^+$ and (G) CD19$^+$CD5$^+$CD1d$^+$ cells in the livers of mice with HCC/*CaMIN*. The data were analyzed using the unpaired Student's *t* test. The data are shown as the mean ± SEM, n = 5–6. *p <0.05, **p <0.01. Fig. S6 shows the MASLD model. Bregs, B regulatory cells; HCC, hepatocellular carcinoma; IF, immunofluorescence.

immunomodulatory molecules), CD19$^+$B220$^+$CD5$^+$CD1d$^+$ B10 Bregs, and CD19$^+$B220$^+$CD27$^+$IgD$^+$ NSw MBCs exhibited the most immunosuppressive phenotype *in vivo* based on their PD-L1 and IL-10 expression in murine HCC/*CaMIN* tissues.

Identification and characterization of the inflamed ('hot') and non-inflamed ('cold') subtypes of human HCC

Next, we analyzed the liver tissues obtained from patients with HCC upon HCC resection. The clinicopathologic characteristics of the patients in the human HCC cohort are shown in Table S1. Histopathological examination of HCC samples revealed that most patients (75%) had a second grade of differentiation (G2) according to the classification system for malignant tumors (tumor, nodes, metastasis [TNM]).[34] Microvascular invasion was detected in 18.75% of the patients with HCC (Table S1).

Using histological assays and based on the assessment of the immune infiltration score (Fig. 8A), we classified the available HCC tissues into two immune subclasses: inflamed ('hot') and non-inflamed ('cold'), as previously described.[6,7] The inflamed HCC subtype is characterized by a

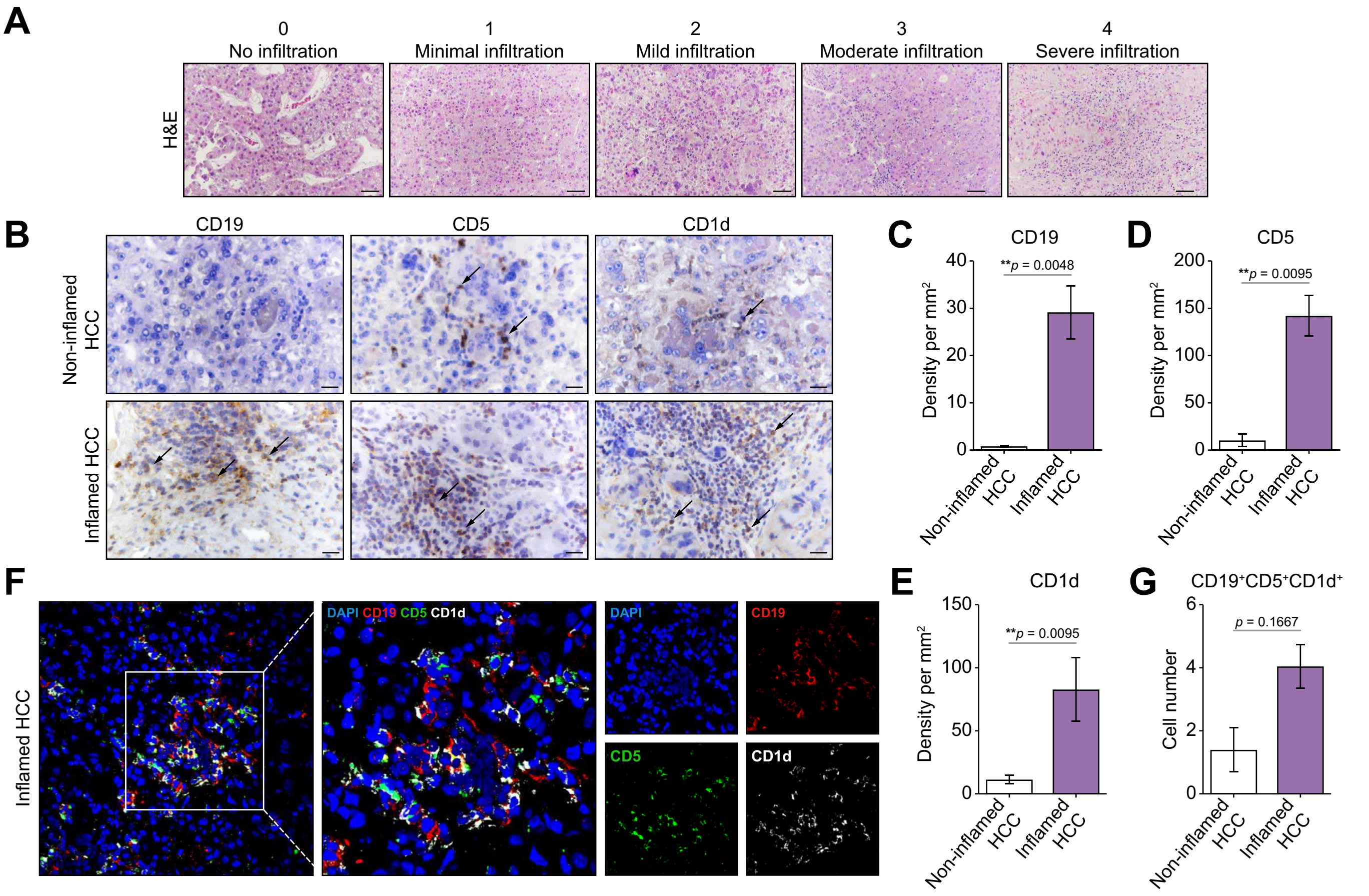

Fig. 8. The inflamed subtype of human HCC is characterized by the presence of high numbers of CD19⁺-, CD5⁺-, and CD1d⁺-expressing B cells. (A) Representative H&E images of human HCC liver tissues and immune infiltration assessment scores (non-inflamed HCC [≤2 infiltration score]; inflamed HCC [≥3 infiltration score]). (B) Representative images of IHC of CD19, CD5, and CD1d expression in non-inflamed and inflamed human HCC tissues. Scale bar, 100 μm. (C–E) Density of cellular markers (C) CD19, (D) CD5, and (E) CD1d in human non-inflamed and inflamed HCC tissues. The data were analyzed using the Mann−Whitney nonparametric test, n = 10. $^{**}p$ <0.01. (F) Representative IF images of frozen liver sections from patients with inflamed HCC stained with CD19 (red), CD5 (green), and CD1d (white) antibodies and counterstained with DAPI (blue). (G) Quantification of $CD19^+CD5^+CD1d^+$ B cells in human non-inflamed and inflamed HCC tissues. The data were analyzed using the Mann−Whitney nonparametric test, n = 7. $^{**}p$ <0.01. HCC, hepatocellular carcinoma; IHC, immunohistochemistry; IF, immunofluorescence.

significantly greater immune infiltration score than the non-inflamed HCC subtype. The number of patients with inflamed and non-inflamed HCC was equal in the cohort (50%) (Table S1). Furthermore, we detected TLSs in human HCC tissues (Fig. S6F). Clear aggregates of lymphocytic immune cells closely associated with hepatocyte tumor cells were observed (Fig. S6F). According to the literature, TLSs are classified as early, primary, or secondary (Fig. S6F).[7,35]

The inflamed subtype of human HCC demonstrates the presence of high amounts of $CD19^+$, $CD5^+$, and $CD1d^+$ B cells

We further analyzed the expression of CD19, CD5, and CD1d in liver tissues obtained from HCC patients (Fig. 8B–E). We performed IHC analysis of the available HCC cohort divided into inflamed and non-inflamed HCC groups as described in the previous section.

In the inflamed HCC subtype, a high number of $CD19^+$ cells were observed, whereas non-inflamed HCC displayed a lack of $CD19^+$ B cell infiltration (Fig. 8B, C). $CD5^+$ and $CD1d^+$ cells were abundant and spatially distributed in the different compartments of the tumor (Fig. 8B). They were noted both in the tumor stroma and parenchyma (Fig. 8B and D, E).

We further analyzed the presence of Bregs in HCC liver tissues using multicolor IF staining while assessing the coexpression of CD19, CD5, and CD1d. A greater number of $CD19^+CD5^+CD1d^+$ Bregs was detected in the inflamed subtype of human HCC than in the non-inflamed subtype of human HCC (Fig. 8F, G, not significant).

In summary, the inflamed subtype of HCC exhibited a notable presence of $CD19^+$ cells accompanied by significant $CD5^+$ and $CD1d^+$ expression in the tumor area. Furthermore, $CD19^+CD5^+CD1d^+$ Bregs were detected in the inflamed subtype of human HCC using multicolor IF analysis.

The inflamed subtype of human HCC has increased numbers of $CD19^+CD5^+IL\text{-}10^+$, $CD19^+CD5^+PD\text{-}L1^+$, and $CD19^+IgM^+IgD^+$ B cells

Next, we aimed to analyze the expression of IL-10, PD-L1, and IgD/IgM in human HCC liver tissue using IHC and multicolor IF staining (Fig. S7).

Compared with the non-inflamed HCC subtype, the inflamed HCC subtype was characterized by significantly more IL-10$^+$ and PD-L1$^+$ lymphocytes (Fig. S7A–C), as confirmed by experienced pathologists. IgD$^+$ cells exhibited a single-cell scattered pattern of distribution and had significantly elevated numbers in the parenchymal and stromal regions of the HCC tumor core as well as in the surrounding tissue in the inflamed HCC subtype (Fig. S7A, D).

We further examined the coexpression of CD19, CD5, and IL-10 in human HCC liver samples using IF. A significantly greater number of CD19$^+$CD5$^+$IL-10$^+$ B cells was observed in the inflamed HCC liver tissue than in the non-inflamed HCC liver tissue (Fig. S7E, F). Furthermore, significantly greater numbers of CD19$^+$CD5$^+$PD-L1$^+$ (Fig. S7G) and CD19$^+$IgM$^+$IgD$^+$ B cells (Fig. S7H) were observed in the inflamed HCC subtype than in the non-inflamed HCC subtype. The number of CD19$^+$CD5$^+$IgD$^+$ B cells was also increased in the inflamed HCC subtype but not significantly (Fig. S7I).

Taken together, increased numbers of CD19$^+$CD5$^+$IL-10$^+$, CD19$^+$CD5$^+$PD-L1$^+$, and CD19$^+$IgM$^+$IgD$^+$ B cells were detected in inflamed HCC liver tissue compared with non-inflamed HCC liver tissue in humans.

The upregulation of IgM$^+$IgD$^+$- and IL-10$^+$-expressing CD19$^+$CD20$^+$ B cells, CD19$^+$CD20$^+$CD5$^+$CD1d$^+$ Bregs, and IL-10$^+$- and IgM$^+$-expressing CD19$^+$CD20$^+$CD27$^+$IgD$^+$ NS MBCs in the blood of patients with MASLD strongly correlated with the murine MASLD data

Because of limited access to liver tissues from patients with MASLD (Table S2), we further performed B cell phenotyping using the peripheral blood mononuclear cells (PBMCs) of patients with MASLD via multicolor FACS and compared the results with those obtained for HFD-fed mice (MASLD group).

We first compared the total number of CD19$^+$CD20$^+$ B cells in the blood of humans and mice with MASLD (Fig. S8A, B, respectively) and detected that the numbers of these cells were only moderately but not significantly elevated in both humans and mice with MASLD. Importantly, a substantial increase in the number of IL-10$^+$CD19$^+$CD20$^+$ B cells was detected in both species (Fig. S8A, B). Furthermore, an increase in the number CD19$^+$CD20$^+$IgM$^+$IgD$^+$ B cells was observed in both groups and was significant in mice with MASLD (Fig. S8A, B).

Next, we examined CD19$^+$CD20$^+$CD5$^+$CD1d$^+$ Bregs in the blood of patients with MASLD (Fig. S8C) and mice with MASLD (Fig. S8D). Breg numbers showed only moderate but not significant increases in both species (Fig. S8C, D). A significant increase in the number of IL-10$^+$-expressing CD19$^+$CD20$^+$CD5$^+$CD1d$^+$ Bregs was detected in the blood of patients with MASLD compared with that in the blood of healthy controls (Fig. S8C), and the same trend was observed in mice (Fig. S8D). A non-significant increase in the number of IgM$^+$IgD$^+$-expressing CD19$^+$B220$^+$CD5$^+$CD1d$^+$ Bregs was further detected in the blood of patients with MASLD and mice with MASLD (Fig. S8C, D).

A strong increase in the number of CD19$^+$CD20$^+$CD27$^+$IgD$^+$ NSw MBCs was observed in the blood of patients with MASLD compared with controls (Fig. S8E), which fully correlated with murine data (Fig. 8F). Furthermore, NSw MBCs, which significantly overexpressed IL-10 and IgM, were detected in the blood of patients with MASLD (Fig. S8E). The frequencies of IgM$^+$-expressing cells were also strongly increased in the blood of mice with MASLD, whereas the frequency of IL-10-expressing cells remained unchanged (Fig. S8F).

The frequencies of CD19$^+$CD20$^+$CD138$^+$ PBs and IgM$^+$IgD$^+$-expressing CD19$^+$CD20$^+$CD138$^+$ PBs were significantly decreased in the blood of patients with MASLD (Fig. S8G). However, the level of IL-10$^+$-expressing CD19$^+$CD20$^+$CD138$^+$ PBs significantly increased in the blood of patients with MASLD (Fig. S8G). Similar non-significant results were observed in the blood of mice with MASLD (Fig. S8H).

In summary, our study reveals similar results in mice and humans with MASLD. We detected IgM$^+$IgD$^+$ and significant overexpression of IL-10 on total CD19$^+$CD20$^+$ B cells and on CD19$^+$CD20$^+$CD5$^+$CD1d$^+$ Breg cells and a significant overexpression of IL-10 and IgM on CD19$^+$CD20$^+$CD27$^+$IgD$^+$ NSw MBCs in the blood of patients with MASLD.

Discussion

In the present study, we characterized the phenotypic statuses of several B cell subsets isolated from the livers and blood of mice with MASLD and HCC. In addition, we performed B cell phenotyping on human samples (PBMCs isolated from patients with MASLD and HCC liver tissues obtained after HCC surgery).

We used a range of markers including CD19, B220, CD5, CD1d, CD138, CD27, IgM, IgD, IgA, IL-10, and PD-L1, to comprehensively phenotype B cell subsets.[9,14,16,21] in the liver and other organs. Based on the obtained results, we specifically concentrated on a few subsets (Bregs, PBs, and MBCs) that exhibited the most pronounced changes in comparison with those in the controls.

In aggressive murine HCC (HCC/*CAMIN*), we detected several B cell populations, such as CD19$^-$B220$^+$CD5$^+$CD1d$^-$ Bregs, CD19$^+$B220$^+$CD5$^+$CD1d$^+$ B10 Bregs, CD19$^+$B220$^+$CD27$^+$IgD$^+$ NSw MBCs, and CD19$^+$B220$^+$CD138$^+$ PBs, which all demonstrated a protumorigenic phenotype (upregulated IL-10 and PD-L1).

Our data are in line with those of the recent studies showing that Bregs are suppressors of antitumor immunity in both mice and humans.[14,36] In agreement with the literature, our results clearly demonstrate a strong increase in the numbers of CD19$^+$B220$^+$CD5$^+$CD1d$^+$ (B10) and CD19$^-$B220$^+$CD5$^+$CD1d$^-$ Bregs in mice with MASLD and HCC. Studies using murine models, have suggested that CD19$^+$CD5$^+$CD1d$^{+(hi)}$ B10 cells represent the predominant immunomodulatory B cell subpopulation that secretes IL-10.[17,37] In our study, the elevated expression levels of PD-L1 and IL-10 were correlated with malignant liver disease severity. In line with our findings, recent evidence suggests that activation of the PD-1/PD-L1 pathway represents one mechanism that allows tumors to elude the host immune system.[38,39] The expression of PD-L1 has been associated with poor prognosis in patients with pancreatic cancer and renal cell carcinoma.[40] PD-L1$^+$ Bregs decrease the production of proinflammatory cytokines by PD-1-expressing CD4$^+$ T cells, macrophages, and NK cells.[14] In addition, Bregs can interact with PD-1-expressing follicular helper T cells, resulting in the inhibition of humoral immune responses.[14]

Recently, IgA-producing B cells were shown to modulate cytotoxic CD8$^+$ T cells in a mouse model of HCC.[18] Moreover, IgA has been recognized as a key biomarker of

immunosuppressive B cells.[18,41] Our data further extend the knowledge presented in a study by Shalapour *et al.*[18] We found that secreted IgA levels were strongly upregulated in the plasma of mice harboring aggressive HCC. Furthermore, we provided additional insights into the role of membrane-bound IgM^+IgD^+ B cells in liver diseases. In the context of Bregs in the liver *in situ*, we detected the membrane-bound forms of immunoglobulins, as these immunoglobulins might play a role in cell-to-cell interactions and immune regulation within the liver microenvironment. Using an ELISA, we measured the levels of secreted immunoglobulins in the blood, which are involved in systemic immune responses. The lack of correlation between the levels of immunoglobulins on Bregs and in the blood could be explained by the different forms of immunoglobulins measured in the liver and plasma.

In this study, we observed a high upregulation of IgM^+- and IgD^+-expressing $CD19^+B220^+CD5^+CD1d^+$ and $CD19^-B220^+CD5^+CD1d^-$ Bregs in the livers of MASLD and HCC mice. IgM and IgD molecules were further detected on $CD19^+B220^+CD5^+CD27^+$ NSw MBCs and $CD19^+B220^+CD138^+$ PBs in murine liver disease. In addition, the abovementioned B cell subsets also highly overexpressed immunosuppressive PD-L1 and IL-10. These findings suggest that the role of B cells in MASLD and HCC may be mediated not only through secreted factors, such as IgA but also through direct cell-to-cell interactions within the tissue microenvironment.

Our data further extend the knowledge regarding MBCs during cancer progression. A significantly greater frequency of $CD27^+IgD^-$ MBCs was found in the peripheral blood of patients with colorectal cancer.[42] In our study, we demonstrated that $CD27^+IgD^+$ NSw MBCs exhibit the most pronounced inhibitory phenotype among all MBCs in aggressive HCC/*CaMIN*, characterized by elevated levels of PD-L1 and IL-10 expression. In addition, $CD27^+IgD^+$ NSw MBCs showed an increase in IgM receptor expression in the livers of mice harboring aggressive HCC/*CaMIN*.

In some studies, high rates of plasma cell and $CD138^+$ B cell infiltration into tumors were associated with shorter recurrence-free survival in patients with invasive breast carcinoma.[43] In addition, $CD138^{hi}$ regulatory plasma cells can produce IL-10 through infection and inflammation.[44] Our findings are in line with the literature, and showed elevated numbers of PD-$L1^+$-, IL-10^+- and IgM/IgD^+-expressing $CD19^+B220^+CD138^+$ PBs in HCC-bearing mice.

Survival experiments in B-cell-deficient μMT mice, which lack the expression of membrane-bound IgM on B cells, demonstrated a strong decrease in survival upon the induction of HCC/$NRAS^{G12V}$ in comparison with that in B-cell-deficient JHT and WT mice. Recently, it was reported that even in the absence of the mu-chain, these μMT mice are leaky and can produce antibodies.[45] Through the use of the alpha constant region chain instead of the mu constant region chain, μMT mice exhibit selective development of IgA^+ cells in the absence of IgM or IgD heavy chain expression.[46] Notably, these IgA^+ cells predominantly develop within the gastrointestinal tract in μMT mice.[19] In our study, we observed that the numbers of cells with $CD19^-B220^+CD5^+CD1d^-$ and $CD19^-B220^+CD138^+$ phenotypes were significantly increased in the livers of μMT mice with HCC. This finding implies that these cell subsets might play a detrimental role in tumor development.

Additionally, in the current study, we showed that the B-cell depletion therapy and protective therapeutic vaccination (combination) therapy led to a reduction in the number of the $CD19^-B220^+CD5^+CD1d^-$ Breg subset, as well as in the level of IL-10 expression on these cells, and improved survival in mice harboring HCC. Importantly, this B cell subset, $CD19^-B220^+CD5^+CD1d^-$ Bregs, exhibited the most immunosuppressive phenotype *in vivo* based on its PD-L1 and IL-10 expression in murine HCC/*CAMIN*, as demonstrated in the pie charts (graphical abstract). Our *in vivo* findings suggest that specific targeting of these cells could be a promising therapeutic option for HCC treatment. Importantly, these findings were observed in preclinical mouse models, and further research is needed to validate the efficacy and safety of B-cell depletion therapy, alone or in combination with a *Listeria*-based vaccine, for clinical applications.

An increase in Bregs was predominantly observed within the liver tissues of MASLD and HCC mice, whereas in the blood of HCC/*CaMIN* mice, all tested Breg populations exhibited decreased numbers, indicating active B cell migration. Furthermore, our study revealed a significant increase in $CXCR5^+$ Bregs in the liver during HCC/*CaMIN* progression. CXCR5 is a chemokine receptor, that plays a pivotal role in B cell migration toward B cell follicles in secondary lymphoid organs.[30,47] For Conversely, the blood and spleen did not exhibit significant increases in the number of $CXCR5^+$-expressing Bregs, indicating a liver-specific migration pattern. Interestingly, with the increase in the number of $CXCR5^+$ Bregs, we also detected TLSs in the livers of both mice and humans with HCC. CXCL13, a ligand of the CXCR5 receptor, is a chemotactic protein for B cells that recruits $CXCR5^+$ B cells to tumor tissues, thereby enhancing tumor immunity.[47] A few reports regarding breast and lung cancers have shown that the CXCL13/CXCR5 axis attracts B cells to form TLSs at peritumoral or tumor sites.[48,49] Recent studies have reported prominent overexpression of CXCL13 in liver cancer tissues and the serum of HCC patients.[47] This evidence supports the notion that increased CXCR5 expression on B cells facilitates their migration from the blood to the liver, contributing to the development and function of TLSs in pathological liver conditions. It remains to be confirmed in follow-up studies whether CXCL13 plays a role in the homing of $CXCR5^+$ Bregs to HCC livers.

By comparing data obtained from mice to data obtained from patients with HCC, our analysis of liver tissues from HCC patients revealed that the inflamed subtype of HCC is characterized by a high abundance of $CD19^+CD5^+$ and $CD1d^+$ cells. Furthermore, the inflamed subtype exhibited increased levels of IL-10- and PD-L1-expressing lymphocytes, and an increased density of IgD^+ cells. These findings are in line with our murine data and highlight the potential of targeting B cells or modulating their activity to regulate the immune response within the tumor microenvironment. In addition, using IF staining, we detected the coexpression of $CD19^+CD5^+CD1d^+$ and $CD19^+CD5^+$IL-10^+ cells in the inflamed subtype of human HCC, similar to murine data. Additionally, we detected significant overexpression of IL-10 and IgM^+IgD^+ on total $CD19^+CD20^+$ B cells, on $CD19^+CD20^+CD5^+CD1d^+$ Bregs and on $CD19^+CD20^+CD27^+$ NSw MBCs in the blood of MASLD patients, which aligns with the findings obtained from MASLD mice. Unfortunately, the data obtained

from the blood of MASLD patients could not be verified with data from liver tissue, because of a lack of liver biopsies from patients with MASLD.

The data obtained in this study underscore the crucial immunosuppressive role of several B cell subsets in the liver microenvironment during liver disease progression. Specific targeting of these cells, for example, using chimeric antigen receptor (CAR) T cells, as shown by several recent studies in different cancer types,[50] could be a therapeutic approach to enhance immune responses against HCC or reduce inflammation associated with MASLD. Consequently, it is also essential to obtain liver biopsies rather than blood samples from MASLD and HCC patients to assess the specific B cell populations and their impact on disease progression.

Interestingly, protumorigenic B cell were found mostly in inflamed human HCC. It has been reported that inflamed HCC is characterized by a high prevalence of immune infiltrates[6,7] and is correlated with a good prognosis in patients.[7,8] Therefore, we assume that targeted neutralization of protumorigenic B cells will further improve the prognosis of patients with HCC; however, the latter needs to first be verified in clinical trials.

Overall, our study provides novel insights into the immunological aspects of MASLD and HCC, emphasizing the involvement of IgM^+IgD^+ Bregs, $CD19^+B220^+CD27^+IgD^+$ NSw MBCs and $CD19^+B220^+CD138^+$ PBs with immunosuppressive characteristics in disease progression. These findings have important implications for the development of targeted immunotherapeutic approaches and hold the potential to improve clinical management and outcomes for individuals with MASLD and HCC. However, further research is needed to understand the mechanisms, clinical relevance, and therapeutic potential of these B cell populations in MASLD and HCC.

In conclusion, our findings demonstrate the involvement of several specific B cell subsets (graphical abstract) in the progression of MASLD and HCC. These subsets include two types of Bregs expressing PD-L1, IL-10, IgM, and IgD, which exert immunosuppressive effects (especially $CD19^-B220^+CD5^+CD1d^-$ Bregs) within the liver. Additionally, two other B cell subsets ($CD27^+IgD^+$ NSw MBCs and $CD19^+B220^+CD138^+$ PBs) were found in MASLD and HCC tissues, which might also play a protumorigenic role in liver disease progression. The specific targeting of these B cell subsets using for example a CAR T cell approach is the scope of further research. Our findings help to elucidate the role of B cells and provide potential targets for therapeutic intervention in patients with MASLD and HCC.

Affiliations

[1]*Department of Gastroenterology, Hepatology, Infectious Diseases and Endocrinology, Hannover Medical School (MHH), Hannover, Germany;* [2]*Department of General-, Visceral and Transplantation Surgery, MHH, Hannover, Germany;* [3]*Department of Pathological Anatomy, Forensic Medicine and Pathological Physiology, Dnipro State Medical University, Dnipro, Ukraine;* [4]*Institute of Pathology, MHH, Hannover, Germany;* [5]*Department of Vaccinology and Applied Microbiology, Helmholtz Centre for Infection Research, Braunschweig, Germany;* [6]*Department of Clinical Chemistry, MHH, Hannover, Germany*

Abbreviations

Breg, B regulatory cell; CAR T cell, chimeric antigen receptor T cell; DN, double-negative; HCC, hepatocellular carcinoma; HDI, hydrodynamic tail vein injection; HFD, high-fat diet; ICIs, immune checkpoint inhibitors; IF, immunofluorescence; IHC, immunohistochemistry; IR, inverted repeats; IRES, internal ribosome entry site; LmAIO, *Listeria monocytogenes ΔactA/ΔinlB + Ova*; MASH, metabolic dysfunction-associated steatohepatitis; MASLD, metabolic dysfunction-associated steatotic liver disease; MBCs, memory B cells; MN, mature naive; NAFLD, non-alcoholic fatty liver disease; NASH, non-alcoholic steatohepatitis; NCD, normal chow diet; NK, natural killer; NSw, non-switched; Ova, ovalbumin; pA, polyadenylation site; PBMCs, peripheral blood mononuclear cells; PBs, plasmablasts; pCaggs, synthetic CAG promoter; PCs, plasma cells; PD-1, programmed cell death protein 1; PD-L1, programmed death-ligand 1; PGK, phosphoglycerate kinase promoter; *SB13*, *Sleeping Beauty 13*; Sw, switched; TLSs, tertiary lymphoid structures; WT, wild-type.

Financial support

TY acknowledges the support of Gilead Sciences International Research Scholars Program in Liver Disease (Research Award) to TY and the German Research Foundation (DFG) under grant YE151/2-1. HB acknowledges the support of the Federal Ministry of Education and Research, LiSyM-Cancer network (031L0257H). This work was supported in part by the German Academic Exchange Service (DAAD) in the scope of the Doctoral Program in Germany 2019/2024 under grant/project-ID 91736778 to NP.

Conflicts of interest

The authors declare no competing interests.

Please refer to the accompanying ICMJE disclosure forms for further details.

Authors' contributions

Conceived the idea, designed the study, and provided the conceptual framework for the study: TY. Performed most experiments and analyzed the data: NP. Assisted during animal experiments: IH, HS. Provided human samples: KT, NJ, HB. Performed histopathological analyses: NB, LN, PK. Performed ELISA: ER, RL. Provided intellectual input and resources: TY, CAG, RL, MPM, HB. Wrote the manuscript: TY, NP. Critically reviewed and approved the final manuscript: all authors.

Data availability statement

The data that support the findings of this study are available from the corresponding authors upon reasonable request.

Acknowledgements

We are grateful to C. Petzold for excellent technical support and Prof. D. Jonigk for providing the infrastructure at the Institute of Pathology, MHH, Hannover. We also thank J. Schmidt for technical support. We are also thankful to Prof. H. Shcherbata and Dr. A. Yatsenko for providing help with the confocal laser-scanning microscopy at the Institute of Cell Biochemistry, MHH, Hannover.

Supplementary data

Supplementary data to this article can be found online at https://doi.org/10.1016/j.jhepr.2024.101189.

References

Author names in bold designate shared co-first authorship

[1] Singal AG, Lampertico P, Nahon P, et al. Epidemiology and surveillance for hepatocellular carcinoma: new trends. J Hepatol 2020;72:250–261.
[2] Llovet JM, Kelley RK, Villanueva A, et al. Hepatocellular carcinoma. Nat Rev Dis Primers 2021;7:6.
[3] Rinella ME, Lazarus JV, Ratziu V, et al. A multisociety Delphi consensus statement on new fatty liver disease nomenclature. J Hepatol 2023;79:1542–1556.
[4] Younossi ZM, Golabi P, Paik JM, et al. The global epidemiology of nonalcoholic fatty liver disease (NAFLD) and nonalcoholic steatohepatitis (NASH): a systematic review. Hepatology 2023;77:1335–1347.
[5] Finn RS, Qin S, Ikeda M, et al. Atezolizumab plus bevacizumab in unresectable hepatocellular carcinoma. New Engl J Med 2020;382:1894–1905.
[6] Sia D, Jiao Y, Martinez-Quetglas I, et al. Identification of an immune-specific class of hepatocellular carcinoma, based on molecular features. Gastroenterology 2017;153:812–826.

[7] **Montironi C**, **Castet F**, **Haber PK**, et al. Inflamed and non-inflamed classes of HCC: a revised immunogenomic classification. Gut 2023;72:129–140.
[8] Llovet JM, Castet F, Heikenwalder M, et al. Immunotherapies for hepatocellular carcinoma. Nat Rev Clin Oncol 2022;19:151–172.
[9] Michaud D, Steward CR, Mirlekar B, et al. Regulatory B cells in cancer. Immunol Rev 2021;299:74–92.
[10] Sarvaria A, Madrigal JA, Saudemont A. B cell regulation in cancer and antitumor immunity. Cell Mol Immunol 2017;14:662–674.
[11] Schneider C, Teufel A, Yevsa T, et al. Adaptive immunity suppresses formation and progression of diethylnitrosamine-induced liver cancer. Gut 2012;61:1733–1743.
[12] Largeot A, Pagano G, Gonder S, et al. The B-side of cancer immunity: the underrated tune. Cells 2019;8:449.
[13] **Hochnadel I**, **Hoenicke L**, Petriv N, et al. Safety and efficacy of prophylactic and therapeutic vaccine based on live-attenuated *Listeria monocytogenes* in hepatobiliary cancers. Oncogene 2022;41:2039–2053.
[14] Catalan D, Mansilla MA, Ferrier A, et al. Immunosuppressive mechanisms of regulatory B cells. Front Immunol 2021;12:611795.
[15] Rosser EC, Mauri C. Regulatory B cells: origin, phenotype, and function. Immunity 2015;42:607–612.
[16] Barrow F, Khan S, Wang H, et al. The emerging role of B cells in the pathogenesis of NAFLD. Hepatology 2021;74:2277–2286.
[17] **Yanaba K**, **Bouaziz JD**, Haas KM, et al. A regulatory B cell subset with a unique CD1dhiCD5+ phenotype controls T cell-dependent inflammatory responses. Immunity 2008;28:639–650.
[18] Shalapour S, Lin XJ, Bastian IN, et al. Inflammation-induced IgA+ cells dismantle anti-liver cancer immunity. Nature 2017;551:340–345.
[19] **Kotsiliti E**, **Leone V**, Schuehle S, et al. Intestinal B cells license metabolic T-cell activation in NASH microbiota/antigen-independently and contribute to fibrosis by IgA-FcR signalling. J Hepatol 2023;79:296–313.
[20] Kaminski DA, Wei C, Qian Y, et al. Advances in human B cell phenotypic profiling. Front Immunol 2012;3:302.
[21] **Weisel NM**, **Joachim SM**, Smita S, et al. Surface phenotypes of naive and memory B cells in mouse and human tissues. Nat Immunol 2022;23:135–145.
[22] Grimsholm O. CD27 on human memory B cells-more than just a surface marker. Clin Exp Immunol 2023;213:164–172.
[23] **Tang B**, **Zhu J**, Li J, et al. The ferroptosis and iron-metabolism signature robustly predicts clinical diagnosis, prognosis and immune microenvironment for hepatocellular carcinoma. Cell Commun Signal 2020;18:174.
[24] **Wang XD**, **Wang L**, Ji FJ, et al. Decreased CD27 on B lymphocytes in patients with primary hepatocellular carcinoma. J Int Med Res 2012;40:307–316.
[25] **Kang TW**, **Yevsa T**, Woller N, et al. Senescence surveillance of pre-malignant hepatocytes limits liver cancer development. Nature 2011;479:547–551.
[26] Eggert T, Wolter K, Ji J, et al. Distinct functions of senescence-associated immune responses in liver tumor surveillance and tumor progression. Cancer Cell 2016;30:533–547.
[27] Dauch D, Rudalska R, Cossa G, et al. A MYC-aurora kinase A protein complex represents an actionable drug target in p53-altered liver cancer. Nat Med 2016;22:744–753.
[28] **Petriv N**, **Neubert L**, Vatashchuk M, et al. Increase of alpha-dicarbonyls in liver and receptor for advanced glycation end products on immune cells are linked to nonalcoholic fatty liver disease and liver cancer. Oncoimmunology 2021;10:1874159.
[29] Zheng K, Cubero FJ, Nevzorova YA. c-MYC-making liver sick: role of c-MYC in hepatic cell function, homeostasis and disease. Genes 2017;8:123.
[30] Moser B, Loetscher P. Lymphocyte traffic control by chemokines. Nat Immunol 2001;2:123–128.
[31] Stein JV, Nombela-Arrieta C. Chemokine control of lymphocyte trafficking: a general overview. Immunology 2005;116:1–12.
[32] Kitamura D, Roes J, Kuhn R, et al. A B cell-deficient mouse by targeted disruption of the membrane exon of the immunoglobulin mu chain gene. Nature 1991;350:423–426.
[33] Gu H, Zou YR, Rajewsky K. Independent control of immunoglobulin switch recombination at individual switch regions evidenced through Cre-loxP-mediated gene targeting. Cell 1993;73:1155–1164.
[34] Bach AG. TNM classification of malignant tumours. Radiologe 2017;57:244–245.
[35] Wen S, Chen Y, Hu C, et al. Combination of tertiary lymphoid structure and neutrophil-to-lymphocyte ratio predicts survival in patients with hepatocellular carcinoma. Front Immunol 2021;12:788640.
[36] Horii M, Matsushita T. Regulatory B cells and T cell regulation in cancer. J Mol Biol 2021;433:166685.
[37] Pylayeva-Gupta Y, Das S, Handler JS, et al. IL35-producing B cells promote the development of pancreatic neoplasia. Cancer Discov 2016;6:247–255.
[38] **Iwai Y**, **Ishida M**, Tanaka Y, et al. Involvement of PD-L1 on tumor cells in the escape from host immune system and tumor immunotherapy by PD-L1 blockade. Proc Natl Acad Sci U S A 2002;99:12293–12297.
[39] Dong H, Strome SE, Salomao DR, et al. Tumor-associated B7-H1 promotes T-cell apoptosis: a potential mechanism of immune evasion. Nat Med 2002;8:793–800.
[40] Nomi T, Sho M, Akahori T, et al. Clinical significance and therapeutic potential of the programmed death-1 ligand/programmed death-1 pathway in human pancreatic cancer. Clin Cancer Res 2007;13:2151–2157.
[41] Biswas S, Mandal G, Payne KK, et al. IgA transcytosis and antigen recognition govern ovarian cancer immunity. Nature 2021;591:464–470.
[42] **Shimabukuro-Vornhagen A**, **Schlosser HA**, Gryschok L, et al. Characterization of tumor-associated B-cell subsets in patients with colorectal cancer. Oncotarget 2014;5:4651–4664.
[43] Mohammed ZMA, Going JJ, Edwards J, et al. The relationship between lymphocyte subsets and clinico-pathological determinants of survival in patients with primary operable invasive ductal breast cancer. Br J Cancer 2013;109:1676–1684.
[44] **Lino AC**, **Dang VD**, Lampropoulou V, et al. LAG-3 inhibitory receptor expression identifies immunosuppressive natural regulatory plasma cells. Immunity 2018;49:120–133.e9.
[45] Shao W, Zhang C, Liu E, et al. Identification of liver epithelial cell-derived Ig expression in mu chain-deficient mice. Sci Rep 2016;6:23669.
[46] Macpherson AJ, Lamarre A, McCoy K, et al. IgA production without mu or delta chain expression in developing B cells. Nat Immunol 2001;2:625–631.
[47] **Tan P**, **Shi M**, **Lai L**, et al. Regulative role of the CXCL13-CXCR5 axis in the tumor microenvironment. Precis Clin Med 2018;1:49–56.
[48] Gao SH, Liu SZ, Wang GZ, et al. CXCL13 in cancer and other diseases: biological functions, clinical significance, and therapeutic opportunities. Life 2021;11:1282.
[49] Gu-Trantien C, Migliori E, Buisseret L, et al. CXCL13-producing TFH cells link immune suppression and adaptive memory in human breast cancer. JCI Insight 2017;2:e91487.
[50] Jogalekar MP, Rajendran RL, Khan F, et al. CAR T-cell-based gene therapy for cancers: new perspectives, challenges, and clinical developments. Front Immunol 2022;13:925985.

Keywords: Metabolic dysfunction-associated steatotic liver disease; Non-alcoholic fatty liver disease; Hepatocellular carcinoma; B cells; B regulatory cells; Memory B cells; plasmablasts.
Received 14 November 2023; received in revised form 5 August 2024; accepted 10 August 2024; Available online 22 August 2024

Journal of Hepatology, Volume *6*

Supplemental information

Essential roles of B cell subsets in the progression of MASLD and HCC

Nataliia Petriv, Huizhen Suo, Inga Hochnadel, Kai Timrott, Nina Bondarenko, Lavinia Neubert, Elena Reinhard, Nils Jedicke, Patrick Kaufhold, Carlos Alberto Guzmán, Ralf Lichtinghagen, Michael P. Manns, Heike Bantel, and Tetyana Yevsa

Essential roles of B cell subsets in the progression of MASLD and HCC

Nataliia Petriv, Huizhen Suo, Inga Hochnadel, Kai Timrott, Nina Bondarenko, Lavinia Neubert, Elena Reinhard, Nils Jedicke, Patrick Kaufhold, Carlos Alberto Guzmán, Ralf Lichtinghagen, Michael P. Manns, Heike Bantel, Tetyana Yevsa

Table of contents

Abbreviations……………………………......…………………………………………..……...…2

Supplementary materials and methods……………………………......……………..…………3

Supplementary figures…………………………………………………………….....……..…8

Graphical abstract legend …….………….....……………………………………….….…..…...20

Supplementary tables………………………………………………………….…..............21

Supplementary references…………………………………………………………..…...27

Abbreviations

AFP	alpha-1-fetoprotein
BMI	body mass index
Breg	B regulatory cell
BSA	bovine serum albumin
DAPI	4’,6-diamidino-2-phenylindole
DN	double-negative
EDTA	ethylenediaminetetraacetic acid
FBS	fetal bovine serum
GGT	gamma-glutamyltransferase
H&E	hematoxylin and eosin
HCC	hepatocellular carcinoma
HDI	hydrodynamic tail vein injection
HFD	high-fat diet
HRP	horseradish peroxidase
IF	immunofluorescence
IHC	immunohistochemistry
IRES	internal ribosome entry site
LmAIO	*Listeria monocytogenes ∆actA/∆inlB + Ova*
MBCs	memory B cells
MN	mature naïve
MASLD	metabolic dysfunction-associated steatotic liver disease
NCD	normal chow diet
NSw	non-switched
Ova	ovalbumin
PBS	phosphate buffered saline
PBs	plasmablasts
PD-L1	programmed death-ligand 1
SB13	*Sleeping Beauty 13*
Sw MBCs	switched memory B cells

Supplementary materials and methods

Human samples

Fresh blood samples, obtained from the patients with metabolic dysfunction-associated steatotic liver disease (MASLD), were prepared for FACS analysis. Liver tissue samples (resected material after surgeries) were collected only from the patients with hepatocellular carcinoma (HCC). The tumor liver tissues were harvested for immunohistochemical (IHC) and immunofluorescent (IF) analyses. The prospective study using all human material (blood and liver tissues obtained after surgeries) was conducted in accordance with the Helsinki Declaration. It was approved by the Ethics Committee of the MHH (approval numbers: 3261_BO_K_2016, 7825_BO_K_2018 and 8742_BO_K_2019). The appropriate informed consent was received from all the patients.

Mouse strains, animal housing and diets used

Wild-type (WT) C57BL/6J mice were purchased from Charles River Laboratories. *P19*$^{Arf-/-}$ mice [1] were obtained in a C57BL/6J background as described previously [2, 3]. B-cell-deficient mice (B6.129S2-Igh-6$^{tm1Cgn/J}$ (µMT) [4] and B6.129P2-Igh-J$^{tm1Cgn/J}$ (JHT) [5]) were purchased from the Jackson Laboratory and were bred at the animal facility of the MHH, Hannover, Germany.

Mice were maintained on a 12 h light and dark cycle with *ad libitum* access to water and a normal chow diet composed of 10% fat. For inducing MASLD, three-weeks-old C57BL/6J female mice were fed with a 60% high-fat diet (HFD, Envigo Teklad TD06414) for 14 weeks long, as previously established [6-8].

B-cell depletion and combination treatment with α-CD20

B-cell depletion was carried out using a monoclonal α-CD20 antibody from BioLegend (clone SA271G2). Mice were treated with 250 µg α-CD20 antibody administered intraperitoneally (i.p.) twice per week [9, 10]. The combination therapy, comprising the α-CD20 treatment and a vaccination with a double-deleted vaccine strain *Listeria monocytogenes ΔactA/ΔinlB*, expressing ovalbumin (designated LmAIO), was performed in HCC-Ova mouse model induced in *p19*$^{Arf-/-}$ mice, as described previously [11]. All antibodies used in the study are listed in Table S3.

Isolation of human peripheral blood mononuclear cells (PBMCs)

Peripheral human blood was obtained by venepuncture and collected into tubes containing ethylenediaminetetraacetic acid (EDTA) as an anticoagulant. PBMCs were obtained using Ficoll-Paque PLUS (GE Healthcare, USA) density gradient centrifugation, according to the

manufacturer's instructions. Briefly, whole blood was diluted with an equal volume of PBS (at room temperature), underlain with Ficoll-Paque (at room temperature), then centrifuged (400×*g*, 30 min, 21 °C) on a Heraeus Megafuge 40R (Thermo Fisher Scientific, USA) without break. PBMCs were collected from the Ficoll-Paque-plasma interface into a 50 ml tube (Greiner Bio-One, Germany), washed twice in PBS (100×*g*, 10 min, 4 °C), and re-suspended at 10^6 cells/ml in complete RPMI 1640 (Gibco, USA). Then, the total number of cells was counted using 0.4% trypan blue (Sigma Aldrich, USA) and a cell counter (LUNA-FL, Logos Biosystems, South Korea).

Vector design and induction of HCC using hydrodynamic tail vein injection (HDI)

Sleeping Beauty 13 (*SB13*) transposase, *NRAS*^{G12V} (*CaN*), and *Myc-NRAS*^{G12V}*-IRES* (*CaMIN*) encoding transposon vectors have been described recently [2, 8, 12]. DNA vectors (transposon and transposase) for HDI were prepared using the QIAGEN EndoFreeMaxi Kit (QIAGEN, Hilden, Germany). For transposon-mediated gene transfer, 4- to 6-week-old animals received a 25 µg:5 µg ratio of transposon to transposase-encoding plasmid, as previously described [2, 3, 13]. DNA was diluted in saline solution at a final volume of 10% of body weight. The model of transposon-mediated stable intrahepatic transfer of oncogenic *NRAS*^{G12V} *or Myc-IRES-NRAS*^{G12V} were used to induce HCC development in *p19*$^{Arf-/-}$ or C57BL/6J mice respectively [1-3, 8]. HCC-Ova was induced in *p19*$^{Arf-/-}$ mice, as previously described [11].

Leukocyte isolation from murine liver tissues and blood

Single-cell suspensions from the murine livers were prepared as previously described [3, 8, 13]. Briefly, liver tissue was chopped into small ~1 mm^3 pieces and then enzymatically digested in complete DMEM medium (Gibco, USA) supplemented with 5% fetal bovine serum (FBS, Roche, Switzerland), 0.5 mg/ml collagenase D (Roche, Switzerland) and 0.01 mg/ml DNAse I (Sigma Aldrich, USA) for 25 min at 37 °C. After incubation, the enzymatic reaction was stopped using 5 mM EDTA and, the liver suspension was passed through a 100 µm cell strainer. Erythrocytes were lysed using an ACK buffer (150 mM NH_4Cl, 10 mM $KHCO_3$, and 0.1 mM EDTA) [3].

Blood was collected from the retro-orbital plexus and mixed with 50 units of heparin (Ratiopharm GmbH, Germany) and erythrocytes were lysed using ACK buffer. The obtained single-cell suspensions were used for stainings and multicolor flow cytometry (FACS) analysis.

Cell suspension staining and multicolor FACS analysis

Isolated single-cell suspensions were stained in 96-well U-bottom plates with fluorochrome-conjugated monoclonal antibodies in FACS buffer (PBS supplemented with 2% FBS, pH=7.4). To avoid Fc-mediated nonspecific interactions, murine cells were pre-incubated with the anti-CD16/32 (clone 93, BioLegend, USA), and human cells were incubated with FcR blocking reagent (BioLegend, USA). Dead cells were excluded based on staining with the Alexa Fluor 350 NHS Ester (Life Technologies, USA). For staining of murine samples, the following monoclonal antibodies were used: anti-B220 (clone RA3-6B2), anti-CD5 (clone 53-7.3), anti-CD1d (clone 1B1), anti-PD-L1 (clone 10F.9G2), anti-IgM (clone RMM-1), anti-CD1d (clone 1B1), anti-IgD (clone 11-26c.2a), anti-CD27 (clone LG.3A10), anti-CD138 (clone 281-2), anti-CD267 (clone 8F10), anti-IL-10 (clone JES5-16E3), anti-CD19 (clone 1D3), anti-IgA (clone 11-44-2).

The antibodies used for the human PBMC staining were the following: anti-CD3 (clone SK7), anti-CD11c (clone 3.9), anti-CD19 (clone HIB19), anti-CD20 (clone 2H7), anti-CD5 (clone L17F12), anti-CD1d (clone 51.1), anti-CD27 (clone M-T271), anti-IgM (clone MHM-88), anti-PD-L1 (clone 29E.2A3), anti-IL-10 (clone JES3-9D7), anti-CD45 (clone HI30), anti-IgD (clone IA6-2). All further information about the antibodies, including the corresponding purchasers, are listed in Table S3.

Samples were acquired on LSR II flow cytometer (BD Biosciences, USA) using BD FACS Diva software (Becton Dickinson Ltd, Oxford, UK) and/or a Cytek Aurora flow cytometer using a SpectroFlo software (Cytek, USA) and analyzed using a FlowJo software (Tree Star, Becton, Dickinson & Company, USA).

Histopathological examination

Murine and resected human liver tissue samples were fixed in 4% paraformaldehyde at room temperature for 24-48 hours, embedded in paraffin, and serially sectioned at 4 μm using a rotary microtome (Microm, Germany). The obtained tissue sections were stained with H&E (Sigma Aldrich, USA and Merck, Germany), argentum (Honeywell-Fluka, USA), and Sirius red (Sigma Aldrich, USA) for the histopathological evaluation, as described [14, 15].

For detection of lipid accumulation in hepatocytes, snap-frozen murine liver tissue samples were sectioned at 6 μm and stained with Oil Red O (Sigma Aldrich, USA), following the routine standard operating procedures at the Institute of Pathology at the MHH [16]. For quantitative morphometry of positive oil area (%, Oil Red O) and collagen deposition (%, Sirius red) representative images of at least five random fields (100x magnification) were calculated using the ImageJ software.

The immune infiltrates were evaluated at the entire liver tissue on the slide for the presence of lymphocytic immune cells that were scored following the recommendations of Sia D. *et al*

using the scoring from 0 to 4 as follows: 0 (absence of immune cell infiltration), 1 (minimal infiltration), 2 (mild infiltration), 3 (moderate infiltration), and 4 (strong infiltration) [17, 18]. In the present study, we characterized samples as the 'non-inflamed' HCC if the sample's score was between 0 to 2 (minimal to mild immune cell infiltration) and the 'inflamed' HCC, if the immune infiltrate score reached levels 3 and 4 (moderate to strong immune cell infiltration).
Liver specimens were analyzed using a standard bright field microscopy (BX51, Olympus (Olympus, Japan) and a Nikon Eclipse Ti2 inverted microscope (Nikon, Japan)). The histopathological analyses were confirmed by two experienced pathologists.

Immunohistochemistry (IHC) analysis

IHC staining was performed on 4 μm-thick formalin-fixed paraffin-embedded tissue sections after heat-induced antigen retrieval in a decloaking chamber (Water Bath 1008, GFL) using citrate buffer (10 mM sodium citrate and 0.05% Tween 20, pH=6.0). Incubation with the primary antibodies to anti-CD19 (1:750, Invitrogen, USA), anti-CD5 (RTU, Epredia, USA), anti-CD1d (1:1200, antibodies-online, Germany), anti-IgD (1:1000, Abcam, USA), anti-PD-L1 (1:800, Invitrogen, USA), anti-IL-10 (1:500, Invitrogen, USA) was carried out in 5% bovine serum albumin (BSA, Carl Roth, USA) at room temperature for 1 h. Detailed information about all antibodies used for the IHC staining is provided in Table S3. Signal amplification and colour development via diaminobenzidine were performed using Epredia™ UltraVision™ Quanto Detection System horseradish peroxidase (HRP) DAB (Fisher Scientific, USA) and a counter-staining with Mayer's hematoxylin (Carl Roth, USA).
Images were analyzed using the Nikon Eclipse Ti2 inverted microscope (Nikon, Japan). The quantification of CD19, CD5, CD1d, IgD, PD-L1, and IL-10 positive immune cells was performed using at least five representative fields (100X) and presented as a cellular density (number of positive cells per 1 mm^2).

Immunofluorescent (IF) staining of the frozen liver tissue sections

Frozen 7 μm-thick liver tissue sections were fixed in ice-cold acetone for 10 min and permeabilized using the 0.1% Triton X-100 (Sigma Aldrich, USA) and 0.1% sodium citrate dihydrate (Fisher Scientific, USA) for 2 minutes at 4°C. Sections were blocked with a PBTB buffer (PBS supplemented with 0.2% Triton X-100 (PBT), 0.2% BSA (Carl Roth, USA), 5% normal goat serum (Abcam, USA) for 30 min at room temperature and incubated, as previously described [8]. Human liver sections were stained with a mix of primary antibodies for 30 min at room temperature: anti-CD19 (clone 6OMP31, Invitrogen, USA), anti-CD5 (clone UCH-T2, Santa Cruz, USA), anti-CD1d (polyclonal, antibodies-online, Germany), anti-IgD (clone EPR6146, Abcam, USA), anti-IgM (clone R1/69, Santa Cruz, USA), anti-IL-10

(polyclonal, Abcam, USA), anti-PD-L1 (polyclonal, Invitrogen, USA). All information about the antibodies, including the corresponding purchasers, are listed in Table S3.

The secondary, fluorescent-conjugated antibodies: Alexa Fluor 488-conjugated donkey anti-rat, Alexa Fluor 555-conjugated donkey anti-rabbit, and Alexa Fluor 647-conjugated donkey anti-mouse antibody (all used at 1:400 dilution, Invitrogen) were applied for 1 h at room temperature. Thereafter, sections were counterstained with 4',6-diamidino-2-phenylindole (DAPI, 0.1 µg/ml, Sigma-Aldrich) in PBS for 10 min at room temperature. Finally, samples were mounted using Citifluor AF1 mountant solution (Electron Microscopy Sciences, Hatfield, PA, USA). Fluorescent images were analyzed using a Zeiss LSM 700 confocal laser-scanning microscope (Carl Zeiss, Germany) (40x objective), ZEN 2011 software and ImageJ software.

ELISA

IgG, IgA, IgM, and IgD levels of antibodies were analyzed in the plasma of mice according to standard ELISA protocols described previously [11]. Briefly, flat-bottomed 96-well ELISA plates were coated overnight with the corresponding antigen dissolved in carbonate buffer. Thereafter, plates were blocked with 3% BSA/PBS and supplied afterwards with murine plasma for 1 hour at 37°C. Detection was performed using anti-mouse IgM-biotin (Sigma Aldrich, USA), anti-mouse IgG-biotin (Sigma Aldrich, USA), or anti-mouse IgA-biotin (Southern Biotech, USA) followed by the incubation with streptavidin-conjugated HRP (BD Pharmingen, USA). HRP substrate solution 2,2'-azino-bis (3-ethylbenzothiazoline-6-sulphonic acid, Sigma Aldrich, USA) was added for the detection of bound IgG/IgM/IgA. Mouse IgD was detected using a sandwich ELISA Kit (CusaBio, USA). Samples were measured at the optical density of 405 nm (OD_{405}) using Synergy 2 microplate reader (BioTek, Winooski, Vermont, USA). All samples were examined in triplicates. Software Gen5 was used for the data analysis. All reagents used for ELISA analyses are listed in Table S3.

Statistical analysis

Graph design and statistical analyses were performed using GraphPad Prism 8.3 software (GraphPad Prism, San Diego, USA). The normality of the calculated variables was assessed by using the Kolmogorov–Smirnov test. To compare means, the nonparametric Mann–Whitney *U*-test was used for non-normally distributed data, whereas the unpaired Student's *t* test was applied for normally distributed data. The relationship of variables was analyzed by the unpaired Student's *t* test and Mann–Whitney nonparametric test. If not stated otherwise, the data are shown as mean +/- standard error of the mean (SEM). *P* values < 0.05 were considered statistically significant (*$p \leq 0.05$, **$p \leq 0.01$, ***$p \leq 0.001$, and ****$p \leq 0.001$).

Supplementary figures

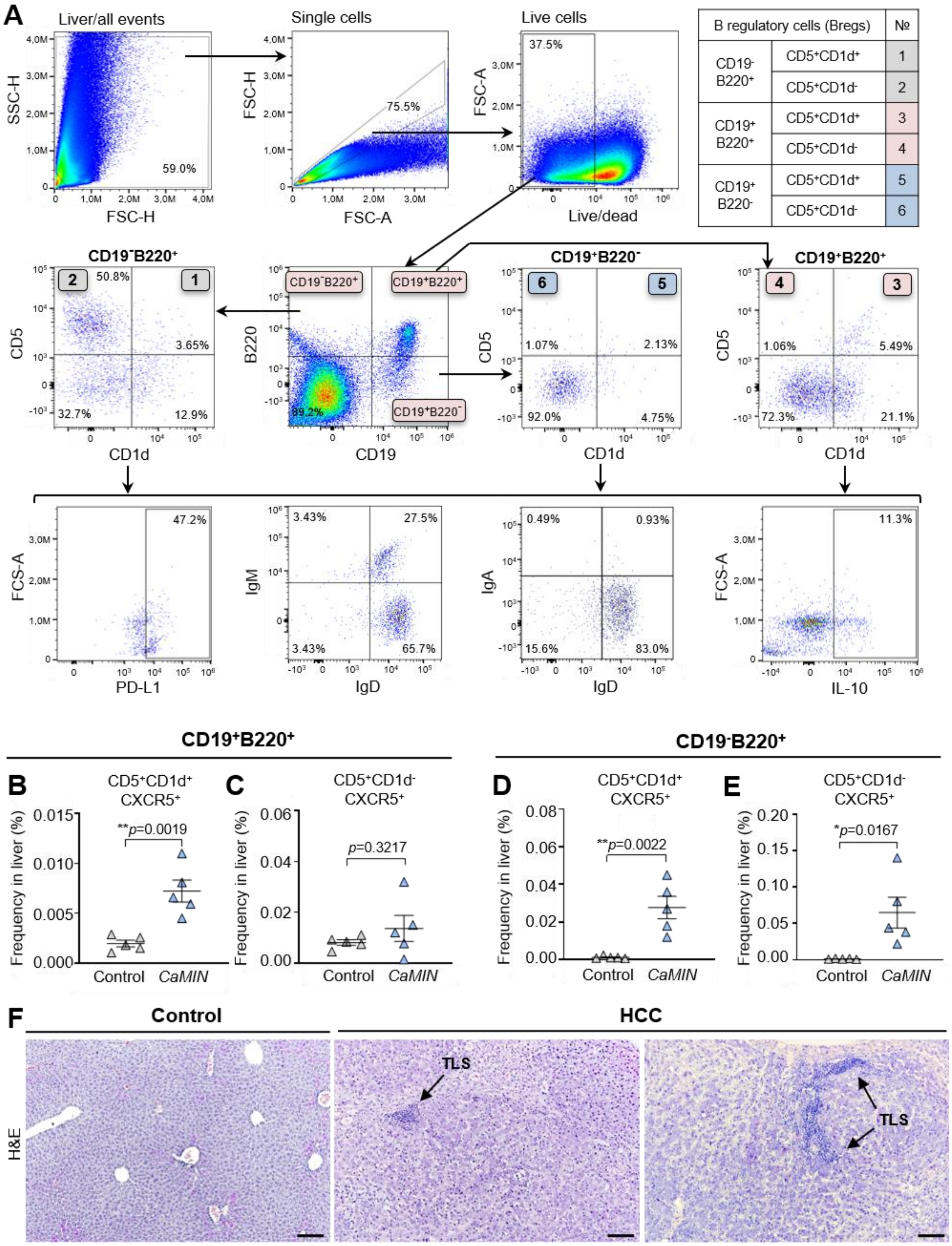

Fig. S1. Gating strategy to phenotype and characterize Bregs; elevated numbers of CXCR5+ Bregs and presence of TLS structures in murine HCC. (A) Gating strategy to define and characterize various populations of Bregs. Single-cell suspensions were obtained from the livers and blood of mice with MASLD, HCC/*NRAS*^{G12V}/*p19*$^{Arf-/-}$, and HCC/*CaMIN*.

The cell suspensions were stained and analyzed via the indicated surface and intracellular markers using multicolor FACS. Based on CD19, CD20, CD5, and CD1d markers six distinct populations of Bregs were identified. Further, the immunomodulatory properties of Bregs were assessed while checking PD-L1 and IL-10 expression. In addition, based on IgM, IgD, and IgA expression, several subpopulations of Bregs were defined. **(B-E)** Bregs were further checked for the presence of the homing receptor $CXCR5^+$ in the livers of HCC/*CaMIN* mice: frequencies of $CXCR5^+$-expressing **(B)** $CD19^+B220^+CD5^+CD1d^+$, **(C)** $CD19^+B220^+CD5^+CD1d^-$, **(D)** $CD19^-B220^+CD5^+CD1d^+$ and **(E)** $CD19^-B220^+CD5^+CD1d^-$ Bregs. **(F)** TLS assessment in H&E-stained murine HCC liver (magnification 100x; scale bar, 100 µm). The data were analyzed using the unpaired Student's *t* test. The data are shown as the mean ± SEM, n = 5-6. $*p < 0.05$, $**p < 0.01$. Bregs – B regulatory cells, MASLD – metabolic dysfunction-associated steatotic liver disease, TLS – tertiary lymphoid structures.

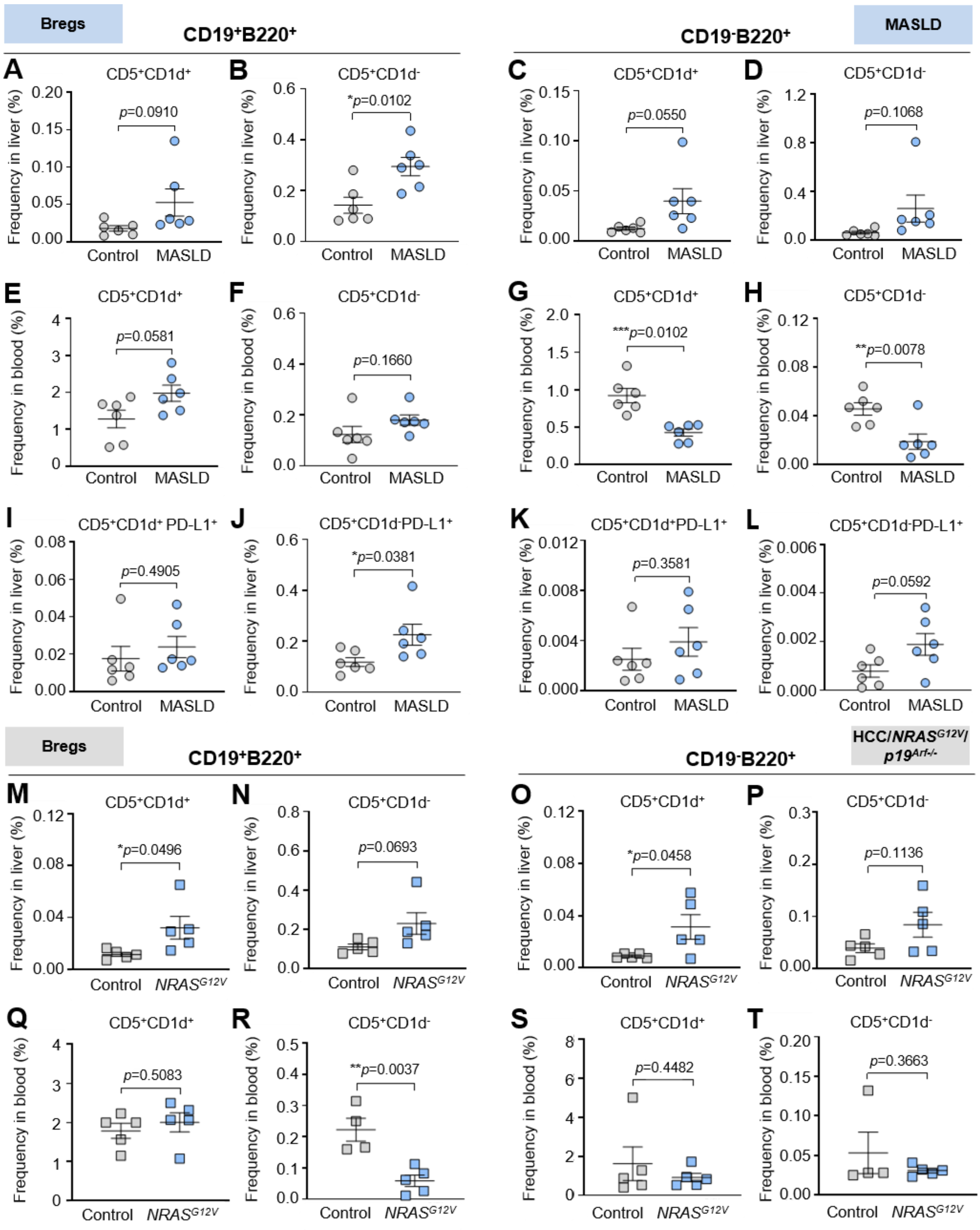

Fig. S2. Bregs phenotype in MASLD and HCC/*NRAS*^{G12V}/*p19*$^{Arf-/-}$ mouse models. (A, B) Frequencies of **(A)** CD19$^+$B220$^+$CD5$^+$CD1d$^+$ and **(B)** CD19$^+$B220$^+$CD5$^+$CD1d$^-$ B cells in the liver of MASLD mice. **(C, D)** Frequencies of **(C)** CD19$^-$B220$^+$CD5$^+$CD1d$^+$ and **(D)** CD19$^-$B220$^+$CD5$^+$CD1d$^-$ B cells in the liver of MASLD mice. **(E, H)** Frequencies of **(E)** CD19$^+$B220$^+$CD5$^+$CD1d$^+$ and **(F)** CD19$^+$B220$^+$CD5$^+$CD1d$^-$ B cells in the blood of MASLD mice. **(G, H)** Frequencies of **(G)** CD19$^-$B220$^+$CD5$^+$CD1d$^+$ and **(H)** CD19$^-$B220$^+$CD5$^+$CD1d$^-$ B cells in the blood of MASLD mice. **(I-L)** Frequencies of PD-L1$^+$-expressing Bregs in the livers

of MASLD mice. **(M, N)** Frequencies of **(M)** $CD19^{+}B220^{+}CD5^{+}CD1d^{+}$ and **(N)** $CD19^{+}B220^{+}CD5^{+}CD1d^{-}$ B cells in the liver of HCC/*NRAS*^{G12V}/*p19*$^{Arf-/-}$ mice. **(O, P)** Frequencies of **(O)** $CD19^{-}B220^{+}CD5^{+}CD1d^{+}$ and **(P)** $CD19^{-}B220^{+}CD5^{+}CD1d^{-}$ B cells in the liver of HCC/*NRAS*^{G12V}/*p19*$^{Arf-/-}$ mice. **(Q, R)** Frequencies of **(Q)** $CD19^{+}B220^{+}CD5^{+}CD1d^{+}$ and **(R)** $CD19^{+}B220^{+}CD5^{+}CD1d^{-}$ B cells in the blood of HCC/*NRAS*^{G12V}/*p19*$^{Arf-/-}$ mice. **(S, T)** Frequencies of **(S)** $CD19^{-}B220^{+}CD5^{+}CD1d^{+}$ and **(T)** $CD19^{-}B220^{+}CD5^{+}CD1d^{-}$ B cells in the blood of HCC/*NRAS*^{G12V}/*p19*$^{Arf-/-}$ mice. The data were analyzed using the unpaired Student's *t* test. The data are shown as the mean ± SEM, n = 5-6. *$p < 0.05$, **$p < 0.01$, ***$p < 0.001$. Bregs – B regulatory cells, MASLD – metabolic dysfunction-associated steatotic liver disease.

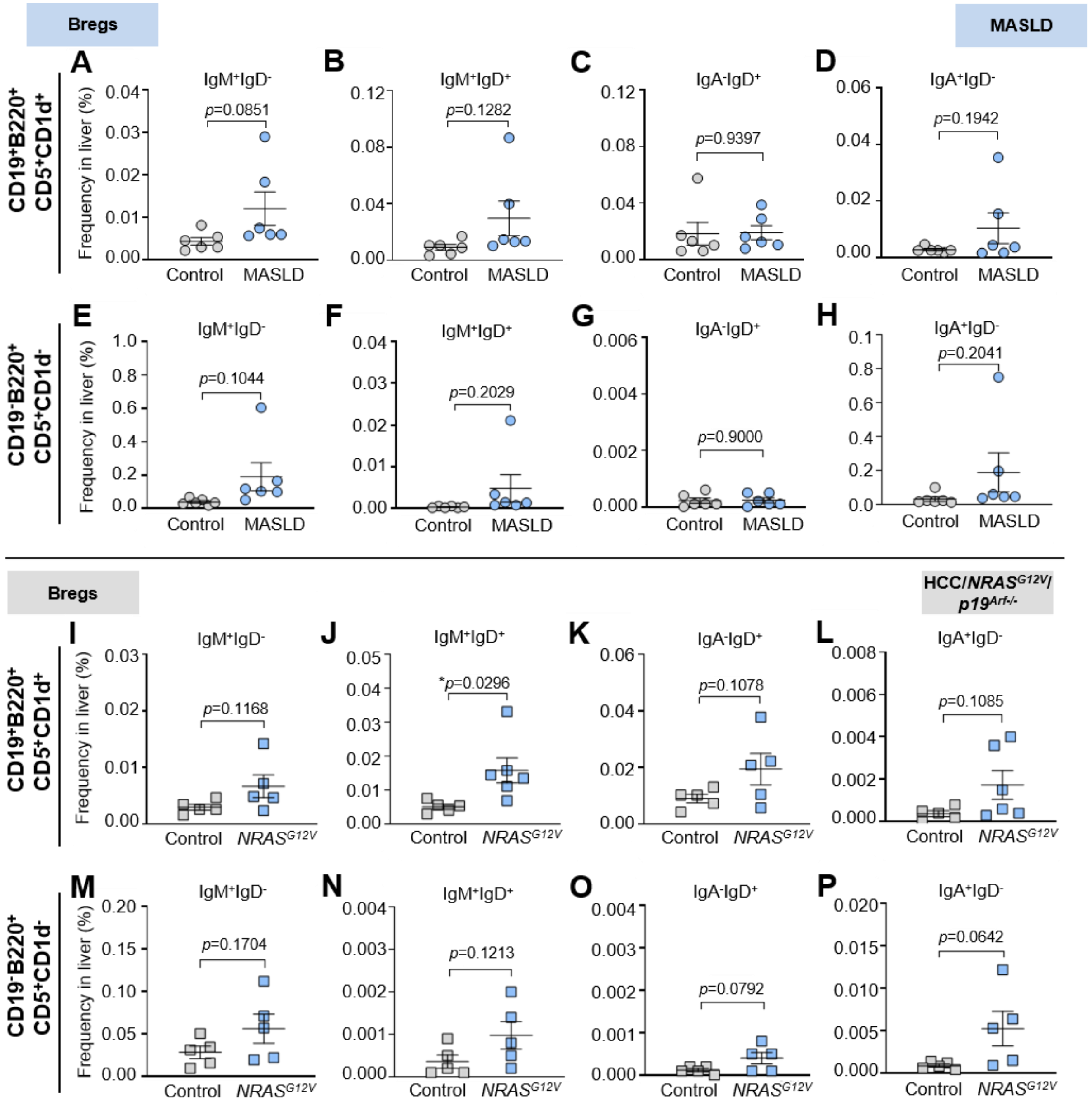

Fig. S3. IgM$^+$, IgD$^+$, and IgA$^+$ Bregs in the livers of MASLD and HCC/*NRAS*^{G12V}/*p19*$^{Arf-/-}$ mice. (A-H), Frequencies of **(A, E)**, IgM$^+$IgD$^-$-, **(B, F)** IgM$^+$IgD$^+$-, **(C, G)** IgA$^-$IgD$^+$-, and **(D, H)** IgA$^+$IgD$^-$-expressing CD19$^+$B220$^+$CD5$^+$CD1d$^+$ and CD19$^-$B220$^+$CD5$^+$CD1d$^-$ Bregs, respectively, in the livers of MASLD mice. **(I-P)** Frequencies of **(I, M)** IgM$^+$IgD$^-$-, **(J, N)** IgM$^+$IgD$^+$-, **(K, O)** IgA$^-$IgD$^+$-, and **(L, P)** IgA$^+$IgD$^-$-expressing CD19$^+$B220$^+$CD5$^+$CD1d$^+$ and CD19$^-$B220$^+$CD5$^+$CD1d$^-$ Bregs, respectively, in the livers of HCC/*NRAS*^{G12V}/*p19*$^{Arf-/-}$ mice. The data were analyzed using the unpaired Student's *t* test. The data are shown as the mean ± SEM, n = 5-6. *$p < 0.05$. Bregs – B regulatory cells, MASLD – metabolic dysfunction-associated steatotic liver disease.

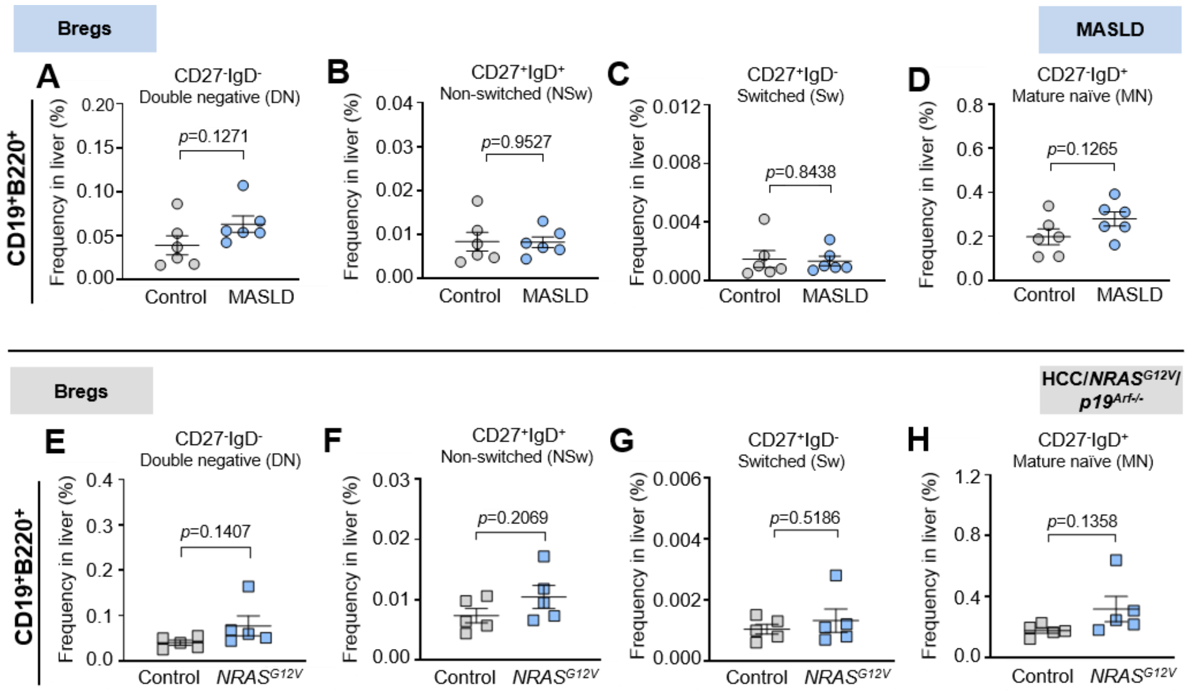

Fig. S4. MBCs in the livers of MASLD and HCC/*NRAS*^{G12V}/*p19*$^{Arf-/-}$ mice. (A-D) Frequencies of **(A)** CD27$^-$IgD$^-$ DN, **(B)** CD27$^+$IgD$^+$ NSw, **(C)** CD27$^+$IgD$^-$ Sw, and **(D)** CD27$^-$IgD$^+$ MN MBCs in the livers and blood of MASLD mice. **(E-H)** Frequencies of **(E)** CD27$^-$IgD$^-$ DN, **(F)** CD27$^+$IgD$^+$ NSw, **(G)** CD27$^+$IgD$^-$ Sw, and **(H)** CD27$^-$IgD$^+$ MN MBCs in the livers of HCC/*NRAS*^{G12V}/*p19*$^{Arf-/-}$ mice. The data were analyzed using the unpaired Student's *t* test. The data are shown as the mean ± SEM, n = 5-6. MASLD – metabolic dysfunction-associated steatotic liver disease, MBCs – memory B cells, DN – double negative, NSw – non-switched, Sw – switched, MN – mature naïve.

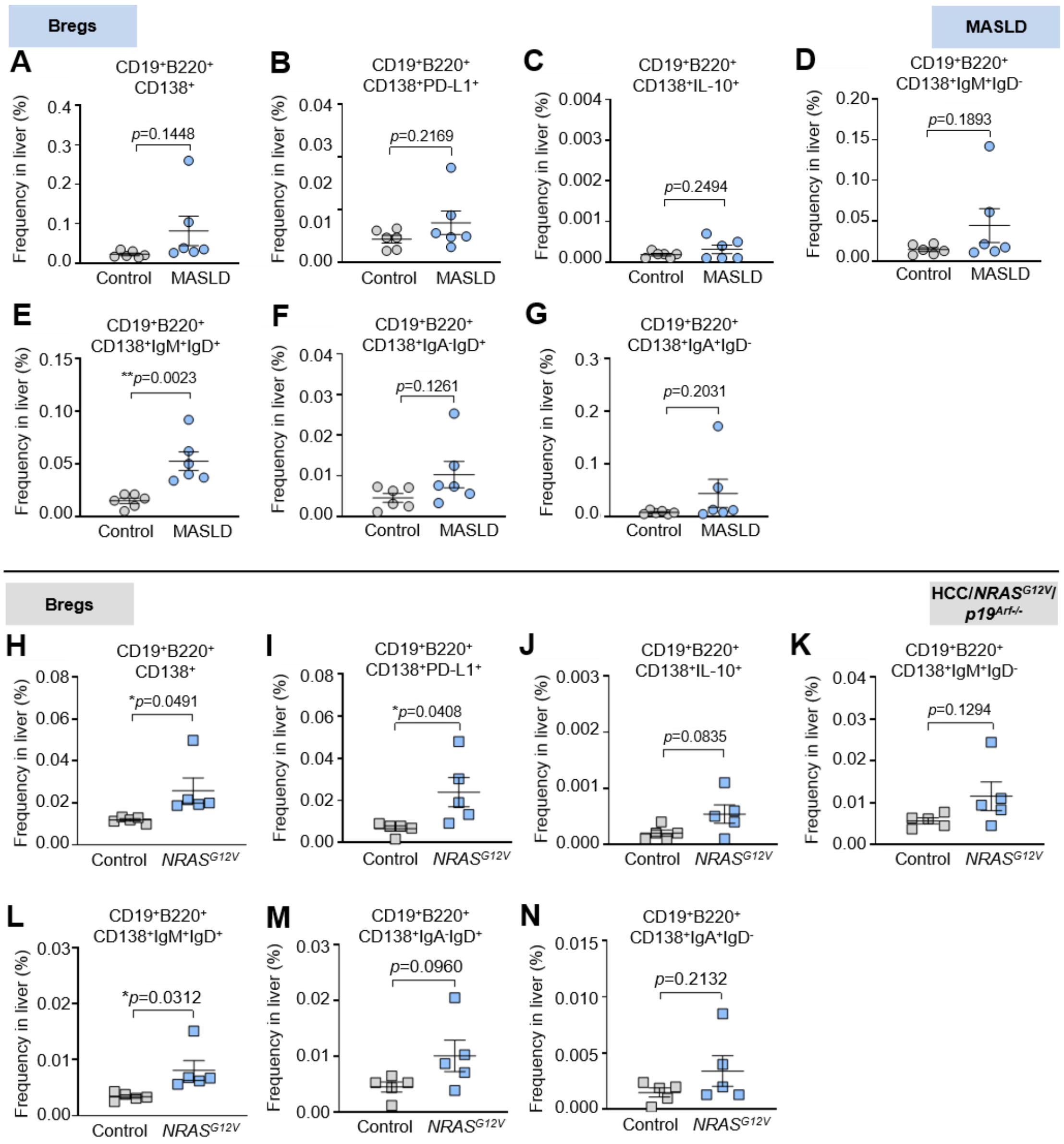

Fig. S5. Elevated frequencies of PD-L1$^+$- and IgM$^+$IgD$^+$-expressing CD19$^+$B220$^+$CD138$^+$ PBs in the livers of MASLD and HCC/*NRAS*^{G12V}/*p19*$^{Arf-/-}$ mice. (A-C) Frequencies of **(A)** CD19$^+$B220$^+$CD138$^+$, **(B)** CD19$^+$B220$^+$CD138$^+$PD-L1$^+$, and **(C)** CD19$^+$B220$^+$CD138$^+$IL-10$^+$ B cells in the livers of MASLD mice. **(D-G)** Frequencies of **(D)** IgM$^+$IgD$^-$, **(E)** IgM$^+$IgD$^+$, **(F)**, IgA$^-$IgD$^+$, and **(G)** IgA$^+$IgD$^-$ CD19$^+$B220$^+$CD138$^+$ B cells in the livers of MASLD mice. **(H-F)** Frequencies of **(H)** CD19$^+$B220$^+$CD138$^+$, **(I)** CD19$^+$B220$^+$CD138$^+$PD-L1$^+$, and **(J)** CD19$^+$B220$^+$CD138$^+$IL-10$^+$ B cells in the livers of HCC/*NRAS*^{G12V}/*p19*$^{Arf-/-}$ mice. **(K-N)** Frequencies of **(K)** IgM$^+$IgD$^-$, **(L)** IgM$^+$IgD$^+$, **(M)**, IgA$^-$IgD$^+$, and **(N)** IgA$^+$IgD$^-$ CD19$^+$B220$^+$CD138$^+$ B cells in the livers of HCC/*NRAS*^{G12V}/*p19*$^{Arf-/-}$ mice. The data were analyzed using the unpaired Student's *t* test. The data are shown as the mean ± SEM, n = 5-6. $*p < 0.05$, $**p < 0.01$. Bregs – B regulatory cells, MASLD – metabolic dysfunction-associated steatotic liver disease, PBs –plasmablasts.

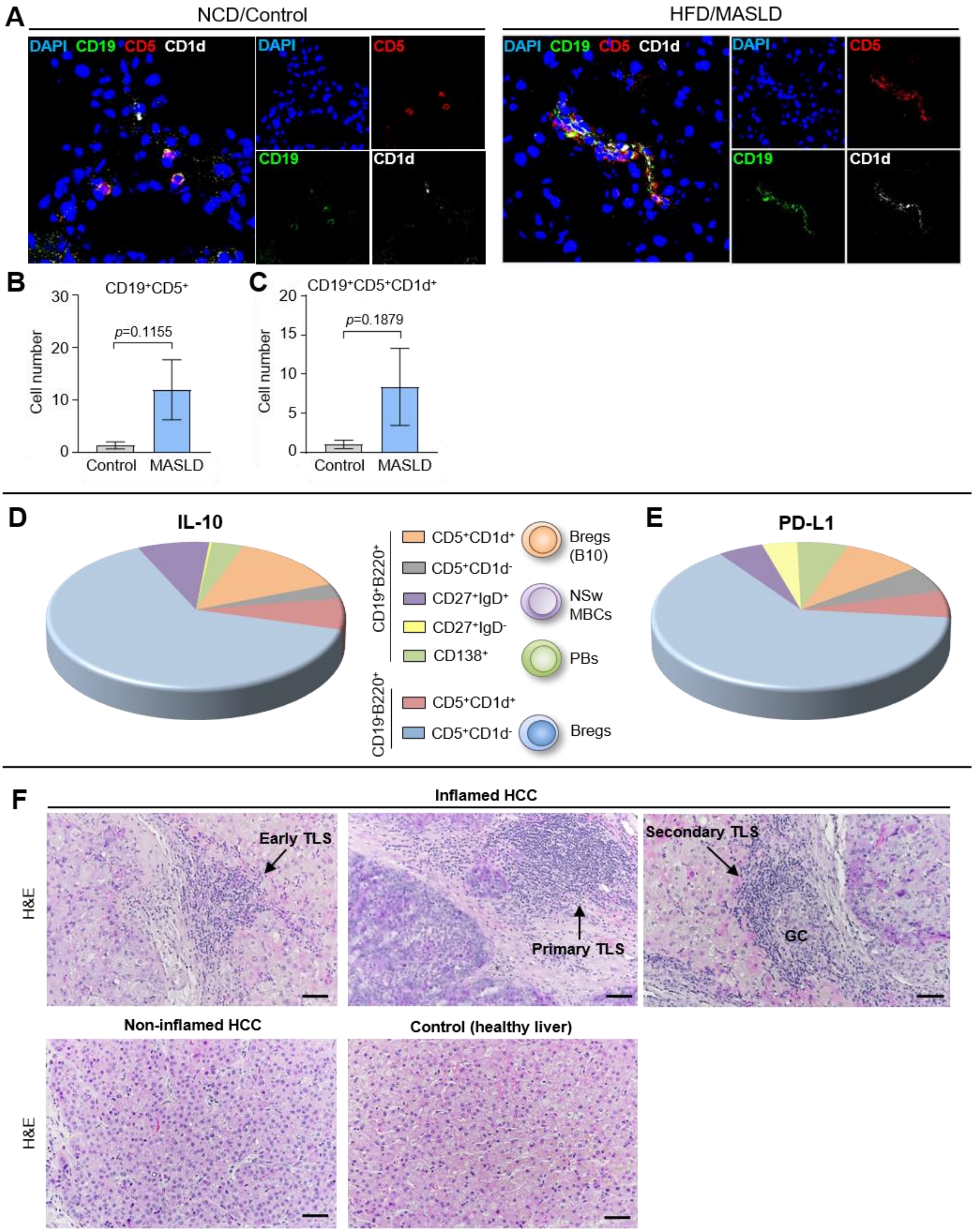

Fig. S6. An increase in the numbers of CD19⁺CD5⁺, CD19⁺CD5⁺CD1d⁺ Breg cells in murine MASLD; comparison of B cell subsets based on IL-10 and PD-L1 expression in murine HCC/*CaMIN*; TLS structures in murine HCC. (A) Representative IF images on frozen liver sections were obtained from MASLD mice stained with CD19 (green), CD5 (red), and CD1d (white) antibodies and counterstained with DAPI (blue). **(B-C)** Quantification of **(B)** $CD19^{+}CD5^{+}$, and **(C)** $CD19^{+}CD5^{+}CD1d^{+}$ cells in the livers obtained from MASLD mice. (**D-E)**

Pie charts demonstrating the proportions of **(D)** IL-10$^+$, and **(E)** PD-L1$^+$ B cell populations in the livers of mice with HCC/*CaMIN*. **(F)** TLS detection in H&E-stained human HCC, demonstrating early, primary, and secondary TLS types and the presence of a germinal center in the inflamed HCC tissue. Controls: non-inflamed HCC (on the left) and healthy liver tissue (on the right). Magnification 400x; scale bar, 100 µm. The data were analyzed using the unpaired Student's *t* test. The data are shown as the mean ± SEM, n = 5-6. NCD – normal chow diet, HFD – high-fat diet, NSw MBC – non-switched memory B cell, Breg – B regulatory cell, PB – plasmablast, TLS – tertiary lymphoid structures, GC – germinal center, MASLD – metabolic dysfunction-associated steatotic liver disease.

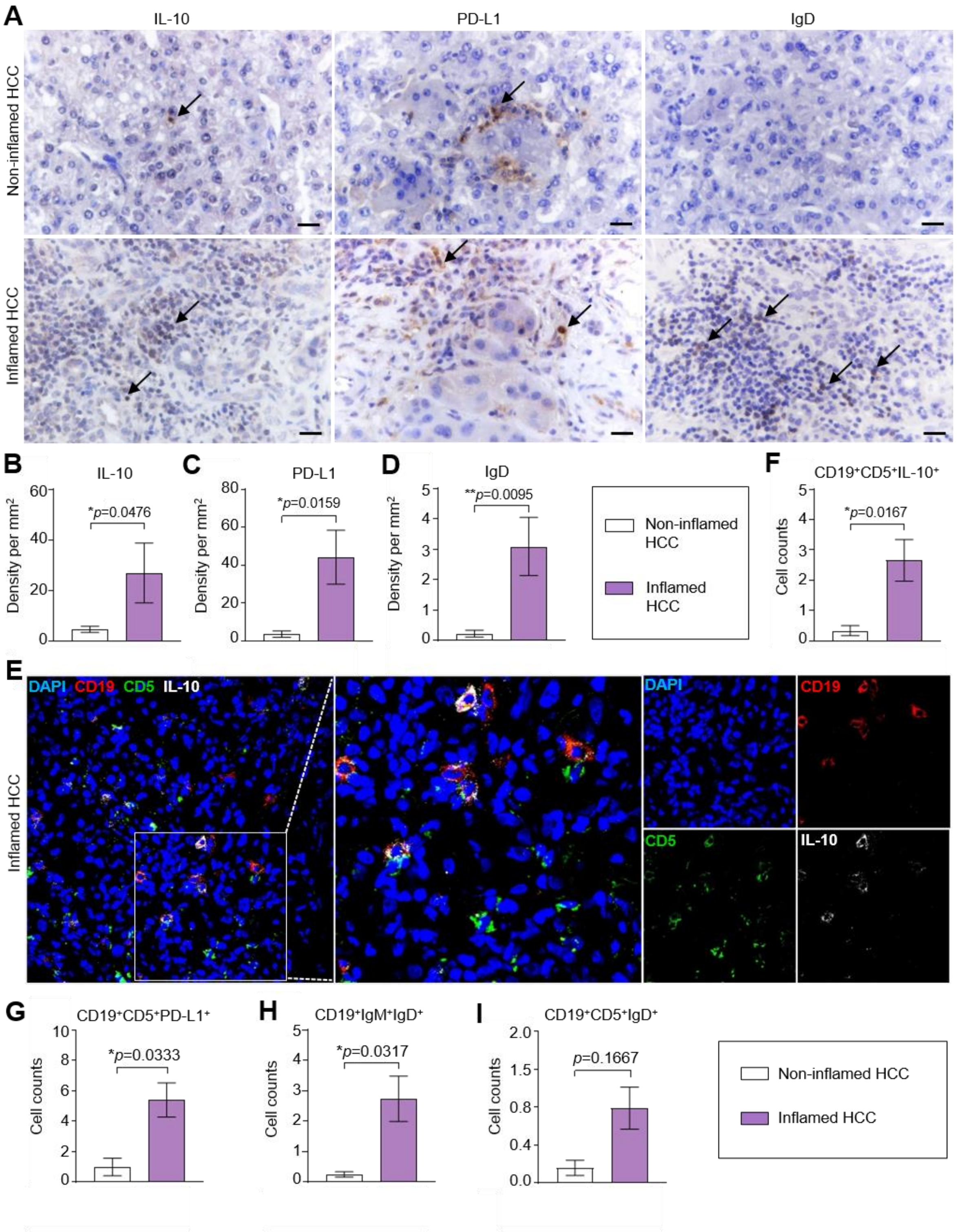

Fig. S7. The inflamed subtype of human HCC is characterized by the increased numbers of $CD19^{+}CD5^{+}IL\text{-}10^{+}$, $CD19^{+}CD5^{+}PD\text{-}L1^{+}$, and $CD19^{+}IgM^{+}IgD^{+}$ B cells. (A) Representative images of IHC of IL-10, PD-L1, and IgD expression in the non-inflamed and inflamed human HCC tissues. Scale bar, 100 µm. **(B-D)** Density of cellular markers **(B)** IL-10, **(C)** PD-L1, and **(D)** IgD in the non-inflamed and inflamed human HCC tissues. The data were analyzed using the Mann–Whitney nonparametric test, n=10. $*p < 0.05$, $**p < 0.01$. **(E)** Representative IF images on frozen liver sections from patients with inflamed HCC stained

with CD19 (red), CD5 (green), and IL-10 (white) antibodies and counterstained with DAPI (blue). **(F-I)** Quantification of **(G)** $CD19^{+}CD5^{+}IL\text{-}10^{+}$, **(G)** $CD19^{+}CD5^{+}PD\text{-}L1^{+}$, **(H)** $CD19^{+}IgM^{+}IgD^{+}$, and **(I)** $CD19^{+}CD5^{+}IgD^{+}$ cells in the non-inflamed and inflamed human HCC tissues. The data were analyzed using the Mann–Whitney nonparametric test, n=7. *$p < 0.05$.

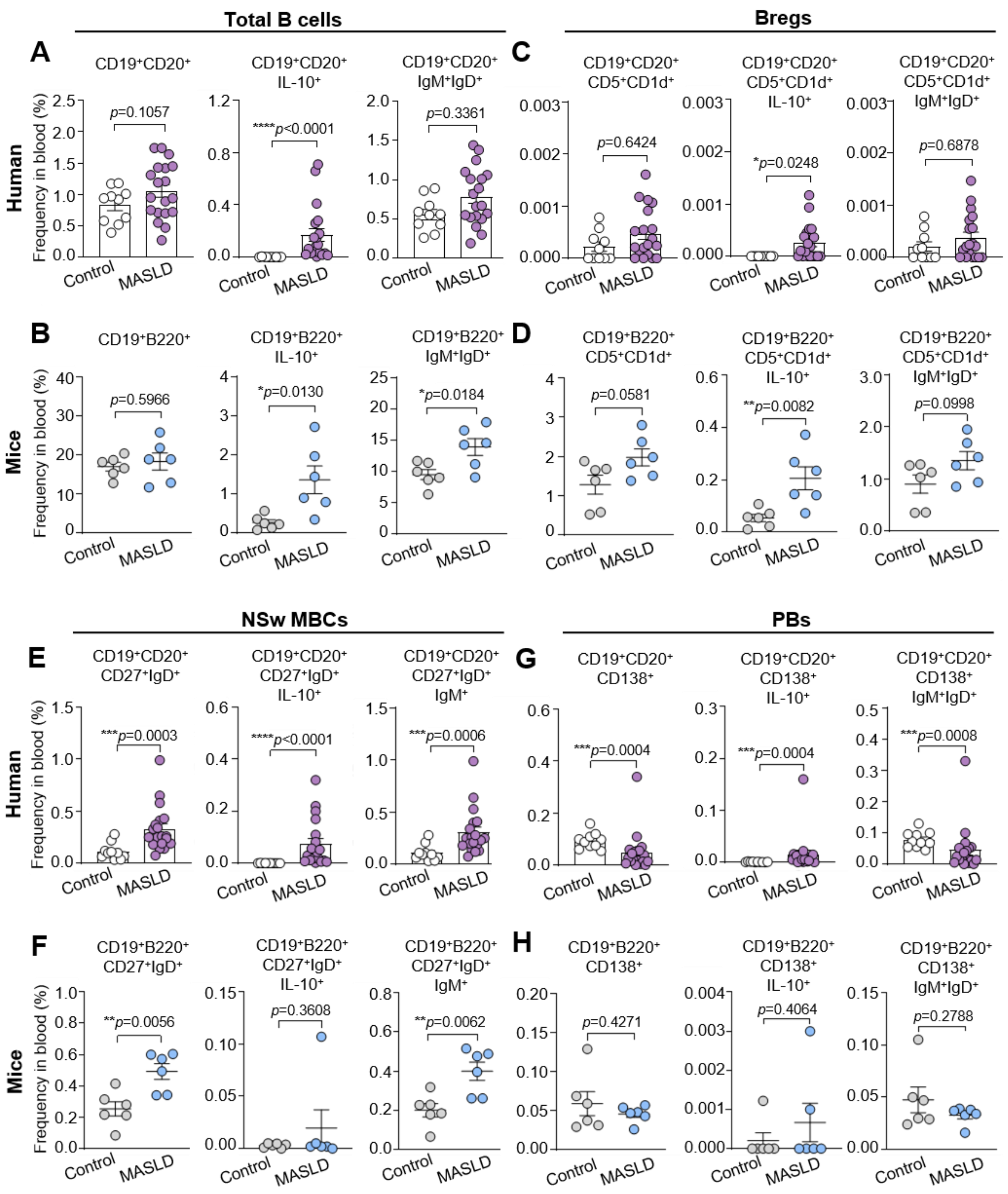

Fig. S8. B cell phenotyping in the blood of patients with MASLD revealed a strong increase of IL-10 on total $CD19^{+}CD20^{+}$ B cells, on $CD19^{+}CD20^{+}CD5^{+}CD1d^{+}$ Bregs and on $CD19^{+}CD20^{+}CD27^{+}IgD^{+}$ NS MBCs. The data strongly correlated between mice and humans with MASLD. (A, B) Frequencies of total B cells in the blood of **(A)** patients and **(B)** mice with MASLD. **(C, D)** Frequencies of Bregs in the blood of **(C)** patients and **(D)** mice with MASLD. **(E, F)** Frequencies of NSw MBCs in the blood of **(E)** patients and **(F)** mice with MASLD. **(G, H)** Frequencies of PBs in the blood of **(G)** patients and **(H)** mice with MASLD. The data were analyzed using the Mann–Whitney nonparametric test, n=4. $*p < 0.05$, $**p <$

0.01, ***$p < 0.001$, ****$p < 0.0001$. Bregs – B regulatory cells, NSw MBCs – non-switched memory B cells, PBs – plasmablasts. MASLD – metabolic dysfunction-associated steatotic liver disease.

Graphical abstract. Essential roles of IgM$^+$IgD$^+$ Bregs, NSw MBCs and PBs in the progression of MASLD and HCC.

Murine models were used to clarify the phenotypic characteristics and investigate the role of B lymphocytes in precancerous (MASLD) and cancerous (HCC) liver diseases. The results obtained in mice have also been verified in patients with MASLD and HCC. Blood and liver tissues obtained from mice and patients were analyzed using multicolor FACS, ELISA, IHC and IF. Our study revealed an increase in numbers of several specific B cell subsets: 1) CD19$^+$B220$^+$CD5$^+$CD1d$^+$ B10 Bregs; 2) CD19$^-$B220$^+$CD5$^+$CD1d$^-$ Bregs; 3) CD19$^+$B220$^+$CD5$^-$CD27$^+$ NSw MBCs; and 4) CD19$^+$B220$^+$CD138$^+$ PBs, all of which highly expressed IgM, IgD receptors, and the inhibitory molecules PD-L1 and IL-10. Two pie chart diagrams, demonstrating the proportions of IL-10$^+$- and PD-L1$^+$-expressing B cell subsets in the livers of HCC/*CaMIN* mice and depicting the most immunosuppressive B cell subset, CD19$^-$B220$^+$CD5$^+$CD1d$^-$ Bregs, in murine HCC. All four identified B cell subsets might play a protumorigenic role in precancerous (MASLD) and cancerous (HCC) liver disease progression.

Supplementary tables

Table S1. Clinicopathological characteristics of the human cohort with HCC (n=16).

**Age (years)**	
Median (range)	69.08 (50-82)
**Gender, n (%)**	
Male	11 (68.75%)
Female	5 (31.25%)
**BMI**	
<25	5 (31.25%)
≥25	9 (56.25%)
Unknown	2 (12.50%)
**AFP, n (%)**	
<20 ng/ml	8 (50.00%)
≥20 ng/ml	3 (18.75%)
Unknown	5 (31.25%)
**GGT, n (%)**	
<54 U/L	7 (43.75%)
8 (50.00%)	8 (50.00%)
Unknown	1 (6.25%)
**Microvascular invasion, n (%)**	
Yes	3 (18.75%)
No	13 (81.25%)
**Inflammatory type**	
Inflamed	8 (50.00%)
Non-inflamed	8 (50.00%)
**Tumor stage, TNM, n (%)**	
1	6 (37.50%)
2	4 (25.00%)
3	1 (6.25%)
4	1 (6.25%)
Unknown	4 (25.00%)
**Tumor grade, n (%)**	

I	0
II	12 (75.00%)
III	1 (6.25%)
Unknown	3 (18.75%)

BMI – body mass index; AFP – alpha-1-fetoprotein; GGT – gamma-glutamyltransferase; TNM – tumor, nodes, metastasis.

Table S2. Clinical characteristics of the human cohort with MASLD (n=19).

**Age (years)**	
Median (range)	48 (32-74)
**Gender, n (%)**	
Male	14 (73.68%)
Female	5 (26.32%)
**BMI**	
<25	18 (94.73%)
≥25	1 (5.26%)
**AFP, n (%)**	
<20 ng/ml	0 (0%)
≥20 ng/ml	19 (100%)
**GGT, n (%)**	
<54 U/L	11 (57.89%)
≥54 U/L	8 (42.10%)

BMI – body mass index; AFP – alpha-1-fetoprotein; GGT – gamma-glutamyltransferase.

Table S3. Key resources table.

Reagent or resource	Source	Identifier
**Antibodies used for flow cytometry**		
Anti-mouse B220	BioLegend	Cat # 103236;<br>RRID: AB_893354
Anti-mouse CD5	BioLegend	Cat # 100622;<br>RRID: AB_2562773
Anti-mouse CD1d	BioLegend	Cat # 123520;<br>RRID: AB_2715918
Anti-mouse PD-L1	BioLegend	Cat # 124343;<br>RRID: AB_2894674
Anti-mouse IgM	BioLegend	Cat # 406504;<br>RRID: AB_315054
Anti-mouse IgD	BioLegend	Cat # 405704;<br>RRID: AB_315026
Anti-mouse CD27	BioLegend	Cat # 124233;<br>RRID: AB_2687192
Anti-mouse CD138	BioLegend	Cat # 142515;<br>RRID: AB_2562336
Anti-mouse CD267	BioLegend	Cat # 133404;<br>RRID: AB_2240584
Anti-mouse IL-10	BioLegend	Cat # 505022;<br>RRID: AB_2563240
Anti-mouse CD19	BD Bioscience	Cat # 563157;<br>RRID: AB_2738035
Anti-mouse IgA	eBioscience	Cat # 13599482;<br>RRID: AB_466863
Anti-mouse CD20	BioLegend	Cat # 152104;<br>RRID: AB_2629619
Anti-human CD3	BioLegend	Cat # 344846;<br>RRID: AB_2800923
Anti-human CD11c	BioLegend	Cat # 301626;<br>RRID: AB_10662381
Anti-human CD19	BioLegend	Cat # 302270;<br>RRID: AB_2832581
Anti-human CD20	BioLegend	Cat # 302332;<br>RRID: AB_2563805
Anti-human CD5	BioLegend	Cat # 364020;<br>RRID: AB_2565941
Anti-human CD1d	BioLegend	Cat # 350316;<br>RRID: AB_2687379
Anti-human CD38	BioLegend	Cat # 303550;<br>RRID: AB_2860784
Anti-human CD27	BioLegend	Cat # 356428;<br>RRID: AB_261671
Anti-human IgM	BioLegend	Cat # 314544;

		RRID: AB_2800832
Anti-human PD-L1	BioLegend	Cat # 329718; RRID: AB_2561687
Anti-human IL-10	BioLegend	Cat # 501426; RRID: AB_2566744
Anti-human CD45	BD Biosciences	Cat # 563792; RRID: AB_2869519
Anti-human CD24	BD Biosciences	Cat # 741831; RRID: AB_2871166
Anti-human IgD	BD Biosciences	Cat # 566138; RRID: AB_2739536
TruStain FcX™ (anti-mouse CD16/32) antibody	BioLegend	Cat # 101320; RRID: AB_1574975
Human TruStain FcX™ (Fc Receptor Blocking Solution)	BioLegend	Cat # 422302; RRID: AB_2818986
BV785 Streptavidin	BioLegend	Cat # 405249; RRID: N/A
APC-Cy7 Streptavidin	BioLegend	Cat # 405208; RRID: N/A
**Antibodies used for IHC and IF**		
Anti-CD19	Invitrogen	Cat # PA5-27442; RRID: AB_2544918
Anti-CD19	eBioscience™	Cat # 14019482; RRID: AB_2637171
Anti-CD19	eBioscience™	Cat # 53-0194-82; RRID: AB_2637167
Anti-CD5	Epredia™Lab Vision™	Cat # MS-393-R7; RRID: AB_61248
Anti-CD5	Invitrogen	Cat # MA5-17781; RRID: AB_2539165
Anti-CD5	Santa Cruz Animal Health	Cat # sc-1180; RRID: AB_627112
Anti-CD1d	antibodies-online.com	Cat # ABIN3022497; RRID: N/A
Anti-IL-10	Invitrogen	Cat # PA5-85660; RRID: AB_2792799
Anti-PD-L1	Invitrogen	Cat # PA5-28115; RRID: AB_2545591
Anti-IgD	Abcam	Cat # AB124795; RRID: AB_10974228
Anti-IgM	Santa Cruz Animal Health	Cat # sc-53347; RRID: AB_672096
Donkey anti-rat IgG, Alexa Fluor 647	Invitrogen	Cat # A-78947; RRID: AB_2910635
Donkey anti-rabbit IgG, Alexa Fluor 555	Invitrogen	Cat # A-31572; RRID: AB_162543
Donkey anti-mouse IgG, Alexa Fluor 488	Invitrogen	Cat # A-21202; RRID: AB_141607

Goat anti-mouse IgG1,<br>Alexa Fluor 488	Invitrogen	Cat # A-21121;<br>RRID: AB_2535764
Goat anti-rat IgG,<br>Alexa Fluor 568	Invitrogen	Cat # A-11077;<br>RRID: AB_2534121
Goat anti-rabbit IgG,<br>Alexa Fluor 647	Invitrogen	Cat # A-21245;<br>RRID: AB_2535813
**Antibodies used for B-cell depletion**		
Anti-mouse CD20	BioLegend	Cat # 152116;<br>RRID: AB_2629619
**Chemicals, peptides and recombinant proteins**		
Complete DMEM (cDMEM) medium	Gibco	Cat # 31966021
Complete RPMI 1640 (cRPMI) medium	Gibco	Cat # 72400047
Fetal bovine serum (FBS)	Serena	Cat # S-FBSP-EU-015
Penicillin/Streptomycin	Gibco	Cat # 15070063
Collagenase D	Roche	Cat # 11088882001
DNAse I	Sigma Aldrich	Cat # D4527
EDTA	Carl Roth	Cat # 8043.2
Heparin 5000	Ratiopharm	Cat # PZN-03029820
Ammonium chloride (NH_4Cl)	Carl Roth	Cat # P726.2
Potassium hydrogen carbonate ($KHCO_3$)	Carl Roth	Cat # X887.2
Phosphate buffered saline (PBS)	Gibco	Cat # 70013-016
Ficoll-Paque PLUS	GE Healthcare	Cat # GE17-1440-02
Trypan blue	Sigma Aldrich	Cat # T815
Alexa Fluor™ 350 NHS Ester (Succinimidyl Ester)	Life Technologies (Molecular Probe)	Cat # 11579036
Argentum	Honeywell-Fluka	Cat # 31630-2506
Eosin	Merck	Cat # 1.115935-0100
Hematoxylin	Sigma Aldrich	Cat # 517-28-2
Sirius red (Direct Red 80)	Sigma Aldrich	Cat # 2610-10-8
Oil red O	Sigma Aldrich	Cat # O-0625
DAPI (4’,6-diamidino-2-phenylindole)	Sigma-Aldrich	Cat # D9542-50MG
Normal goat serum	Abcam	Cat # ab7481
Bovine serum albumin fraction V	Carl Roth	Cat # 8076.4
Triton X-100	Sigma Aldrich	Cat # 9002-93-1

Sodium citrate dihydrate	Fisher Scientific	Cat # 11945071
**Commercial kits / assays**		
QIAGEN EndoFree Maxi Kit	Qiagen	Cat # 12362
Mouse Immunoglobulin D (IgD) ELISA Kit	CusaBio	Cat # CSB-E15761m
Epredia™ UltraVision™ Quanto Detection System HRP DAB	Fisher Scientific	Cat # 12673997
**Experimental models: animals / murine strains**		
C57BL/6J	Charles River	Strain code: 632; RRID: IMSR_JAX:000664
B6.129X1-Cdkn2a^{tm1Cjs} (*p19*$^{Arf-/-}$)	Jackson Laboratory	Strain code: 029676; RRID: IMSR_JAX:029676
B6.129S2-Igh-6$^{tm1Cgn/J}$ (µMT)	Jackson Laboratory	Strain Code: 002288; RRID: IMSR_JAX:002288
B6.129P2-Igh-J$^{tm1Cgn/J}$ (JHT)	Jackson Laboratory	Strain Code: 002438; RRID: IMSR_JAX:002438
**Software and algorithms**		
FlowJo, v10.8.1	Tree Star Inc.	RRID: SCR_008520
GraphPad Prism, v8.3	GraphPad Software	RRID: SCR_002798
ZEN Digital Imaging for Light Microscopy, 2011	Carl Zeiss AG	RRID: SCR_013672
Fiji/ImageJ	Fiji	RRID: SCR_002285
Adobe Photoshop CS5	Adobe	RRID: SCR_014199
**Other**		
Instrument: Cytek Aurora	Cytek	
Instrument: LSR II	BD Biosciences	
Instrument: LUNA-FL™ Automated Fluorescence Cell Counter	Logos Biosystems	
Instrument: Microtome HM 335 E	Microm	
Instrument: Cryotome HM 500 OM	Microm	
Instrument: Zeiss LSM 700 confocal laser-scanning microscope	Carl Zeiss AG	
Instrument: BX51, Olympus	Olympus	
Nikon Eclipse Ti2 Inverted Microscope	Nikon	

<u>Supplementary references</u>

Author names in bold designate shared co-first authorship.

[1] Kamijo T, Zindy F, Roussel MF, et al. Tumor suppression at the mouse INK4a locus mediated by the alternative reading frame product p19ARF. Cell 1997;91:649-659.

[2] Dauch D, Rudalska R, Cossa G, et al. A MYC-aurora kinase A protein complex represents an actionable drug target in p53-altered liver cancer. Nature medicine 2016;22:744-753.

[3] **Kang TW, Yevsa T,** Woller N, et al. Senescence surveillance of pre-malignant hepatocytes limits liver cancer development. Nature 2011;479:547-551.

[4] Kitamura D, Roes J, Kuhn R, at al. B cell-deficient mouse by targeted disruption of the membrane exon of the immunoglobulin mu chain gene. Nature 1991;350:423-426.

[5] Gu H, Zou YR, Rajewsky K. Independent control of immunoglobulin switch recombination at individual switch regions evidenced through Cre-loxP-mediated gene targeting. Cell 1993;73:1155-1164.

[6] Ohashi T, Nakade Y, Ibusuki M, et al. Conophylline inhibits high fat diet-induced non-alcoholic fatty liver disease in mice. PloS one 2019;14:e0210068.

[7] **Bartolome F, Antequera D, de la Cueva M,** et al. Endothelial-specific deficiency of megalin in the brain protects mice against high-fat diet challenge. Journal of neuroinflammation 2020;17:22.

[8] **Petriv N, Neubert L,** Vatashchuk M, et al. Increase of alpha-dicarbonyls in liver and receptor for advanced glycation end products on immune cells are linked to nonalcoholic fatty liver disease and liver cancer. Oncoimmunology 2021;10:1874159.

[9] Shalapour S, Lin XJ, Bastian IN, et al. Inflammation-induced IgA+ cells dismantle anti-liver cancer immunity. Nature 2017;551:340-345.

[10] DiLillo DJ, Hamaguchi Y, Ueda Y, et al. Maintenance of long-lived plasma cells and serological memory despite mature and memory B cell depletion during CD20 immunotherapy in mice. J Immunol 2008;180:361-371.

[11] **Hochnadel I, Hoenicke L,** Petriv N, et al. Safety and efficacy of prophylactic and therapeutic vaccine based on live-attenuated Listeria monocytogenes in hepatobiliary cancers. Oncogene 2022.

[12] **Rudalska R, Dauch D,** Longerich T, et al. In vivo RNAi screening identifies a mechanism of sorafenib resistance in liver cancer. Nature medicine 2014;20:1138-1146.

[13] Eggert T, Wolter K, Ji J, et al. Distinct Functions of Senescence-Associated Immune Responses in Liver Tumor Surveillance and Tumor Progression. Cancer cell 2016;30:533-547.

[14] Neubert L, Borchert P, Stark H, et al. Molecular Profiling of Vascular Remodeling in Chronic Pulmonary Disease. The American journal of pathology 2020;190:1382-1396.

[15] Neubert L, Borchert P, Shin HO, et al. Comprehensive three-dimensional morphology of neoangiogenesis in pulmonary veno-occlusive disease and pulmonary capillary hemangiomatosis. The journal of pathology Clinical research 2019;5:108-114.

[16] Mehlem A, Hagberg CE, Muhl L, at al. Imaging of neutral lipids by oil red O for analyzing the metabolic status in health and disease. Nature protocols 2013;8:1149-1154.

[17] Sia D, Jiao Y, Martinez-Quetglas I, et al. Identification of an Immune-specific Class of Hepatocellular Carcinoma, Based on Molecular Features. Gastroenterology 2017;153:812-826.

[18] **Montironi C, Castet F, Haber PK,** et al. Inflamed and non-inflamed classes of HCC: a revised immunogenomic classification. Gut 2023;72:129-140.
